# Social media activities to enhance the visibility of long-term invasive ventilation: A content and visibility analysis from the PRiVENT study

**DOI:** 10.1371/journal.pone.0352267

**Published:** 2026-06-25

**Authors:** Franziska C. Trudzinski, Nicola Litke, Olaf Buntemeyer, Simone Janssen, Elena Biehler, Thomas Fleischhauer, Jana C. Dahlhoff, Philipp Höger, Axel Kempa, Benjamin Neetz, Biljana Joves, Claus Neurohr, Alessandro Ghiani, Armin Schneider, Joachim Szecsenyi, Jan Meis, Janina Schubert-Haack, Julia D. Michels-Zetsche, Felix JF Herth, and the PRiVENT-Study Group

**Affiliations:** 1 Thoraxklinik University Hospital Heidelberg, Department of Pneumology and Critical Care, Heidelberg, Translational Lung Research Centre Heidelberg (TLRC-H), Member of the German Centre for Lung Research (DZL), Heidelberg, Germany; 2 Department of General Practice and Health Services Research, University Hospital Heidelberg, Heidelberg, Germany; 3 Visuelle Werte GmbH, Berlin, Germany; 4 SLK-Klinik Löwenstein, Department of Pneumology and Critical Care, Löwenstein, Germany; 5 Robert-Bosch-Krankenhaus Klinik Schillerhöhe, Department of Pneumology and Respiratory Medicine, Gerlingen, Germany; 6 Department of Anesthesia and Intensive Care Medicine Waldburg-Zeil Kliniken, Wangen im Allgäu, Germany; 7 aQua Institute for Applied Quality Improvement and Research in Health Care, Göttingen, Germany; 8 Institute of Medical Biometry, Heidelberg University, Heidelberg, Germany; IESEG School of Management, FRANCE

## Abstract

**Background:**

Despite the increasing number of patients receiving long-term invasive mechanical ventilation (IMV) and the social importance of this issue, it is largely neglected in the public perception. One aim of the PRIVENT project was to raise public awareness of the issue.

**Methods:**

A team of physicians, health researchers, an art director and a scientific editor developed an information campaign, including blog posts, podcasts, social media posts and information for medical professionals or patients/their relatives and made them available to the public on a dedicated homepage and various social media platforms. The number of views was used to measure the reach of the campaign.

**Results:**

In the period from 02/2021–03/2025, a total of 72 blog posts and 880 social media posts and 3 podcast seasons with 21 episodes were made available to the public. The total reach was 2.6 million, with Facebook, Instagram and LinkedIn being the most used platforms with a total of 1872 followers. The most popular blog posts were those that explained the basics of IMV in plain language. Followers of the different social networks differ. Instagram users are younger and more likely to be female than those on Facebook and LinkedIn.

**Conclusion:**

Our analysis shows that social media can increase the visibility of long-term invasive mechanical ventilation and reach diverse audiences. Although visibility does not equate to awareness, these findings highlight the potential of social media to support broader communication about long-term IMV and weaning within the medical community and society. Trial registration number: The PRiVENT study is registered at ClinicalTrials.gov (NCT05260853) on 02/03/2022.

## Introduction

The increasing number of patients being discharged with invasive mechanical ventilation (IMV) is the downside of modern intensive care medicine in Germany [1]. For these patients and their families, transitioning to home mechanical ventilation (HMV) represents a significant lifestyle change and reduction in independence. Patients on HMV usually require round-the-clock intensive care. The existing shortage of qualified staff in intensive care medicine is well known, and recruiting well-trained staff to provide outpatient care will only worsen the problem. The resulting costs are a significant burden on the health care system and thus on the general public [2,3]. Nevertheless, this topic is hardly addressed in the public perception. We suspect there are several reasons for this: The healthy do not usually want to deal with such an unpleasant subject because they assume that it will not affect them. Patients with chronic lung disease who are at risk of long-term ventilation, on the other hand, suppress the topic even more, as it is particularly painful and associated with great anxiety. In addition, those affected are often severely disabled and are unable to express their thoughts and feelings to the public due to their severe impairments, of which the tracheotomy is often only one aspect. HMV patients and their families often struggle to manage their daily lives and have few opportunities to participate in social life [4]. In addition to the general public, the topic of long-term ventilation primarily concerns staff in intensive care units, who are frequently confronted with it but have considerable gaps in their knowledge about weaning from mechanical ventilation [5–7].

Social media has become an important tool in digital health communication, offering new ways to disseminate specialised medical information to diverse audiences. Social media has become an increasingly important component of digital health communication, enabling healthcare organisations and research projects to disseminate information, engage stakeholders, and increase the visibility of health-related topics. From a health information-seeking perspective, digital platforms provide low-threshold access to information for patients, relatives, healthcare professionals, and the general public. At the same time, concepts of digital engagement suggest that communication effectiveness may vary across platforms because different audiences interact with health information in different ways. Understanding how specialised healthcare topics are communicated and received across digital channels is therefore relevant for both public health communication and the dissemination of healthcare research.

The German project PRiVENT is investigating innovative care methods for patients receiving invasive mechanical ventilation [8]. One of the project’s objectives is to raise public awareness of the issue of long-term ventilation and to empower those affected and their families. This will be achieved by providing interesting and targeted information on various topics related to invasive ventilation and inpatient and out-of-hospital care for long-term ventilated patients via social media. The aim of this study is to analyse the visibility and reach of the project’s social media activities across the platforms used.

## Methods

Within the project PRiVENT a secondary aim was to enhance the visibility of the topics of weaning, invasive ventilation, and inpatient and outpatient care for long-term ventilated patients. The present study analyses the visibility and reach of this campaign using platform-provided metrics. All content was published in German. The study was approved by the ethics committee of the Medical Faculty Heidelberg (S-352/2018).

### Content production

#### The PRiVENT public relations team.

The content of the social media campaign within the PRiVENT project was created by a multi-professional team consisting of a pulmonologist and intensive care physician, health researchers as well as advertising and knowledge communication experts, an art director and a scientific writer. All topics of the contributions were coordinated in a weekly virtual editorial conference in which the whole team took part.

### Project homepage and social media platforms

The heart of the campaign is the project homepage *“www.wieder-selbst-atmen.de”*, which went online in February 2021 and provides access to almost all content and links to the other social media channels. Project accounts were opened for the social media platforms Facebook, Instagram, the video platform YouTube, the career network LinkedIn, and the community for doctors, medical assistants, pharmacists, and other healthcare professionals DocCheck.

### Podcasts

The content of the podcasts was planned during the editorial meetings, with each episode aiming to be between 20 and 30 minutes long. In order to create thematically coherent podcast seasons, the topics were planned in advance for each entire season and inter-seasonal. Each episode was structured using a semi structured interview guideline. Lead questions were sent to the participants in advance. The requested guests received no honorarium for their participation. Whenever possible, the moderated podcast episodes were filmed on location with guests and the moderator at the Thoraxklinik in Heidelberg. In cases where the organisational effort for this was too great, the guests were connected online. The recorded interviews were then edited and reviewed by the entire team. Subtitling was required for one episode because the invited guest was difficult to understand due to his non-invasive ventilation. The finished podcast episodes were promoted on social media platforms and made available through YouTube, Deezer, Google Podcasts, Apple Podcasts, and Spotify video and audio services.

### Blog posts

In the editorial meetings, the topics of the blog posts were also agreed upon; these were written in plain language by a scientific editor and proofread by the collected team prior to publication. The contributions include various aspects of invasive mechanical ventilation, such as the tasks of the different professional groups in an intensive care unit or weaning unit, background information and resources on scientific publications and the published podcasts.

### Social media posts

In addition, social media posts were published on a regular basis via Facebook, Instagram, YouTube, LinkedIn and DocCheck to highlight current developments in the project as well as blog posts and podcasts. In order to establish a lively network and integrate different professional groups involved in the weaning process, various PRiVENT project partners were introduced and team photos of staff from the participating ICUs were displayed. The contributions were actively promoted on Facebook between 04/2021 and 12/03/2025.

### Target group definition (campaign design)

For targeted advertising, Facebook tools were used to define audiences aged 18–65 years. Criteria included interests and professional characteristics related to [1] patients and relatives (e.g., quality of life, health and social care) and [2] healthcare professionals (e.g., nursing professions, healthcare sector). Advertising was geographically restricted to Baden-Württemberg.

### Analytical procedures

#### Content reach.

Content reach was assessed using platform-provided metrics, including views, impressions, and interactions. In this study, “reach” refers to views (i.e., the number of times content was seen), while impressions reflect the total number of displays, including repeated views. Engagement metrics included interactions such as reactions and comments. User characteristics were derived from aggregated platform analytics (Facebook, Instagram, LinkedIn) to obtain descriptive and indirect indicators of audience demographics.

### Statistical analysis

Data on content volume and associated metrics were extracted on March 12, 2025. Analyses were conducted descriptively. Results are reported as absolute numbers and percentages for overall reach, as well as stratified by platform and content type.

## Results

In the period from 02/2021–03/2025, a total of 72 blog posts, 880 social media posts and 3 podcast seasons with a total of 21 episodes were made available to the public.

### Content of the contributions

**Fig 1:**
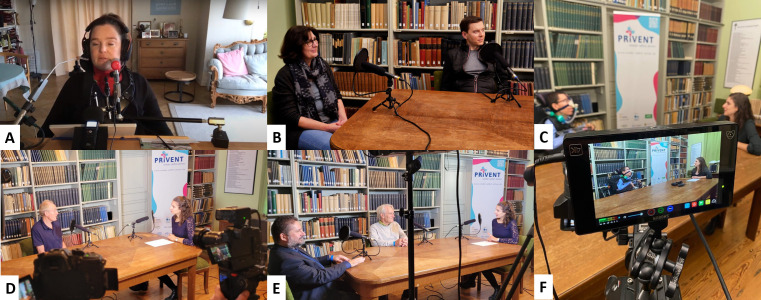
The PRiVENT Podcast Series. The illustration shows various interview situations from the PRiVENT podcast. In addition to patients **(A, D)**, respiratory therapists **(B)**, respiratory physicians (C) and representatives of patient support organisations, a number of other stakeholders were interviewed to give an insight into the situation of long-term respiratory care. Wherever possible, the recordings were made in the library of the Tuberculosis Museum of the Thoraxklinik in Heidelberg If on-site recording was not possible, interviews were conducted via video conference.

The aim of the podcasts was to give a differentiated impression of the current situation of patients on long-term mechanical ventilation and to highlight it from different perspectives. For this purpose, invasively and non-invasively ventilated patients, their relatives, representatives of self-help groups, doctors, respiratory therapists, nursing staff and employees of the largest statutory health insurance in Germany, the AOK, had the opportunity to present on the podcasts. **Table 1** indicates the topics of all podcasts published to date (03/2025). The themes discussed ranged from the history of ventilation to the current situation in intensive care units during the COVID-19 pandemic. **Fig 1** shows impressions from the interviews. The blog posts related to the content of the podcasts and provided background information. They also covered general topics for people with respiratory diseases, such as information on topics like “Apps that help manage respiratory diseases”, “How to motivate yourself to do breathing exercises?” and “Pulmonary rehabilitation: effective training for different pulmonary conditions”. There was also specific information on acute intensive care, such as “Animals in intensive care: more than just a fluffy visit”, “Children as relatives – when parents are in intensive care” or “Artificial intelligence in intensive care”. Other topics included weaning from mechanical ventilation, such as “What does ‘weaning’ mean and how does it work?” or “Who works on a ventilator weaning unit?”, and the care situations of patients with HMV, such as “Self-determined life with mechanical ventilation” and “Ventilation in children and the role of nursing”, as well as topics relating to aftercare in the ICU and the sequelae of intensive care, such as “Post-COVID syndrome – the difference between recovered and healthy”.

**Table 1:** Topics of the published podcasts.

Season/Episode	Title	Access numbers
Season 1 #1	How the PRiVENT project aims to improve ventilator weaning	148
Season 1 #2	How PRiVENT will improve the situation of patients on mechanical ventilation	85
Season 1 #3	When do people need ventilation and what are the risks?	102
Season 1 #4	Special episode – How the Corona pandemic affects work in intensive care units	70
Season 1 #5	How can patient support organisations support people on mechanical ventilation?	73
Season 1 #6	The importance and effectiveness of self-help and comprehensive patient education	61
Season 1 #7	How do certified weaning centres work and what are the benefits for patients?	93
Season 2 #1	Update on the study	55
Season 2 #2	History of Mechanical Ventilation	78
Season 2 #3	chronic obstructive pulmonary disease	84
Season 2 #4	The role of the AOK Baden-Württemberg in PRiVENT	68
Season 2 #5	What do respiratory specialists do?	73
Season 2 #6	Post COVID-Syndrome – The difference between recovered and healthy	83
Season 3 #1	How intensive care units and weaning centres work together	66
Season 3 #2	Quality Circles in the Weaning Process	69
Season 3 #3	Invasive mechanical ventilation in children and the role of Nursing	67
Season 3 #4	Self-determined life with mechanical ventilation	59
Season 3 #5	A plea for more patience and mutual understanding	61
Season 3 #6	In a wheelchair at 18 and 30 years later	64
Season 3 #7	What’s new in the PRiVENT project	40
Season 3 #8	An interview with Prof Dr Bernd Schönhofer	162

**Fig 2:**
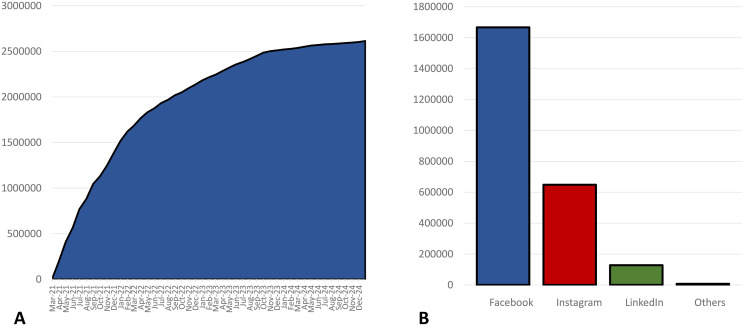
Content reach. **A)** Development of cumulative content reach over time from March 2021 to March 2025, based on platform-provided visibility metrics. Peaks in reach reflect periods of increased communication activity and/or particularly visible content. **B)** Total reach by platform during the observation period. Facebook and Instagram generated the highest overall reach, whereas LinkedIn contributed primarily to professional visibility. Reach refers to the number of content views reported by the respective platforms.

The blog posts provide information about the PRiVENT-project and illuminate the topic of “invasive long-term ventilation” from various perspectives. In the 20-minute videos, patients, hospital staff (pneumologists, intensive care physicians, and respiratory therapists), patient support groups, and health insurance companies describe their views on long-term invasive ventilation, thus providing an insight into the complex topic. Image excerpts from various interview situations are shown in Fig 2.

### Content reach

The total reach was 2.6 million views with Facebook and Instagram being the most used platforms with 1.7 and 0.6 million views, respectively, and a total of 1018 followers. The third most popular platform was LinkedIn, where 854 people are currently following the content. Visibility and reach were not evenly distributed over time but were characterised by several pronounced peaks, particularly on Facebook and Instagram. These fluctuations likely reflect periods of intensified communication activity, or content that generated above-average user engagement. In contrast, LinkedIn showed lower overall reach but contributed to a more continuous professional presence throughout the project period. **Fig 2** shows the evolution of total reach and provides an overview of the reach of the different platforms.

### The top 10 most popular pages by number of hits

With 161462 views from 74751 visitors, the most popular online contributions to the project were those that explained the basics of weaning and mechanical ventilation in simple language. The most popular post was entitled “What is weaning and how does it work” (35617 views), followed by “What is invasive ventilation” (27236 views) and “Respiratory trainers in physiotherapy and respiratory therapy” (10438 views). **Table 2** shows the topics and access figures for the 10 most popular posts.

**Table 2:** The top 10 most popular blog posts (accessed 03/2025).

Most popular pages by number of visits	Type	Views
What does “weaning” mean and how does weaning work?	Blog post	35617
What is invasive mechanical ventilation?	Blog post	27236
Respiratory trainers in physiotherapy and respiratory therapy	Blog post	10438
“What is a residential community for HMV patients and why have these facilities been criticised?”	Blog post	9583
Exercise to strengthen and stretch the lateral respiratory muscles	Blog post	7115
physical activity against breathlessness during exercise	Blog post	4268
Lungs and respiratory pump: the two parts of the respiratory system	Blog post	3175
What do respiratory therapists do?	Blog post	3162
The PRiVENT e-learning programme	Specialist information	1438
PRiVENT download material	Specialist information	1241

### Users of the different platforms

**Fig 3:**
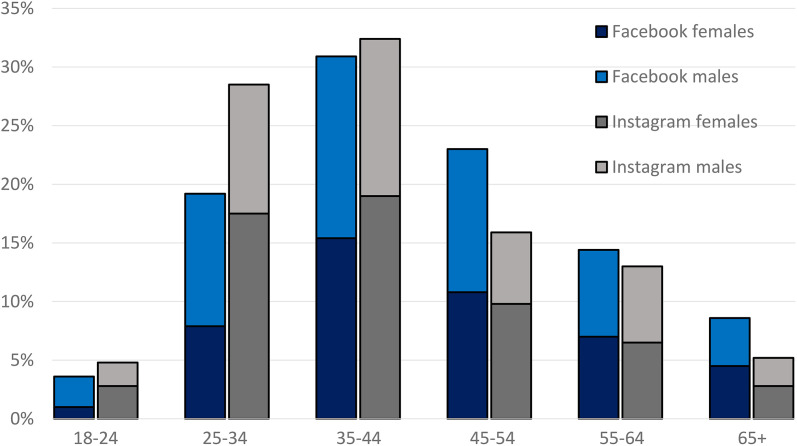
Age and gender distribution of Facebook and Instagram followers. The figure shows the distribution of followers across age groups and gender categories based on platform-provided audience analytics for Facebook and Instagram. Values are presented as percentages of the respective platform audiences and illustrate differences in the demographic composition of followers between the two social media platforms.

Compared to Facebook, Instagram users were younger, with the majority aged between 25 and 30 years, compared to 35–44 years for Facebook. Instagram also had a higher proportion of female users, with 58% of users being female compared to 47% on Facebook (accessed 03/2025). The business network LinkedIn does not provide information about the sex and age of its users, but a review of user profiles shows that LinkedIn users were more likely to be men than on other platforms, with 38.3% describing themselves as experienced professionals, 8.4% as managers and 9.6% as directors. Recent graduates account for only 25.5% of the total, while interns and trainees account for 2.0%. The majority of followers worked in the healthcare sector, with 42.2% in healthcare, 9.5% in business development and 8.3% in research. Accordingly, 29.6% of followers worked in hospitals and healthcare facilities, 12.4% in medical practices and 10.5% in the manufacture of medical devices. 5.6% worked in pharmaceutical manufacturing and 4.9% in colleges and universities (accessed (03/2025). **Fig 3** provides an overview of the age and gender distribution of Facebook and Instagram users, while **Table 3** provides information on users of the professional network LinkedIn.

**Table 3:** LinkedIn followers (accessed 03/2025).

LinkedIn followers	Nr. (%) N = 856
*Area of professional activity*	
Health services	361 (42.2)
Distribution and sales	81 (9.5)
Business development	71 (8.3)
Scientific research	55 (6.4)
in training and education	36 (4.2)
Human Resources	19 (2.2)
Others	233 (27.2)
*Business sector*	
Hospitals and healthcare facilities	253 (29.6)
Medical practices	106 (12.4)
Manufacture of medical devices	90 (10.5)
Hospitals	59 (6.9)
Pharmaceutical production	48 (5.6)
Universities and colleges	42 (4.9)
Others	258 (30.1)
*Career level*	
professionally experienced	328 (38.3)
young professionals	218 (25.5)
director	82 (9.6)
manager	72 (8.4)
vice president	31 (3.6)
Internship/Training	17 (2.0)
others	108 (12.6)

## Discussion

The PRiVENT project aimed to enhance the visibility of information on long-term mechanical ventilation. In the current analysis, platform-provided metrics such as access numbers and user characteristics of the social media activities within the project were studied. Between February 2021 and March 2025, a range of web-based content was created and made available to the public via a dedicated website and various social media platforms. In total, 72 blog posts, 880 social media posts and 3 podcast seasons with a total of 21 episodes were provided, highlighting the topic from different perspectives. Platform-provided analytics suggest that different formats and channels reached distinct user groups; however, these metrics are limited to measures of visibility and reach and do not allow conclusions regarding user interest, informational needs, or cognitive engagement. It should be noted that this is a highly specialised topic, which naturally attracts smaller audiences compared with general lifestyle content. Our analysis showed that the most frequently accessed online posts were those explaining the basics of weaning and mechanical ventilation in plain language. The article “What is weaning and how does it work?” received 35,617 views, and “What is invasive ventilation?” reached 27,236 views. While high view counts cannot confirm the specific identities of the readers, they indicate substantial public interest and suggest that such foundational information is widely sought—likely including patients and relatives. The observed access patterns may suggest substantial public interest in easily understandable weaning-related information in Germany, although user motivations and information needs cannot be directly inferred from the available data. The involvement of patients and relatives, who are an important resource in the ICU recovery and weaning process [1,9,10], requires information and education. There is therefore a need for patients and relatives to understand intensive care therapies, for which they need objective and easily accessible sources of information, in addition to personal dialogue with the attending physicians. Another target group of the campaign was intensive care staff across participating units, with the aim of providing knowledge on weaning from mechanical ventilation. Nursing staff are a valuable resource in the weaning process as the active involvement of non-medical staff can shorten the duration of mechanical ventilation in ICU [11–13]. As part of the project, various measures were implemented to enhance nursing staff competence in ventilator weaning [8]. According to the demographic estimates provided by Facebook and Instagram analytics, the content on these platforms was viewed predominantly by younger and female users. In addition to bedside consultations, during which pneumologists and respiratory therapists from the weaning centres discussed complex patient cases with staff at cooperating clinics [13]. Additionally, an e-learning programme was offered to all professional groups involved [14]. The effects of the weaning consultations were explored in qualitative interviews, which suggested that nursing staff had limited opportunities to participate in these consultations because offstaff shortages [15].

Digital dissemination strategies were used to complement these efforts. In order to attract nursing staff to the online content, they were actively involved in selected content formats, including podcasts and visual materials. Platform-derived user statistics from Facebook and Instagram suggested that the content reached a predominantly young and female audience. However, these data are based on platform-specific analytics and should be interpreted as indirect indicators only. While the observed age and gender distribution (e.g., 25–34 years on Instagram and 35–44 years on Facebook) may be compatible with certain healthcare workforce demographics, no direct conclusions about professional background can be drawn. In particular, it cannot be confirmed whether specific groups such as nursing staff were reached. Thus, these activities should be understood as efforts to increase visibility across potential target groups rather than as evidence of successful targeting of specific professional groups. Facebook is still the most popular social network. In the ranking of the largest social networks and messengers by number of users, Facebook is in first place with around 3.05 billion monthly active users worldwide. According to a Statista survey among regular social media users, Facebook is the most popular social network in Germany. Seventy-two percent of respondents stated that they use Facebook regularly, followed closely by YouTube with 71 percent. Instagram ranks third, with 56 percent of respondents reporting regular use (Statista, https://de.statista.com).The LinkedIn network has also established itself in the professional field in recent years; the network provides various professional data about its users (https://de.statista.com). The data on LinkedIn users shows that they represent a different sample. In terms of their age group, LinkedIn users were comparable to Facebook users: the majority of them belonged to the millennial generation, with the majority of LinkedIn users being male. On LinkedIn, the PRiVENT project was mainly followed by healthcare professionals in leadership positions. We suspect that these were mainly doctors, respiratory therapists and nursing managers from the various intensive care units, as well as administrative staff from the hospitals and employees from the medical industry.

The way consumers get their health information has changed. With the increasing importance of the internet as a source of information, there is also an increasing risk of misinformation being spread. A systematic review of the nature and potential causes of health-related misinformation showed an overall upward trend in the number of articles published on health-related misinformation and the role of social media in its dissemination [16]. There is a growing awareness in the medical community of the relevance of information on social media, which our patients often already use as their only source of information in addition to direct dialogue with their doctor. Objective patient information is seen as necessary to avoid misinformation in medically sensitive areas such as weaning from mechanical ventilation and HMV.

### Limitations

This study has several limitations. First, due to the heterogeneous data sources and the lack of occupational identifiers, we were only able to obtain an indirect impression of the users of our content, which limits conclusions about whether the intended target groups were reached. In addition, platform-specific metrics (e.g., views, reach, engagement) are based on heterogeneous and partly non-transparent definitions, which restricts comparability across platforms and limits the validity of cross-platform or distributional analyses. Second, the descriptive nature of our analysis and the absence of inferential or comparative statistical approaches restricts the overall scholarly contribution, as similar social-media evaluations are common in health communication research. At the same time, this study— to the best of our knowledge—represents the first structured, multi-platform digital communication approach in the field of long-term mechanical ventilation in Germany and should therefore be understood as an exploratory, hypothesis-generating analysis. While descriptive temporal trends are presented, more advanced comparative analyses (e.g., across platforms or content types) remain methodologically challenging due to the lack of standardized metrics and a consistent baseline. Third, our study design did not allow for a deeper examination of structural factors such as algorithmic bias, sustainability of engagement, echo-chamber dynamics, or cost-effectiveness. In particular, content visibility and reach are influenced by platform-specific and largely non-transparent algorithms, which may introduce bias and cannot be accounted for in the present analysis. Furthermore, our evaluation is based predominantly on organic reach, which may further limit generalizability. Although temporal trends are presented (Fig 2), the sustainability of engagement over time remains difficult to assess, especially in the absence of a defined post-campaign phase due to ongoing platform activity. In addition, potential echo-chamber effects cannot be excluded, as health-related social media communication may preferentially reach already engaged or interested audiences rather than broader populations. A formal assessment of cost-effectiveness was beyond the scope of this analysis; however, such evaluation is planned within the broader PRiVENT project and represents an important area for future research. Furthermore, temporal analyses are limited by the absence of a defined pre-intervention baseline and a clearly delineated post-intervention phase, and benchmarking against comparable initiatives is not feasible, as no similar structured programs or reference data currently exist in Germany. Finally, the specificity of the campaign content, aimed at a small and heterogeneous group of patients, relatives, and healthcare professionals, further limits the generalisability of our findings. Despite these limitations, the study offers empirical insights into the feasibility and reach of a collaborative digital health communication approach within the PRiVENT project. However, knowledge translation in this domain is complex and typically requires close collaboration between scientific and communication experts [17]. The PRiVENT project provided an opportunity to explore such collaboration and to analyse its outputs; nevertheless, the broader responsibility for structured public communication ultimately lies with medical societies. Despite these limitations, our experience suggests that meaningful content can be produced for specialised topics and can reach distinct user groups—including, unexpectedly, a substantial number of younger users.

## Conclusion

Our aims were to provide accessible information on long-term invasive ventilation and weaning, to highlight the situation of patients requiring out-of-hospital intensive care, and to increase public and professional visibility of specialised weaning centres. In addition, we sought to support the dissemination of weaning expertise among ICU staff. Our analysis indicates that social media can serve as an effective channel to communicate such content, with different platforms reaching distinct target groups. Beyond documenting communication metrics, our findings provide practical insights into the implementation of digital communication strategies in highly specialised healthcare settings. The observed differences in reach and engagement across platforms suggest that a coordinated multi-channel approach may be necessary to effectively address the diverse information needs of patients, relatives, healthcare professionals, researchers, and the broader public. These observations contribute to the growing literature on digital health communication by illustrating how different communication channels can complement each other when disseminating complex healthcare topics and engaging diverse stakeholder groups in a real-world setting.
