# Supplementary material for: Social media activities to enhance the visibility of long-term invasive ventilation: A content and visibility analysis from the PRiVENT study
Source: PLoS One. 2026 Jun 25;21(6):e0352267. doi: 10.1371/journal.pone.0352267 (PMC13298930; doi:10.1371/journal.pone.0352267)
Supplement: Attachment — Submitted filename: PONE-D-25-24730_reviewer.pdf [file pone.0352267.s001.pdf]

PLOS ONE

The use of social media in PRiVENT to raise awareness of long-term invasive ventilation

--Manuscript Draft--

**Manuscript Number:**	PONE-D-25-24730
**Article Type:**	Research Article
**Full Title:**	The use of social media in PRiVENT to raise awareness of long-term invasive ventilation
**Short Title:**	Social media in PRiVENT
**Corresponding Author:**	Julia Dorothea Michels-Zetsche<br>Thoraxklinik-Heidelberg gGmbH<br>Heidelberg, GERMANY
**Keywords:**	Social Media; long-term ventilation; weaning from mechanical ventilation; social perception
**Abstract:**	Background<br><br>Despite the increasing number of patients receiving long-term invasive mechanical ventilation (IMV) and the social importance of this issue, it is largely neglected in the public perception. One aim of the PRIVENT project was to raise public awareness of the issue.<br><br>Methods<br><br>A team of physicians, health researchers, an art director and a scientific editor developed an information campaign, including blog posts, podcasts, social media posts and information for medical professionals or patients/their relatives and made them available to the public on a dedicated homepage and various social media platforms. The number of views was used to measure the reach of the campaign.<br><br>Results<br><br>In the period from 02/2021–03/2025, a total of 72 blog posts and 880 social media posts and 3 podcast seasons with 21 episodes were made available to the public. The total reach was 2.6 million, with Facebook, Instagram and LinkedIn being the most used platforms with a total of 1872 followers. The most popular blog posts were those that explained the basics of IMV in plain language. Followers of the different social networks differ. Instagram users are younger and more likely to be female than those on Facebook and LinkedIn.<br><br>Conclusion<br><br>The results show that the use of social media can be one way to raise awareness for long-term IMV among a wide audience. On an issue of such importance, the public should be involved to empower those affected and to highlight the importance of weaning in the medical community and in society's perception.<br><br>Trial registration number<br><br>The PRiVENT study is registered at ClinicalTrials.gov (NCT05260853) on 02/03/2022.
**Order of Authors:**	Franziska C. Trudzinski
	Nicola Litke
	Olaf Buntemeyer
	Simone Janssen
	Elena Biehler

Powered by Editorial Manager® and ProduXion Manager® from Aries Systems Corporation

	Thomas Fleischhauer
	Jana C. Dahlhoff
	Philipp Höger
	Axel Kempa
	Benjamin Neetz
	Biljana Joves
	Claus Neurohr
	Alessandro Ghiani
	Armin Schneider
	Joachim Szecsenyi
	Jan Meis
	Janina Schubert-Haack
	Felix JF Herth
	Julia Dorothea Michels-Zetsche
	the PRiVENT-Study Group
**Additional Information:**	
**Question**	**Response**
**Financial Disclosure**<br><br>Enter a financial disclosure statement that describes the sources of funding for the work included in this submission. Review the submission guidelines for detailed requirements. View published research articles from *PLOS ONE* for specific examples.<br><br>This statement is required for submission and **will appear in the published article** if the submission is accepted. Please make sure it is accurate.<br><br>**Funded studies**<br>Enter a statement with the following details:<br>• Initials of the authors who received each award<br>• Grant numbers awarded to each author<br>• The full name of each funder<br>• URL of each funder website<br>• Did the sponsors or funders play any role in the study design, data collection and analysis, decision to publish, or preparation of the manuscript?	Yes

Did you receive funding for this work?	
Please add funding details.<br>as follow-up to "**Financial Disclosure**<br><br>Enter a financial disclosure statement that describes the sources of funding for the work included in this submission. Review the submission guidelines for detailed requirements. View published research articles from *PLOS ONE* for specific examples.<br><br>This statement is required for submission and **will appear in the published article** if the submission is accepted. Please make sure it is accurate.<br><br>**Funded studies**<br>Enter a statement with the following details:<br>• Initials of the authors who received each award<br>• Grant numbers awarded to each author<br>• The full name of each funder<br>• URL of each funder website<br>• Did the sponsors or funders play any role in the study design, data collection and analysis, decision to publish, or preparation of the manuscript?<br><br>Did you receive funding for this work?"	Funding by the Innovation Fund of the Federal Joint Committee (G-BA), grant number: 01NVF19023.
Please select the country of your main research funder (please select carefully as in some cases this is used in fee calculation).<br>as follow-up to "**Financial Disclosure**<br><br>Enter a financial disclosure statement that describes the sources of funding for the work included in this submission. Review the submission guidelines for detailed requirements. View published research articles from *PLOS ONE* for specific examples.<br><br>This statement is required for submission and **will appear in the published article** if the submission is accepted. Please make	GERMANY - DE

Powered by Editorial Manager® and ProduXion Manager® from Aries Systems Corporation

sure it is accurate.<br><br>**Funded studies**<br>Enter a statement with the following details:<br>• Initials of the authors who received each award<br>• Grant numbers awarded to each author<br>• The full name of each funder<br>• URL of each funder website<br>• Did the sponsors or funders play any role in the study design, data collection and analysis, decision to publish, or preparation of the manuscript?<br><br>Did you receive funding for this work?"	
**Competing Interests**<br><br>Use the instructions below to enter a competing interest statement for this submission. On behalf of all authors, disclose any competing interests that could be perceived to bias this work—acknowledging all financial support and any other relevant financial or non-financial competing interests.<br><br>This statement is required for submission and **will appear in the published article** if the submission is accepted. Please make sure it is accurate and that any funding sources listed in your Funding Information later in the submission form are also declared in your Financial Disclosure statement.<br><br>View published research articles from *PLOS ONE* for specific examples.	FCT reports payment or honoraria for lectures, or reimbursement of travel expenses from Novartis AG, GlaxoSmithKline, Chiesi, Boehringer Ingelheim GmbH, Grifols and AstraZeneca. JDMZ payment or honoraria for lectures, or reimbursement of travel expenses from CSL Behring and AstraZeneca. All authors declare funding by the Innovation Fund of the Federal Joint Committee (G-BA), grant number: 01NVF19023.

**NO authors have competing interests**<br><br>Enter: *The authors have declared that no competing interests exist.*<br><br>**Authors with competing interests**<br><br>Enter competing interest details beginning with this statement:<br><br>*I have read the journal's policy and the authors of this manuscript have the following competing interests: [insert competing interests here]*<br><br>* typeset	
**Ethics Statement**<br><br>Enter an ethics statement for this submission. This statement is required if the study involved:<br><br>• Human participants<br>• Human specimens or tissue<br>• Vertebrate animals or cephalopods<br>• Vertebrate embryos or tissues<br>• Field research<br><br>Write "N/A" if the submission does not require an ethics statement.<br><br>General guidance is provided below. Consult the submission guidelines for detailed instructions. **Make sure that all information entered here is included in the Methods section of the manuscript.**	This is a retrospective analysis of user data from an online platform, which were only available in fully anonymised form. No personal data were collected during the course of the study, and the data were processed in such a way that no individual could be identified. As a result, obtaining informed consent from participants was not possible and was not required.<br>The study protocol version 1.0 was first approved by the ethics committee of the Medical Faculty Heidelberg prior to the start of the study (S-352/2018) on 18th September 2020, version 1.4 was approved on 27th April 2021.

**Format for specific study types**<br><br>**Human Subject Research (involving human participants and/or tissue)**<br>• Give the name of the institutional review board or ethics committee that approved the study<br>• Include the approval number and/or a statement indicating approval of this research<br>• Indicate the form of consent obtained (written/oral) or the reason that consent was not obtained (e.g. the data were analyzed anonymously)<br><br>**Animal Research (involving vertebrate animals, embryos or tissues)**<br>• Provide the name of the Institutional Animal Care and Use Committee (IACUC) or other relevant ethics board that reviewed the study protocol, and indicate whether they approved this research or granted a formal waiver of ethical approval<br>• Include an approval number if one was obtained<br>• If the study involved *non-human primates*, add *additional details* about animal welfare and steps taken to ameliorate suffering<br>• If anesthesia, euthanasia, or any kind of animal sacrifice is part of the study, include briefly which substances and/or methods were applied<br><br>**Field Research**<br><br>Include the following details if this study involves the collection of plant, animal, or other materials from a natural setting:<br>• Field permit number<br>• Name of the institution or relevant body that granted permission	
**Data Availability**<br><br>Authors are required to make all data underlying the findings described fully available, without restriction, and from the time of publication. PLOS allows rare exceptions to address legal and ethical concerns. See the PLOS Data Policy and FAQ for detailed information.	No - some restrictions will apply

A Data Availability Statement describing where the data can be found is required at submission. Your answers to this question constitute the Data Availability Statement and **will be published in the article**, if accepted.<br><br>**Important:** Stating 'data available on request from the author' is not sufficient. If your data are only available upon request, select 'No' for the first question and explain your exceptional situation in the text box.<br><br>Do the authors confirm that all data underlying the findings described in their manuscript are fully available without restriction?	
**Describe where the data may be found in full sentences. If you are copying our sample text, replace any instances of XXX with the appropriate details.**<br><br>• If the data are **held or will be held in a public repository**, include URLs, accession numbers or DOIs. If this information will only be available after acceptance, indicate this by ticking the box below. For example: *All XXX files are available from the XXX database (accession number(s) XXX, XXX.)*.<br>• If the data are all contained **within the manuscript and/or Supporting Information files**, enter the following: *All relevant data are within the manuscript and its Supporting Information files.*<br>• If neither of these applies but you are able to provide **details of access elsewhere**, with or without limitations, please do so. For example:<br><br>*Data cannot be shared publicly because of [XXX]. Data are available from the XXX Institutional Data Access / Ethics Committee (contact via XXX) for researchers who meet the criteria for access to confidential data.*<br><br>*The data underlying the results presented in the study are available from (include the name of the third party*	Data are available from F. Trudzinski and Visuelle Werte

and contact information or URL).<br>• This text is appropriate if the data are owned by a third party and authors do not have permission to share the data.<br><br>* typeset	
Additional data availability information:	

Powered by Editorial Manager® and ProduXion Manager® from Aries Systems Corporation

Manuscript

Click here to access/download;Manuscript;Social media to raise public awareness of long-term IMV.docx

The use of social media in PRiVENT to raise awareness of long-term invasive ventilation

Franziska C. Trudzinski[1], Nicola Litke[2], Olaf Buntemeyer[3], Simone Janssen[3], Elena Biehler[2], Thomas Fleischhauer[2], Jana C. Dahlhoff[1], Philipp Höger[1], Axel Kempa[4], Benjamin Neetz[1], Biljana Joves[4], Claus Neurohr[5], Alessandro Ghiani[5], Armin Schneider[6], Joachim Szecsenyi[2,7], Jan Meis[8], Janina Schubert-Haack[7], Felix JF Herth[1], Julia D. Michels-Zetsche[1] and the PRiVENT-Study Group

Author affiliations:

[1]Thoraxklinik University Hospital Heidelberg, Department of Pneumology and Critical Care, Heidelberg, Translational Lung Research Centre Heidelberg (TLRC-H), Member of the German Centre for Lung Research (DZL), Heidelberg, Germany

[2]Department of General Practice and Health Services Research, University Hospital Heidelberg, Heidelberg, Germany

[3]Visuelle Werte GmbH, Berlin, Germany, www.visuelle-werte.de

[4]SLK-Klinik Löwenstein, Department of Pneumology and Critical Care, Löwenstein, Germany

[5]Robert-Bosch-Krankenhaus Klinik Schillerhöhe, Department of Pneumology and Respiratory Medicine, Gerlingen, Germany

[6]Department of Anesthesia and Intensive Care Medicine Waldburg-Zeil Kliniken, Wangen im Allgäu, Germany

[7]aQua Institute for Applied Quality Improvement and Research in Health Care, Göttingen, Germany

[8]Institute of Medical Biometry, Heidelberg University, Heidelberg, Germany

Correspondence to: Dr Julia Michels-Zetsche, E-mail: Julia.Michels@med.uni-heidelberg.de, https://orcid.org/0000-0002-0980-7586

Words: 3060, Abstract 263

Figures: 3, Tables:3

Key words: Social media, long-term ventilation, weaning from mechanical ventilation, social perception

Abstract

Background

Despite the increasing number of patients receiving long-term invasive mechanical ventilation (IMV) and the social importance of this issue, it is largely neglected in the public perception. One aim of the PRIVENT project was to raise public awareness of the issue.

Methods

A team of physicians, health researchers, an art director and a scientific editor developed an information campaign, including blog posts, podcasts, social media posts and information for medical professionals or patients/their relatives and made them available to the public on a dedicated homepage and various social media platforms. The number of views was used to measure the reach of the campaign.

Results

In the period from 02/2021–03/2025, a total of 72 blog posts and 880 social media posts and 3 podcast seasons with 21 episodes were made available to the public. The total reach was 2.6 million, with Facebook, Instagram and LinkedIn being the most used platforms with a total of 1872 followers. The most popular blog posts were those that explained the basics of IMV in plain language. Followers of the different social networks differ. Instagram users are younger and more likely to be female than those on Facebook and LinkedIn.

Conclusion

The results show that the use of social media can be one way to raise awareness for long-term IMV among a wide audience. On an issue of such importance, the public should be involved to empower those affected and to highlight the importance of weaning in the medical community and in society's perception.

Trial registration number: The PRiVENT study is registered at ClinicalTrials.gov (NCT05260853) on 02/03/2022.

Introduction

The increasing number of patients being discharged with invasive mechanical ventilation (IMV) is the downside of modern intensive care medicine in Germany (1). For these patients and their families, discharge to home mechanical ventilation (HMV) represents a radical change in their lives and is associated with a significant reduction in their independence. Patients on HMV are usually in need of round-the-clock intensive care. The existing shortage of qualified staff in intensive care medicine is well known, and recruiting well-trained staff to provide outpatient care will only worsen the problem. The resulting costs are a significant burden on the health care system and thus on the general public (2, 3). Nevertheless, this topic is hardly addressed in the public perception.

We suspect there are several reasons for this: The healthy do not usually want to deal with such an unpleasant subject because they assume that it will not affect them. Patients with chronic lung disease who are at risk of long-term ventilation, on the other hand, suppress the topic even more, as it is particularly painful and associated with great anxiety. In addition, those affected are often severely disabled and are unable to express their thoughts and feelings to the public due to their severe impairments, of which the tracheotomy is often only one aspect. HMV patients and their families often struggle to manage their daily lives and have few opportunities to participate in social life (4). In addition to the general public, the topic of long-term ventilation primarily concerns staff in intensive care units, who are frequently confronted with it but have considerable gaps in their knowledge about weaning from mechanical ventilation (5-7).

Social media is one way to reach these different groups and promote discourse on this complex issue. The German project PRiVENT is investigating innovative forms of care for invasively ventilated patients with the aim of reducing the number of patients discharged from hospital receiving invasive mechanical ventilation (8). One of the project's goals is to raise public awareness of the issue of long-term ventilation and to empower those affected and their families. This will be achieved by providing interesting and targeted information on various topics related to invasive ventilation and inpatient and out-of-hospital care for long-term ventilated patients via social media. The aim of this study is to analyse the impact of the project's social media work based on the social media reach on the platforms used.

Methods

Within the project PRiVENT, it was secondary aim to raise awareness for the topics weaning, invasive ventilation and inpatient and out-of-hospital care for long-term ventilated patients. To achieve this, a targeted social media campaign was designed and its impact was analysed within this study. The campaign and all content were published in German language.

The PRiVENT public relations team

The content of the social media campaign within the PRiVENT project was created by a multi-professional team consisting of a pulmonologist and intensive care physician, health researchers as well as advertising and knowledge communication experts, an art director and a scientific writer. All topics of the contributions were coordinated in a weekly virtual editorial conference in which the whole team took part.

Project homepage and social media platforms

The heart of the campaign is the project homepage "www.wieder-selbst-atmen.de”, which went online in February 2021 and provides access to almost all content and links to the other social media channels. Project accounts were opened for the social media platforms Facebook, Instagram, the video platform YouTube, the career network LinkedIn, and the community for doctors, medical assistants, pharmacists, and other healthcare professionals DocCheck.

Podcasts

The content of the podcasts was planned during the editorial meetings and aimed for 20 to 30 minutes per episode. In order to create thematically coherent podcast seasons, the topics were planned in advance for each entire season and inter-seasonal. Each episode was structured using a semi structured interview guideline. Lead questions were sent to the participants in advance. The requested guests received no honorarium for their participation. Whenever possible, the moderated podcast episodes were filmed on location with guests and the moderator at the Thoraxklinik in Heidelberg. In cases where the organisational effort for this was too great, the guests were connected online. The recorded interviews were then edited and reviewed by the entire team. Subtitling was required for one episode because the invited guest was difficult to understand due to his non-invasive ventilation. The finished

podcast episodes were promoted on social media platforms and made available through YouTube, Deezer, Google Podcasts, Apple Podcasts, and Spotify video and audio services.

Blog posts

In the editorial meetings, the topics of the blog posts were also agreed upon; these were written in plain language by a scientific editor and proofread by the collected team prior to publication. The contributions include various aspects of invasive mechanical ventilation, such as the tasks of the different professional groups in an intensive care unit or weaning unit, background information and resources on scientific publications and the published podcasts.

Social media posts

In addition, social media posts were published on a regular basis via Facebook, Instagram, YouTube, LinkedIn and DocCheck to highlight current developments in the project as well as blog posts and podcasts. In order to establish a lively network and integrate different professional groups involved in the weaning process, various PRiVENT project partners were introduced and team photos of staff from the participating ICUs were displayed. The contributions were actively promoted on Facebook between 04/2021 and 12/2024.

Target group characteristics

For professional advertisers, Facebook offers the opportunity to address specific target groups directly. The target group was selected on the basis of age 18-65 years and the various criteria listed below for the target groups of patients and relatives and healthcare professionals. 1.) Criteria for patients and relatives: Areas of interest: Quality of life (psychology), disease prevention, Industries: Health and social care, non-profit and social services. 2.) Criteria for healthcare professionals: Interests: geriatric nursing, Employee: nurse, Job title: nurse, geriatric nurse, nurse, exam. Elderly carer, Nursing service manager, Elderly carer, Nursing assistant, Elderly care assistant, Sector: Healthcare. Advertising was limited to the region of Baden-Württemberg in which the project took place.

Content reach

Content reach was assessed by the number of views, impressions and interactions provided by the platforms. In this context, general reach is equated with views, which determine how many people have seen the content. Impressions measure the number of times a piece of

content is viewed. This metric also considered repeat impressions or people who viewed the same content twice. The engagement measures the individual interactions (e.g. reactions, comments) with the content. The different social networks provide different information about their users. The available data of followers on Facebook, Instagram and LinkedIn was analysed to get more information about the users of PRiVENT content.

Statistical methods

The number of contents and the related views interactions were accessed 12/03/2025 and analysed descriptively, the overall content reach as well as the reach on the different platforms and contributions are given in absolute numbers and/or %.

Results

In the period from 02/2021–03/2025, a total of 72 blog posts, 880 social media posts and 3 podcast seasons with a total of 21 episodes were made available to the public.

Content of the contributions

The aim of the podcasts was to give a differentiated impression of the current situation of patients on long-term mechanical ventilation and to highlight it from different perspectives. For this purpose, invasively and non-invasively ventilated patients, their relatives, representatives of self-help groups, doctors, respiratory therapists, nursing staff and employees of the largest statutory health insurance in Germany, the AOK, had the opportunity to present on the podcasts. **Table 1** indicates the topics of all podcasts published to date (03/2025). The themes discussed ranged from the history of ventilation to the current situation in intensive care units during the COVID-19 pandemic. **Figure 1** shows impressions from the interviews. The blog posts related to the content of the podcasts and provided background information. They also covered general topics for people with respiratory diseases, such as information on topics like "Apps that help manage respiratory diseases", "How to motivate yourself to do breathing exercises?" and "Pulmonary rehabilitation: effective training for different pulmonary conditions". There was also specific information on acute intensive care, such as "Animals in intensive care: more than just a fluffy visit", "Children as relatives – when parents are in intensive care" or "Artificial intelligence in intensive care". Other topics included weaning from mechanical ventilation, such as "What does 'weaning' mean and how does it work?" or "Who works on a ventilator weaning unit?", and the care situations of patients with HMV, such as "Self-determined life with mechanical ventilation" and "Ventilation in children and the role of nursing", as well as topics relating to aftercare in the ICU and the sequelae of intensive care, such as "Post-COVID syndrome – the difference between recovered and healthy".

Season/Episode	Title	Access numbers
Season 1 #1	How the PRiVENT project aims to improve ventilator weaning	148
Season 1 #2	How PRiVENT will improve the situation of patients on mechanical ventilation	85

Season 1 #3	When do people need ventilation and what are the risks?	102
Season 1 #4	Special episode – How the Corona pandemic affects work in intensive care units	70
Season 1 #5	How can patient support organisations support people on mechanical ventilation?	73
Season 1 #6	The importance and effectiveness of self-help and comprehensive patient education	61
Season 1 #7	How do certified weaning centres work and what are the benefits for patients?	93
Season 2 #1	Update on the study	55
Season 2 #2	History of Mechanical Ventilation	78
Season 2 #3	chronic obstructive pulmonary disease	84
Season 2 #4	The role of the AOK Baden-Württemberg in PRiVENT	68
Season 2 #5	What do respiratory specialists do?	73
Season 2 #6	Post COVID-Syndrome – The difference between recovered and healthy	83
Season 3 #1	How intensive care units and weaning centres work together	66
Season 3 #2	Quality Circles in the Weaning Process	69
Season 3 #3	Invasive mechanical ventilation in children and the role of Nursing	67
Season 3 #4	Self-determined life with mechanical ventilation	59
Season 3 #5	A plea for more patience and mutual understanding	61
Season 3 #6	In a wheelchair at 18 and 30 years later	64
Season 3 #7	What's new in the PRiVENT project	40
Season 3 #8	An interview with Prof Dr Bernd Schönhofer	162

Table 1: Topics of the published podcasts

The blog posts provide information about the PRiVENT-project and illuminate the topic of "invasive long-term ventilation" from various perspectives. In the 20-minute videos, patients, hospital staff (pneumologists, intensive care physicians, and respiratory therapists), patient support groups, and health insurance companies describe their views on long-term invasive ventilation, thus providing an insight into the complex topic. Image excerpts from various interview situations are shown in Figure 2.

Content reach

The total reach was 2.6 million views with Facebook and Instagram being the most used platforms with 1.7 and 0.6 million views, respectively, and a total of 1018 followers. The third most popular platform was LinkedIn, where 854 people are currently following the content.

Figure 2 shows the evolution of total reach and provides an overview of the reach of the different platforms.

The top 10 most popular pages by number of hits

With 161462 views from 74751 visitors, the most popular online contributions to the project were those that explained the basics of weaning and mechanical ventilation in simple language. The most popular post was entitled "What is weaning and how does it work" (35617 views), followed by "What is invasive ventilation" (27236 views) and "Respiratory trainers in physiotherapy and respiratory therapy" (10438 views). **Table 2** shows the topics and access figures for the 10 most popular posts.

Most popular pages by number of visits	Type	Views
What does "weaning" mean and how does weaning work?	Blog post	35617
What is invasive mechanical ventilation?	Blog post	27236
Respiratory trainers in physiotherapy and respiratory therapy		10438
"What is a residential community for HMV patients and why have these facilities been criticised?"	Blog post	9583
Exercise to strengthen and stretch the lateral respiratory muscles	Blog post	7115
physical activity against breathlessness during exercise	Blog post	4268
Lungs and respiratory pump: the two parts of the respiratory system	Blog post	3175
What do respiratory therapists do?	Blog post	3162
The PRiVENT e-learning programme	Specialist information	1438
PRiVENT download material	Specialist information	1241

Table 2: The top 10 most popular blog posts (accessed 03/2025)

Users of the different platforms

Compared to Facebook, Instagram users were younger, with the majority aged between 25 and 30 years, compared to 35 to 44 years for Facebook. Instagram also had a higher proportion of female users, with 58 % of users being female compared to 47 % on Facebook (accessed 03/2025). The business network LinkedIn does not provide information about the sex and age of its users, but a review of user profiles shows that LinkedIn users were more likely to be men than on other platforms, with 38.3 % describing themselves as experienced professionals, 8.4 % as managers and 9.6 % as directors. Recent graduates account for only 25.5 % of the total, while interns and trainees account for 2.0 %. The majority of followers worked in the healthcare sector, with 42.2 % in healthcare, 9.5 % in business development and 8.3 % in research. Accordingly, 29.6 % of followers worked in hospitals and healthcare facilities, 12.4 % in medical practices and 10.5 % in the manufacture of medical devices. 5.6 % worked in pharmaceutical manufacturing and 4.9 % in colleges and universities (accessed (03/2025). **Figure 3** provides an overview of the age and gender distribution of Facebook and Instagram users, while **Table 3** provides information on users of the professional network LinkedIn.

**LinkedIn followers**	**Nr. (%) N = 856**
Area of professional activity	
Health services	361 (42.2)
Distribution and sales	81 (9.5)
Business development	71 (8.3)
Scientific research	55 (6.4)
in training and education	36 (4.2)
Human Resources	19 (2.2)
Others	233 (27.2)
Business sector	
Hospitals and healthcare facilities	253 (29.6)
Medical practices	106 (12.4)
Manufacture of medical devices	90 (10.5)
Hospitals	59 (6.9)
Pharmaceutical production	48 (5.6)
Universities and colleges	42 (4.9)

Others	258 (30.1)
Career level	
professionally experienced	328 (38.3)
young professionals	218 (25.5)
director	82 (9.6)
manager	72 (8.4)
vice president	31 (3.6)
Internship/Training	17 (2.0)
others	108 (12.6)

Table 3: LinkedIn followers (accessed 03/2025)

Discussion

The aim of the PRiVENT project was to inform the general public about various aspects of long-term mechanical ventilation. In the current analysis, the access numbers and user profiles of the social media activities within the project were studied. Between February 2021 and March 2025, a range of web-based content was created and made available to the public via a dedicated website and various social media platforms. In total, 72 blog posts, 880 social media posts and 3 podcast seasons with a total of 21 episodes were provided, highlighting the topic from different perspectives. The user information provided by the various platforms and the number of hits on the individual contributions indicate that different user groups were reached via the various channels and the wide range of information on offer. It should be noted that this is a very specialised topic that only appeals to a small number of users compared to lifestyle topics.

Analysis of the access showed that the most popular online posts were those explaining the basics of weaning and mechanical ventilation in plain language. Our article "What is weaning and how does it work?" was the most popular article with 35617 views and the second most popular article "What is invasive ventilation?" explained the basics of ventilation in layman's terms with a total of 27236 views. The fact that these articles were the most viewed suggests that the content of the campaign did reach the target group of patients and relatives. The access statistics give hint on the need for such information in Germany.

The involvement of patients and relatives, who are an important resource in the ICU recovery and weaning process (1, 9, 10), requires information and education. There is therefore a need for patients and relatives to understand intensive care therapies, for which they need objective and easily accessible sources of information, in addition to personal dialogue with the attending physicians. Another target group of the campaign was the intensive care staff of the various intensive care units involved. The main aim was to impart knowledge about weaning from mechanical ventilation. Nursing staff are a valuable resource in the weaning process as the active involvement of non-medical staff can shorten the duration of mechanical ventilation in ICU (11-13). As part of the project, various measures were taken to qualify the nursing staff for the topic of ventilator weaning (8). An analysis of the user statistics of the two largest social media platforms, Facebook and Instagram, revealed that they reached a predominantly young and female target group. In addition to bedside consultations in which

pneumologists and respiratory therapists from the weaning centres in the cooperating clinics discussed critical patient cases (14), an e-learning programme was offered for all professional groups involved (15). The effects of the weaning consultations were analysed in qualitative interviews, which revealed that nursing staff rarely had the opportunity to take part in these consultations due to staff shortages (16). In order to attract nursing staff to the online content, we therefore tried to actively involve them in the content. In addition, nursing staff from the hospital and non-clinical areas were given a voice in the podcasts, and photos of staff from the various wards who had agreed to take part were also shared on social media. On the one hand, this increased the reach, and on the other hand, this target group has been reached. An analysis of the user statistics of the two largest social media platforms, Facebook and Instagram, revealed that the content reached a predominantly young and female target group. On Instagram, the average age of followers was between 25 and 34 years, while the average age of Facebook users was slightly higher at 35 to 44 years. The age and gender distribution on Instagram in particular leads to the assumption that at least some of these followers were nurses.

Facebook is still the most popular social network. In the ranking of the largest social networks and messengers by number of users, Facebook is in first place with around 3.05 billion monthly active users worldwide. According to a Statista survey of regular social media users Facebook is the most popular social network in Germany. 72 % of respondents said they use this service regularly. 71 % of respondents regularly use YouTube and 56 % Instagram (https://de.statista.com).

The LinkedIn network has also established itself in the professional field in recent years; the network provides various professional data about its users (https://de.statista.com). The data on LinkedIn users shows that they represent a different sample. In terms of their age group, LinkedIn users were comparable to Facebook users: the majority of them belonged to the millennial generation, with the majority of LinkedIn users being male. On LinkedIn, the PRiVENT project was mainly followed by healthcare professionals in leadership positions. We suspect that these were mainly doctors, respiratory therapists and nursing managers from the various intensive care units, as well as administrative staff from the hospitals and employees from the medical industry.

The way consumers get their health information has changed. With the increasing importance of the internet as a source of information, there is also an increasing risk of misinformation being spread. A systematic review of the nature and potential causes of health-related misinformation showed an overall upward trend in the number of articles published on health-related misinformation and the role of social media in its dissemination (17). There is a growing awareness in the medical community of the relevance of information on social media, which our patients often already use as their only source of information in addition to direct dialogue with their doctor. Objective patient information is seen as necessary to avoid misinformation in medically sensitive areas such as weaning from mechanical ventilation and HMV.

Limitations

The main limitation of the study is that we were only able to indirectly obtain an impression of the users of our content due to the different types of information. The topics are generally very specific and are aimed at a certain, very small group of users. This is also reflected in the access figures for the blog posts, which appear low in comparison to the Livestyle posts. The translation of knowledge is complex and requires the co-operation of scientists and journalists alike (18). The PRiVENT project has opened up the opportunity to test this type of collaboration and to analyse the results in the context of a study. Ultimately, however, it is up to the medical societies to provide this type of information. The experience of this study was that the collaboration was fruitful, and the results show that we were able to create content that reached different groups of users. It was perceived as unexpected that it was achieved to reach so many young users with this topic.

Conclusion

Our aims were to raise public awareness of the issue of long-term ventilation and weaning from mechanical ventilation, and to provide information about the status of patients requiring out-of-hospital intensive care, as well as to show the patients, relatives and public that there are specialised weaning centres whose main role is to prevent long-term ventilation. We also aimed to further increase the weaning expertise of ICU staff. Our analysis shows that various social media represent a valid way to communicate such content, demonstrating that specific target-groups can be reached through different social networks.

Figures

Figure 1: The PRiVENT Podcast Series

The illustration shows various interview situations from the PRiVENT podcast. In addition to patients (A, D), respiratory therapists (B), respiratory physicians (C) and representatives of patient support organisations, a number of other stakeholders were interviewed to give an insight into the situation of long-term respiratory care. Wherever possible, the recordings were made in the library of the Tuberculosis Museum of the Thoraxklinik in Heidelberg. in cases where this would have been too inconvenient, video conferences were held with the podcast guests.

Figure 2: Content reach

The illustration shows A.) the development of reach over time and B.) the reach on the different platforms.

Figure 3: Distribution of age groups and gender stratified for Facebook and Instagram

The figure shows the gender distribution and the age groups of the users who follow the content on Facebook and Instagram.

Acknowledgements

The PRiVENT study group: Thoraxklinik Heidelberg: Felix Herth, Franziska Christina Trudzinski, Julia Dorothea Michels-Zetsche, Michael Müller, Benjamin Neetz, Gabriele Iberl, Jana Christina Dahlhoff, Philipp Höger; Universitätsklinikum Heidelberg Abteilung Allgemeinmedizin & Versorgungsforschung: Martina Bentner, Elena Biehler, Johanna Forstner, Gerhard Fuchs, Nicola Litke, Markus Qreini, Selina von Schumann, Noemi Sturm, Joachim Szecsenyi, Aline Weis, Michel Wensing; aQua-Institut für angewandte Qualitätsförderung und Forschung im Gesundheitswesen GmbH: Janina Schubert-Haack, Timm Frerk, Anja Klingenberg; SLK-Klinik Löwenstein: Axel Kempa, Biljana Joves, Andreas Rheinhold; Robert-Bosch-Krankenhaus Klinik Schillerhöhe: Claus Neurohr; Waldburg-Zeil Kliniken Fachkliniken Wangen: Armin Schneider, Christoph Rauch, Patrick Gehrig, Joachim Sugg, Susanne Hirschmann; Universitätsklinikum Heidelberg Medizinische Klinik 4 für Gastroenterologie, Infektionskrankheiten, Vergiftungen: Uta Merle; Universitätsklinikum Heidelberg Klinik für Anästhesiologie: Mascha Fiedler-Kalenka; SRH Kurpfalzkrankenhaus: Jens Regula; Siloah St. Trudpert Klinikum: Miriane Bomeken; Städtisches Klinikum Karlsruhe: Sebastian Stier; GRN-Klinik Schwetzingen: Jens Müller; Universitätsklinikum Mannheim Medizinische Klinik 1 Kardiologie, Angiologie, Hämostaseologie und Internistische Intensivmedizin: Simone Britsch; Universitätsklinikum Mannheim Klinik für Anästhesiologie und Operative Intensivmedizin: Jörg Krebs; Helios Klinikum Pforzheim: Ute Oltmanns; St. Josefskrankenhaus: Tom Terboven; SLK Klinikum am Gesundbrunnen: Marcus Hennersdorf; SLK Klinikum am Plattenwald: Neslihan Satir; Caritas-Krankenhaus Bad Mergentheim: Mathias Borst; Klinikum Heidenheim: Brigitte Mayer; Klinikum Crailsheim: Wolfgang Reikow; Ostalb Klinikum Aalen: Markus Kredel; St. Anna-Virngrund-Klinik Ellwangen: Konstantin Frey; Hohenloher Krankenhaus: Holger Wolff; Die Filderklinik: Florian Seidlitz; SRH Klinikum Karlsbad-Langensteinbach: Stefanie Bientzle; Zollernalb Klinikum: Boris Nohé; Robert-Bosch-Krankenhaus: Sebastian Allgäuer; Diakonie-Klinikum Stuttgart: Alexej Schöpp; RKU Ulm: Christoph Schlegel; Krankenhaus Landkreis Freudenstadt: Andrezj Kuzniar; Universitätsklinikum Tübingen Klinik für Anästhesie und Intensivmedizin: Helene Häberle; Universitätsklinikum Tübingen Medizinische Klinik: Reimer Riessen; Klinikum am Steinenberg: Benjamin Schempf; Klinikum Landkreis Tuttlingen: Ingo Rebenschütz; St. Elisabethen Klinikum Ravensburg: Andreas Straub; Hegau-Bodensee-Klinikum Singen 1 Medizinische Klinik Singen, Kardiologie und internistische Intensivmedizin: Marc Kollum; Alb-Donau-Kreis Krankenhaus Blaubeuren: Markus Winter; Alb-Donau-Kreis Krankenhaus Ehingen: Andreas Junginger; Oberschwabenklinik Westallgäu-Klinikum in Wangen: Stefan Locher; Universitätsklinikum Ulm: Peter Hartveg; Klinikum Friedrichshafen, Klinik für Anästhesiologie und Intensivmedizin: Mathias Vogel

Funding: This study is fully funded by the Innovation Fund of the Federal Joint Committee (G-BA), grant number: 01NVF19023.

Trial registration number: The PRiVENT study is registered at ClinicalTrials.gov (NCT05260853).

Competing interests: FCT reports payment or honoraria for lectures, or reimbursement of travel expenses from Novartis AG, GlaxoSmithKline, Chiesi, Boehringer Ingelheim GmbH, Grifols and AstraZeneca. JDMZ payment or honoraria for lectures, or reimbursement of travel expenses from CSL Behring and AstraZeneca. All authors declare funding by the Innovation Fund of the Federal Joint Committee (G-BA), grant number: 01NVF19023.

Patient consent for publication: This is a retrospective analysis of user data from an online platform, which were only available in fully anonymised form. No personal data were collected during the course of the study, and the data were processed in such a way that no individual could be identified. As a result, obtaining informed consent from participants was not possible and was not required.

Availability of data and materials: The datasets used and analysed during the current study are available from the corresponding author on reasonable request

Ethics approval: The study protocol version 1.0 was first approved by the ethics committee of the Medical Faculty Heidelberg prior to the start of the study (S-352/2018) on 18th September 2020, version 1.4 was approved on 27th April 2021.

Author Contributions Statement: FCT, NL, OB, SJ, EB, TF and JD jointly created the online content, helped interpret the data and proofread the manuscript. FCT and JDMZ drafted the first version of the manuscript. PH, AK, BN, BJ, CN, AG, AS, JS, JM, JS-H, FJF H provided input for the online content and proofread the manuscript. All authors made a substantial contribution and read and approved the final manuscript.

Literature

1. King AC. Long-term home mechanical ventilation in the United States. Respir Care. 2012;57(6):921-30; discussion 30-2.
2. Nonoyama ML, McKim DA, Road J, Guerriere D, Coyte PC, Wasilewski M, et al. Healthcare utilisation and costs of home mechanical ventilation. Thorax. 2018.
3. Michels JD, Trudzinski FC, Bornitz F, Ewert R, Muller M, Trinkmann F, et al. Costs of Weaning Failure: A Prospective, Multicentre, Controlled, Non-Randomised, Interventional Study on Economic Implications for the German Health Care System. Respiration. 2023;102(9):813-20.
4. Vitacca M, Escarrabill J, Galavotti G, Vianello A, Prats E, Scala R, et al. Home mechanical ventilation patients: a retrospective survey to identify level of burden in real life. Monaldi Arch Chest Dis. 2007;67(3):142-7.
5. Hassen KA, Nemera MA, Aniley AW, Olani AB, Bedane SG. Knowledge Regarding Mechanical Ventilation and Practice of Ventilatory Care among Nurses Working in Intensive Care Units in Selected Governmental Hospitals in Addis Ababa, Ethiopia: A Descriptive Cross-Sectional Study. Crit Care Res Pract. 2023;2023:4977612.
6. Guilhermino MC, Inder KJ, Sundin D, Kuzmiuk L. Education of ICU nurses regarding invasive mechanical ventilation: findings from a cross-sectional survey. Aust Crit Care. 2014;27(3):126-32.
7. Goldsworthy S. Mechanical Ventilation Education and Transition of Critical Care Nurses into Practice. Crit Care Nurs Clin North Am. 2016;28(4):399-412.
8. Michels JD, Meis J, Sturm N, Bornitz F, von Schumann S, Weis A, et al. Prevention of invasive ventilation (PRiVENT)-a prospective, mixed-methods interventional, multicentre study with a parallel comparison group: study protocol. BMC Health Serv Res. 2023;23(1):305.
9. Burns KEA, Misak C, Herridge M, Meade MO, Oczkowski S, Patient, et al. Patient and Family Engagement in the ICU. Untapped Opportunities and Underrecognized Challenges. Am J Respir Crit Care Med. 2018;198(3):310-9.

10. Palmqvist S. [Partial crowns as bridge abutments. Esthetics and retention]. Tandlakartidningen. 1980;72(8):459-63.
11. Sepahyar M, Molavynejad S, Adineh M, Savaie M, Maraghi E. The Effect of Nursing Interventions Based on Burns Wean Assessment Program on Successful Weaning from Mechanical Ventilation: A Randomized Controlled Clinical Trial. Iran J Nurs Midwifery Res. 2021;26(1):34-41.
12. Kimura R, Barroga E, Hayashi N. Effects of Mechanical Ventilator Weaning Education on ICU Nurses and Patient Outcomes: A Scoping Review. J Contin Educ Nurs. 2023;54(4):185-92.
13. Danckers M, Grosu H, Jean R, Cruz RB, Fidellaga A, Han Q, et al. Nurse-driven, protocol-directed weaning from mechanical ventilation improves clinical outcomes and is well accepted by intensive care unit physicians. J Crit Care. 2013;28(4):433-41.
14. von Schumann S, Ullrich C, Weis A, Wensing M, Litke N. Interprofessional weaning boards for invasively ventilated patients in intensive care units: Qualitative interview study with healthcare professionals in Germany. Res Nurs Health. 2023;46(1):148-58.
15. Michels-Zetsche JD, Schubert-Haack J, Tanck K, Neetz B, Iberl G, Muller M, et al. E-learning-an interventional element of the PRiVENT project to improve weaning expertise. BMC Med Educ. 2024;24(1):420.
16. Keller S, Forstner J, Weis A, Biehler E, Trudzinski F, Michels J, et al. [Interprofessional weaning boards and weaning consults for long-term ventilated patients: A qualitative study of perceived potential for patient care]. Pneumologie. 2024;78(3):180-90.
17. Wang Y, McKee M, Torbica A, Stuckler D. Systematic Literature Review on the Spread of Health-related Misinformation on Social Media. Soc Sci Med. 2019;240:112552.
18. Shapiro M. Media and education of the consumer. Menopause. 2016;23(8):924-7.

Click here to access/download;Figure;Figure1_1st.png

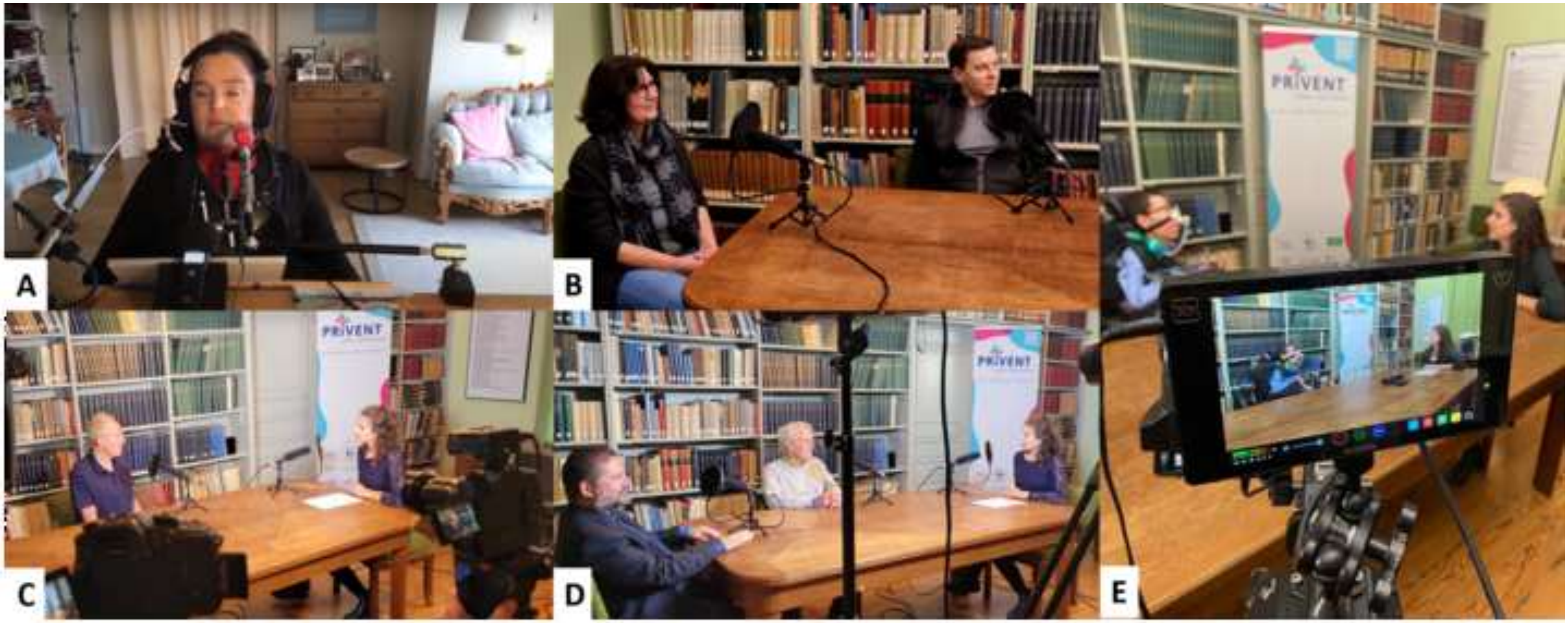

Figure

Click here to access/download;Figure;Figure 2.pdf

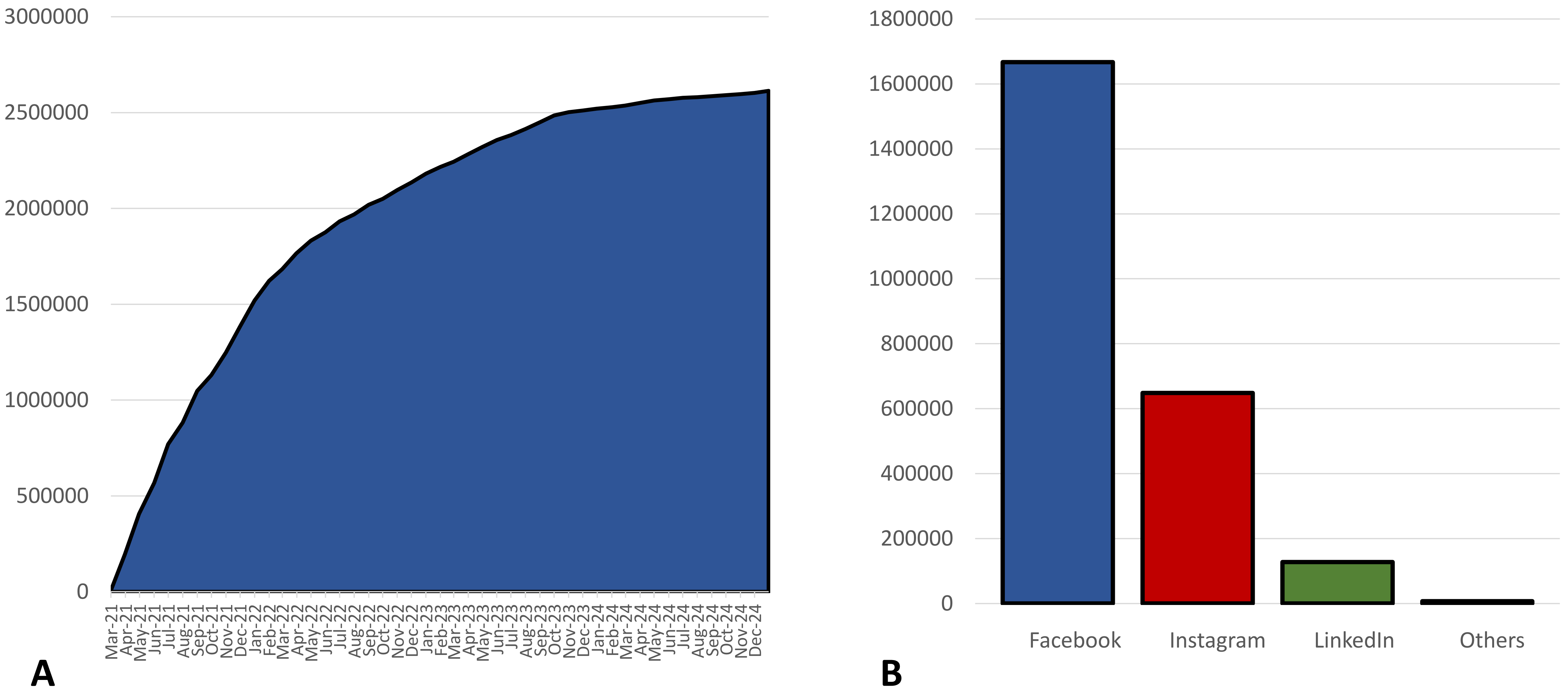

Click here to access/download;Figure;Figure 3.pdf

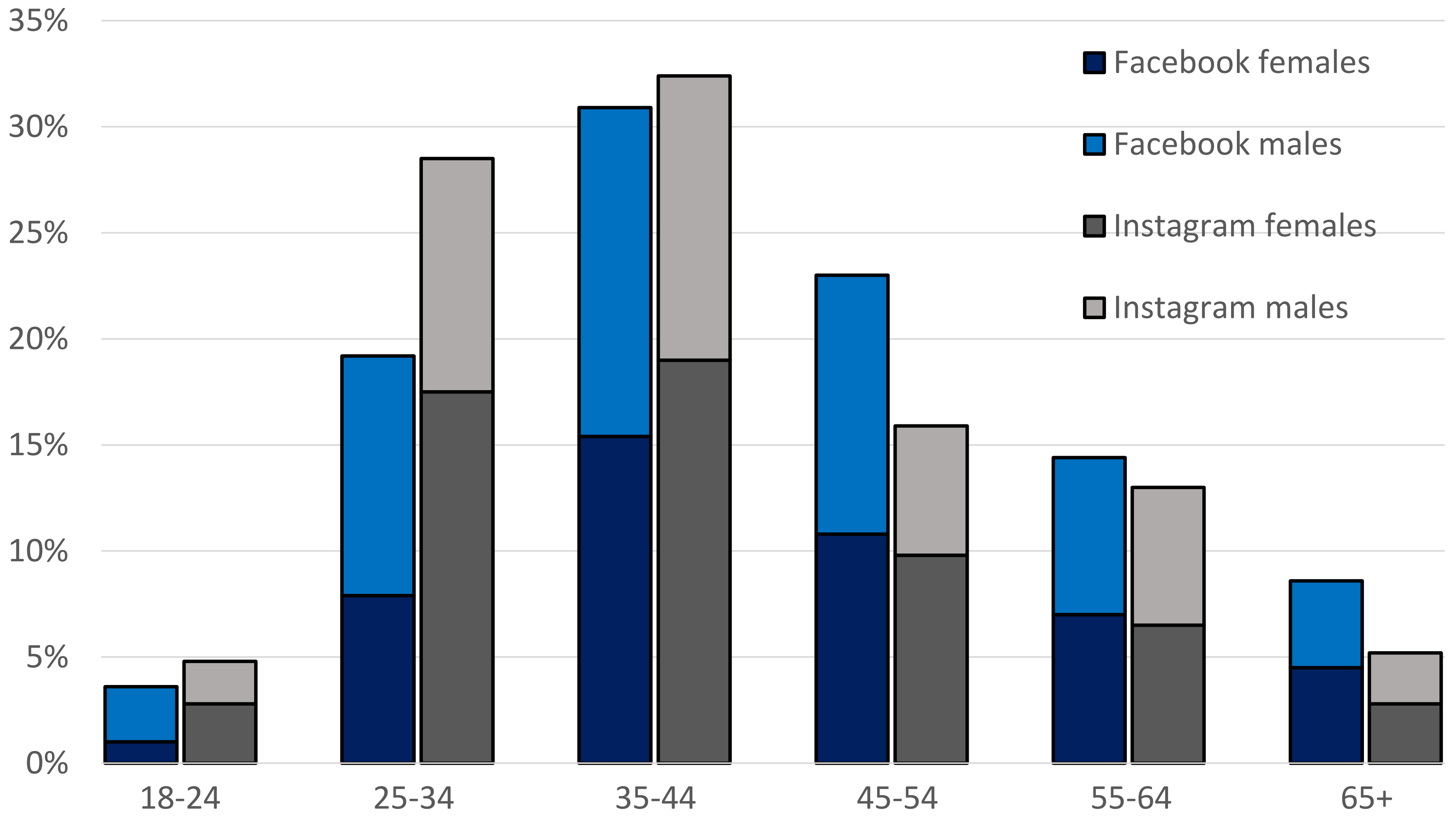

Click here to access/download
Supporting Information
STROBE_The use of social media.doc
