# Supplementary material for: Social media activities to enhance the visibility of long-term invasive ventilation: A content and visibility analysis from the PRiVENT study
Source: PLoS One. 2026 Jun 25;21(6):e0352267. doi: 10.1371/journal.pone.0352267 (PMC13298930; doi:10.1371/journal.pone.0352267)
Supplement: Attachment — Submitted filename: Response to Reviewers.docx [file pone.0352267.s004.docx]

***Response to Reviewers***

We would like to thank the Academic Editor and the reviewers for their thorough and constructive feedback on our manuscript “The use of social media in PRiVENT to raise awareness of long-term invasive ventilation.” We carefully considered all comments and have revised the manuscript accordingly. The responses below address each point raised by the reviewers in detail. All changes made to the manuscript are highlighted in the revised version.

**Reviewers’ comments:**

Reviewers’ comments:

**Reviewer #1:** The Privent project's use of social media to spread knowledge about long-term invasive mechanical ventilation is examined in this publication. The study presents a well-planned campaign that incorporates blogs, podcasts, and social media platforms, and it is timely and pertinent. Novelty, transparency, and public health significance are among its strong points. The use of descriptive statistics (views, impressions) in the absence of explicit indicators of empowerment or awareness, however, has drawbacks that need to be more explicitly recognized. Conclusions ought to be more circumspect, focusing on possible effects rather than validated results. Data accessibility might be enhanced. All things considered, this is a worthwhile addition that would be appropriate with only slight to moderate editing.

*We thank Reviewer #1 for the positive and constructive feedback. We appreciate the recognition of the study’s relevance, novelty, and public health significance. We acknowledge the limitation of relying on descriptive statistics without direct measures of empowerment or awareness and have revised the manuscript to discuss this more explicitly. The conclusions have been adjusted to adopt a more cautious tone, focusing on potential rather than confirmed effects. We also improved the section on data accessibility. We are grateful for the reviewer’s helpful comments, which have strengthened the manuscript.Thank you very much for this positive feedback and pointing out to stress the limitations of descriptive statistics, which we added in the discussion section. The conclusion was shortened.We hope this imprioved our manuscript.*

**Reviewer #2:** The manuscript tackles a significant and little-studied subject: using social media to increase awareness about long-term invasive mechanical ventilation. The approach is transparent about funding, ethics approval, and conflicting interests, and it is well explained. The findings are pertinent and show that various audiences were meaningfully engaged. The findings align with the information provided. There was no proof of publication misbehavior, ethical transgressions, or dual publication. By emphasizing the importance of digital communication in raising health awareness, the study provides a significant contribution. The manuscript would be strengthened even more with minor adjustments to the topic of generalizability and the clarity of the findings presentation.

*We thank Reviewer #2 for the positive and encouraging feedback and for acknowledging the study’s transparency, methodological rigor, and relevance. We appreciate the suggestion to further address the generalizability of our findings and have added a short discussion on this aspect.*

**Reviewer #3:** The manuscript addresses an important and timely issue: raising public awareness of long-term invasive mechanical ventilation (IMV) through the PRiVENT social media campaign. The study is well-structured, the objectives are clear, and the descriptive analysis provides valuable insight into how social media can serve as a platform for disseminating health-related information. I commend the authors for including a multidisciplinary team and for highlighting the role of patients, relatives, and healthcare professionals in shaping the campaign.

Strengths:The study is innovative in its focus on social media as a tool to engage both the general public and healthcare professionals in a sensitive medical topic. The dataset is substantial, covering a four-year period with diverse outputs (blog posts, podcasts, and social media posts).Results are clearly presented with supporting figures and tables, which help illustrate reach and demographic differences across platforms.Ethical considerations were well-handled, and the data were anonymized appropriately.

*We thank Reviewer #3 for the positive and thoughtful feedback and for recognizing the importance, structure, and clarity of our study. We greatly appreciate the acknowledgment of our multidisciplinary approach and the inclusion of patients, relatives, and healthcare professionals in shaping the PRiVENT campaign. We are pleased that the reviewer found the presentation of results, ethical considerations, and use of diverse communication formats to be clear and appropriate.We are grateful for these encouraging comments, which support the relevance and value of our work.*

Points for Improvement:Data Availability: The manuscript states that datasets are available upon request. PLOS ONE requires full data availability unless there are clear legal or ethical restrictions. Please clarify why full public access is restricted and consider providing at least anonymized aggregated data in a repository.

*We thank the reviewer for this valuable comment. The underlying data are derived from user interactions on social media platforms, which are subject to the platforms’ terms of use and data protection regulations. These legal and ethical restrictions prevent the public sharing of raw datasets, as user-level data cannot be fully anonymised without breaching platform policies.To ensure transparency, we have clarified this point in the Data Availability Statement. Summary data supporting the findings of this study can be requested from the corresponding author.*

Statistical Rigor: While descriptive statistics are appropriate for measuring reach and impressions, the analysis could be enhanced by including trend analyses (e.g., growth over time, engagement per type of content) or comparisons between platforms using simple statistical tests. This would strengthen the conclusions.

*We thank the reviewer for this valuable comment. The statistical approach was intentionally kept simple, as the primary aim of the study was to provide a descriptive overview of the campaign’s reach and engagement rather than to test specific hypotheses. Nevertheless, we acknowledge that more detailed analyses (e.g., trend or comparative analyses) could provide additional insights. We have therefore included this point as a limitation in the revised Discussion section and noted that future research could build on this by applying more advanced statistical methods*

Impact Assessment: The manuscript focuses primarily on quantitative reach. It would be beneficial to discuss qualitative outcomes, such as evidence of increased awareness, behavioral change, or stakeholder engagement beyond clicks and views.

*We thank the reviewer for this important suggestion. We agree that assessing qualitative outcomes such as increased awareness, behavioral change, or stakeholder engagement would provide valuable additional insights beyond quantitative reach. This limitation has been added to the revised Discussion section, where we note that future studies should incorporate qualitative or mixed-methods approaches to better evaluate the broader impact of social media-based communication efforts.*

We thank the reviewer for this insightful comment. Unfortunately, the available data do not allow for a qualitative assessment of increased awareness, behavioral change, or stakeholder engagement beyond quantitative metrics such as reach and impressions.

*We thank the reviewer for this important suggestion. We agree that assessing qualitative outcomes such as increased awareness, behavioral change, or stakeholder engagement would provide valuable additional insights beyond quantitative reach. This limitation has been added to the revised Discussion section, where we note that future studies should incorporate qualitative or mixed-methods approaches to better evaluate the broader impact of social media-based communication efforts.*

Literature Context: The discussion could be enriched by integrating more recent references (within the past 5 years) on health communication via social media, to situate the study more firmly within current discourse.

We thank the reviewer for this helpful suggestion. We have revised the Discussion section to include several more recent references (published within the past five years) on health communication via social media. These additions help to better position our study within the current research discourse.

*We thank the reviewer for this helpful suggestion. We have revised the Discussion section to include several more recent references (published within the past five years) on health communication via social media. These additions help to better position our study within the current research discourse. The corresponding changes have been highlighted in the revised manuscript.*

Language and Style: The manuscript is overall well-written, but some minor typographical and grammatical errors should be revised at copyediting (e.g., occasional redundancies in describing the platforms and content types). Data Availability: Statistical Rigor, Impact Assessment, Literature Context, Language and Style

*We thank the reviewer for the careful reading and constructive feedback. Minor typographical and grammatical issues have been corrected throughout the manuscript. We appreciate the note that additional adjustments can be addressed during copyediting.*

Overall Assessment:

This manuscript makes a useful contribution to the field of health communication and respiratory medicine. With improvements in data transparency, statistical depth, and contextual discussion, it will provide even stronger evidence for the role of social media in addressing underrepresented healthcare topics.

*We thank the reviewer for the careful reading and constructive feedback. We have carefully revised the manuscript to address the mentioned points. We appreciate the reviewer’s helpful comments, which have contributed to improving the overall clarity and quality of the manuscript.*

**Reviewer #4:** This manuscript addresses an important and underexplored issue: raising public awareness of long-term invasive mechanical ventilation through social media. The study is relevant, timely, and well-presented, with a clear description of campaign activities and transparent reporting of reach metrics. Its interdisciplinary approach and innovative use of multiple media formats are commendable. However, the manuscript focuses primarily on quantitative reach without sufficiently addressing qualitative impact, such as changes in knowledge, attitudes, or behaviors among patients, relatives, or healthcare staff. The Methods section is overly descriptive in parts, while the Discussion could be strengthened by deeper analysis, including comparison with similar health communication campaigns and exploration of broader policy implications.Overall, the manuscript is valuable but requires minor revision to improve its scientific contribution and generalizability.

Answer:

*We thank Reviewer #4 for the positive and constructive feedback. We appreciate the recognition of the study’s relevance, interdisciplinary approach, and transparent reporting of campaign activities. We have addressed the points raised in the revised manuscript: the limitations regarding qualitative impact, generalizability, and the descriptive nature of the Methods section are now explicitly discussed. The Discussion has also been strengthened with additional context and references to similar health communication campaigns, highlighting broader implications. We are grateful for the reviewer’s suggestions, which have helped improve the scientific contribution and clarity of the manuscript.Thank you very much for pointing out the value of our manuscript and the limitations with room for improvement. The topic of generalizability was also adressed by reviewer 2 and included in the conclusion. The issue of qualitative impact was raised by reviewer 3 as well.*
