# Supplementary material for: Social media activities to enhance the visibility of long-term invasive ventilation: A content and visibility analysis from the PRiVENT study
Source: PLoS One. 2026 Jun 25;21(6):e0352267. doi: 10.1371/journal.pone.0352267 (PMC13298930; doi:10.1371/journal.pone.0352267)
Supplement: Attachment — Submitted filename: Plos one accept.docx [file pone.0352267.s003.docx]

The authors have successfully addressed all the concerns raised during the previous review round. The revisions made to the Discussion and Limitations sections provide a more balanced view of the study’s findings, particularly regarding the descriptive nature of the data. I recommend the manuscript for publication in its current form. As a final constructive note, I encourage the authors to consider incorporating qualitative feedback mechanisms in future iterations of the project to better assess the educational impact on the target audience.
