# Supplementary material for: Social media activities to enhance the visibility of long-term invasive ventilation: A content and visibility analysis from the PRiVENT study
Source: PLoS One. 2026 Jun 25;21(6):e0352267. doi: 10.1371/journal.pone.0352267 (PMC13298930; doi:10.1371/journal.pone.0352267)
Supplement: Attachment — Submitted filename: Response_to_Reviewers_auresp_2.docx [file pone.0352267.s005.docx]

**Response to Reviewers’ Comments**

We thank the reviewers for their careful evaluation of our revised manuscript and for their constructive and positive feedback.

**Reviewer #1**

Reviewer #1 did not provide additional comments and indicated that all evaluation criteria have been satisfactorily met. We thank the reviewer for the positive assessment of our work.

**Reviewer #3**

We sincerely thank Reviewer #3 for the detailed review and for confirming that the major issues raised in the previous round have been adequately addressed. We respond to the remaining minor comments as follows:

**Comment 1: Minor language polishing is recommended, particularly in the Introduction and Discussion sections.**

**Response:**
The Introduction and Discussion have been carefully revised to improve clarity, flow, and readability. Minor grammatical and stylistic issues have been corrected throughout the manuscript.

**Comment 2: Please ensure consistency in terminology and formatting throughout the manuscript.**

**Response:**
The manuscript has been reviewed in its entirety to ensure consistent terminology, abbreviations, and formatting across all sections.

**Comment 3: Adding brief justifications for selected analytical parameters would improve methodological transparency.**

**Response:**
Brief justifications for key analytical parameters have been added to the Methods section where appropriate, improving transparency and clarity of the analytical approach.

We believe that these minor revisions further strengthen the manuscript and address all remaining concerns. We thank the reviewer again for the constructive comments and for considering the manuscript suitable for publication.
