# Supplementary material for: Social media activities to enhance the visibility of long-term invasive ventilation: A content and visibility analysis from the PRiVENT study
Source: PLoS One. 2026 Jun 25;21(6):e0352267. doi: 10.1371/journal.pone.0352267 (PMC13298930; doi:10.1371/journal.pone.0352267)
Supplement: Attachment — Submitted filename: Point_by_point.docx [file pone.0352267.s006.docx]

**Subject:** Re: Manuscript PONE-D-25-24730R2 – Revision 3

Dear Sulemana Bankuoru Egala

Thank you very much for your detailed evaluation of our manuscript “The use of social media in PRiVENT to raise awareness of long-term invasive ventilation”.

We appreciate the constructive feedback and the opportunity to submit another revision. We acknowledge the critical concerns raised by Reviewer 5 regarding conceptual framing, theoretical rigor, methodological approaches, and the operationalization of targets. We will substantially revise the manuscript to address all points raised, with particular attention to strengthening the theoretical underpinnings, clarifying methodological procedures, and more explicitly demonstrating the contribution of our work. We also sincerely thank Reviewer #6, who had no further comments and recommended the manuscript for acceptance. We appreciate this positive assessment and are pleased that the practical relevance and social value of the work were recognized. We hope that the revised version meets all expectations and remains at your disposal for any further questions.

Thank you again for the opportunity to improve our manuscript. We look forward to continuing the review process.

Kind regards,

Franziska Trudzinski, MD

(on behalf of all co-authors)

Reviewer #5: Review of manuscript no. PONE-D-25-24730

1. Conceptual overclaim: The central problem is conceptual. The study claims to assess whether social media “raises awareness,” yet no awareness, knowledge, attitude, perception, or behavioral outcomes were measured. Only platform analytics (views, impressions, followers) were analyzed. Visibility cannot be equated with awareness or empowerment. The conclusions therefore overstate what the data support. The manuscript must either (a) narrow its claims to “digital reach and engagement,” or (b) include outcome measures demonstrating actual awareness impact.

***Response:*** *We thank the reviewer for this important conceptual clarification and fully agree with the concern. Platform analytics provide information on visibility and reach, but they cannot be used to infer “awareness,” attitudes, or behavioral outcomes.*

*Following this recommendation, we have fundamentally revised the conceptual framing of the manuscript:*

1. *Terminology revised throughout:
   We systematically replaced the term “raises awareness” with more accurate terms such as “visibility,” “digital reach,” and “engagement metrics.” No remaining statements suggest an awareness or empowerment effect.*
2. *Revised study title to reflect this conceptual correction:
   The manuscript now uses the title:
   “Social media activities to enhance the visibility of long-term invasive ventilation: A content and visibility analysis from the PRiVENT study.”
   This title accurately reflects the methodological scope and avoids overstating the impact.*
3. *Adjusted aims and conclusions:
   The aims, results, and conclusions now explicitly describe the study as an analysis of content and visibility patterns of PRiVENT’s social media activities. We emphasize that our findings relate to visibility rather than awareness.*
4. *Clarified methodological limitations:
   We added a dedicated section explaining that platform analytics do not measure cognitive or behavioral change. This limitation is now explicitly acknowledged and integrated.*

*We appreciate the reviewer’s sharp conceptual guidance and believe that these changes significantly strengthen the accuracy, clarity, and rigor of the manuscript.*

2. Lack of theoretical framework: The manuscript is descriptive and operational but theoretically underdeveloped. There is no engagement with communication theory, health literacy frameworks, public sphere theory, digital engagement models, or knowledge translation literature. For a paper that positions itself around “public awareness,” this absence is a major weakness. The introduction should be grounded in established frameworks of science communication, digital health communication, or misinformation research.

***Response:***

*We thank the reviewer for this valuable comment. We agree that the original introduction did not sufficiently reflect relevant theoretical perspectives. To address this, we have briefly expanded the introduction to reference key concepts from digital health communication and social media engagement. These additions provide a clearer rationale for analysing visibility and reach in the context of specialised health topics. Given the descriptive nature of our dataset, we focus on frameworks that are directly applicable without overstating the theoretical scope of the study. We hope this concise integration satisfactorily addresses the reviewer’s concern.*

3. Methodological limitation: purely descriptive analytics: The study design is retrospective and purely descriptive. There is no comparator (e.g., before–after, control topic, similar campaigns), no inferential analysis, no engagement rate calculation (per post, per follower), and no evaluation of content effectiveness beyond raw counts. Without normalization or benchmarking, the 2.6 million views cannot be interpreted. The analysis lacks analytical depth and risks being perceived as a campaign report rather than research.

***Response:*** *We appreciate the reviewer’s observation regarding the descriptive nature of our analysis. Our dataset is limited to retrospective platform analytics, which do not allow for inferential comparisons, benchmarking, or content-effectiveness analyses. We have therefore added a clear statement to the Limitations section emphasising that our results represent descriptive visibility metrics only, that the view counts cannot be interpreted without normalization or comparators, and that the analysis should not be understood as an effectiveness evaluation. We hope this clarification appropriately contextualises the methodological scope of the study.*

4. Weak operationalization of target groups: The manuscript assumes that certain demographic distributions (e.g., young female Instagram users) indicate successful reach of nurses. This inference is speculative. No direct occupational verification exists. Similarly, LinkedIn professional categories are interpreted as specific healthcare roles without validation. The conclusions about reaching intended target groups are not sufficiently supported by the data.

***Response:*** *We thank the reviewer for this important comment. We agree that the platform-based demographic information does not allow us to reliably infer occupational groups, and that any interpretation regarding specific professions (e.g., nurses) would be speculative. We have therefore revised the manuscript to remove such inferences and now describe the demographic data strictly as platform-reported user distributions. The conclusions have been adjusted accordingly to state that different demographic groups were reached, without attributing these groups to specific professional roles. We hope this clarification adequately addresses the reviewer’s concern.*

5. Limited contribution: From a scholarly perspective, the contribution is unclear. Many health projects use social media; descriptive reporting of output volume and views is not, in itself, novel. The manuscript needs to clarify what new knowledge this study generates for digital health communication, implementation science, or public engagement research. At present, it reads as documentation of project dissemination activities.

***Response:***
*We appreciate the reviewer’s concern regarding the contribution of the study. We agree that many health projects use social media and that descriptive metrics alone are not novel. However, we believe the study still provides meaningful value for digital health communication and implementation practice. It documents, for the first time, how a national weaning project in Germany—focused on a highly specialised and often overlooked topic—was able to reach large and diverse audiences, including unexpected demographic groups. The findings offer empirical insight into which types of content were most accessed and where information needs appear to exist.While exploratory, this evidence can support future implementation strategies for similar specialised medical fields, where public awareness has historically been low. We have clarified this contribution in the revised manuscript and explicitly acknowledged the study’s limitations.*

6. Discussion lacks critical reflection: The discussion remains largely affirmative. There is minimal critical interrogation of:
• algorithmic bias.
• paid promotion effects.
• regional targeting limitations.
• sustainability of engagement.
• potential echo-chamber effects.
• cost-effectiveness.
Hence, a more rigorous critical appraisal is required.

***Response:*** *We thank the reviewer for these important comments. We agree that the study has several methodological and conceptual limitations, including the indirect identification of user groups, the descriptive design, and the limited ability to assess algorithmic effects, sustainability, or cost-effectiveness. We have now revised the limitations section accordingly and clearly outlined these constraints. In addition, we have strengthened the discussion to better delineate the study’s contribution—namely, empirical insights into the feasibility and reach of a collaborative digital health communication approach—and to specify where more robust research is needed. We hope that these revisions adequately address the reviewer’s concerns.*

Overall assessment: The topic is relevant and socially meaningful. The execution of the campaign appears substantial. However, as a scientific manuscript, it currently overinterprets descriptive analytics and lacks theoretical and methodological depth. Substantial revision is required to align claims with evidence and to clarify the scholarly contribution.

***Response:****Thank you for this overall assessment. We appreciate the acknowledgement of the social relevance and the substantial execution of the campaign. We also agree that the original manuscript tended to overinterpret descriptive findings and did not sufficiently articulate its theoretical and methodological boundaries. In the revision, we have aligned all claims more closely with the available evidence, strengthened the positioning within existing frameworks of digital health communication, and clarified the exploratory nature of the analysis. We now explicitly outline the study’s scholarly contribution as well as its limitations.*
