# Supplementary material for: Social media activities to enhance the visibility of long-term invasive ventilation: A content and visibility analysis from the PRiVENT study
Source: PLoS One. 2026 Jun 25;21(6):e0352267. doi: 10.1371/journal.pone.0352267 (PMC13298930; doi:10.1371/journal.pone.0352267)
Supplement: Attachment — Submitted filename: Point_by_point_auresp_4.docx [file pone.0352267.s008.docx]

Dear Editor and Reviewers,

we would like to thank you for the thorough and constructive evaluation of our manuscript. We highly appreciate the time and effort invested in reviewing our work.

We have carefully considered all comments and revised the manuscript accordingly. All reviewer comments are reproduced below, and our responses are provided in blue font. Changes in the manuscript are indicated accordingly.

We have in particular clarified the descriptive nature of the study, strengthened methodological transparency, expanded the Discussion with a more critical reflection, and more clearly positioned the contribution within the digital health communication literature. Limitations have been addressed in greater detail, and future research needs have been outlined. Aditionally, an anonymized, aggregated dataset has been made publicly available via Zenodo.

Please find below our detailed, point-by-point responses.

Thank you again for your valuable feedback and for the opportunity to improve our manuscript.

Sincerely,
Franziska Trudzinski et al.

**Comments to the Author**

1. If the authors have adequately addressed your comments raised in a previous round of review and you feel that this manuscript is now acceptable for publication, you may indicate that here to bypass the “Comments to the Author” section, enter your conflict of interest statement in the “Confidential to Editor” section, and submit your "Accept" recommendation.

Reviewer #5: (No Response)

Reviewer #6: All comments have been addressed

*Answer: We thank the reviewer for the constructive feedback. We have addressed all comments raised in the previous review round and revised the manuscript accordingly. We hope that the manuscript is now suitable for publication.*

2. Is the manuscript technically sound, and do the data support the conclusions?

The manuscript must describe a technically sound piece of scientific research with data that supports the conclusions. Experiments must have been conducted rigorously, with appropriate controls, replication, and sample sizes. The conclusions must be drawn appropriately based on the data presented.

Reviewer #5: Partly

Reviewer #6: Yes

*Answer:* *We thank the reviewer for this important comment. We confirm that the study was conducted with appropriate methodological rigor, including suitable controls, and that the sample size and analyses are in line with the study design. The conclusions have been carefully derived from the data presented and have been revised where necessary to ensure they are fully supported by the results.*

3. Has the statistical analysis been performed appropriately and rigorously?

Reviewer #5: I Don't Know

Reviewer #6: Yes

4. Have the authors made all data underlying the findings in their manuscript fully available?

The [PLOS Data policy](http://track.editorialmanager.com/CL0/http:%2F%2Fwww.plosone.org%2Fstatic%2Fpolicies.action%23sharing/1/010f019d03e1700a-920f9ab1-22e9-41d0-81c7-e64d3e45bc67-000000/Xr6i2J6BYW2B9YY0_QwJiWEsjd_MZZ1oGGQ_ID3Wj28=255) requires authors to make all data underlying the findings described in their manuscript fully available without restriction, with rare exception (please refer to the Data Availability Statement in the manuscript PDF file). The data should be provided as part of the manuscript or its supporting information, or deposited to a public repository. For example, in addition to summary statistics, the data points behind means, medians and variance measures should be available. If there are restrictions on publicly sharing data—e.g. participant privacy or use of data from a third party—those must be specified.

Reviewer #5: Yes

Reviewer #6: Yes

5. Is the manuscript presented in an intelligible fashion and written in standard English?

PLOS ONE does not copyedit accepted manuscripts, so the language in submitted articles must be clear, correct, and unambiguous. Any typographical or grammatical errors should be corrected at revision, so please note any specific errors here.

Reviewer #5: Yes

Reviewer #6: Yes

6. Review Comments to the Author

Reviewer #3: The manuscript addresses a socially relevant and underexplored topic, namely the role of social media in increasing the visibility of long-term invasive mechanical ventilation (IMV). The authors present a substantial communication effort through the PRiVENT project, and the dataset spans multiple years and platforms, which is commendable. The revision has improved the manuscript, particularly in aligning claims with the descriptive nature of the data. However, several critical issues remain that limit the scientific contribution of the study.

*Anwer: Thank you for your thoughtful and constructive feedback. We appreciate your recognition of the relevance of the topic and the improvements made in the revision. We have carefully addressed the remaining concerns and further clarified scope, methods, and interpretation accordingly.*

Major Comments
1. Conceptual Framing and Claims
The authors have improved the terminology by shifting from “awareness” to “visibility” and “reach,” which is appropriate. However, some residual interpretative statements still imply user interest or informational needs based solely on view counts. These interpretations should be further softened, as platform analytics cannot capture cognitive engagement, understanding, or behavioral impact.

*The manuscript has been thoroughly revised and the respective sections have been adjusted accordingly.*

2. Methodological Limitations and Analytical Depth
The study remains purely descriptive. While the authors acknowledge this limitation, the manuscript still lacks analytical depth expected for a research article. Specifically:
- No normalization of metrics (e.g., engagement rate per post or per follower) is provided.
- No temporal or comparative analysis (e.g., trends over time, pre/post campaign phases) is included.
- No benchmarking against similar campaigns or baseline metrics is attempted.
As a result, the reported figures (e.g., 2.6 million views) remain difficult to interpret in a meaningful scientific context.

*Anwer: Thank you for these important and well-founded comments. We fully agree with the limitations you raise and have addressed them more explicitly in the revised manuscript.Regarding normalization of metrics, this remains challenging, as the included platforms use fundamentally different definitions and calculation methods for reach and engagement, limiting comparability across channels. We have clarified this point in the Methods and Discussion.With respect to temporal analyses, we now explicitly refer to Figure X, which illustrates trends over time within platforms. However, we agree that more robust pre/post comparisons are not feasible: the information offering was newly established within the PRiVENT project, and no pre-campaign phase exists. In addition, the platforms are still actively maintained, so a defined post-campaign phase cannot yet be analyzed.Benchmarking against similar campaigns or baseline metrics is likewise not possible, as— to our knowledge—no comparable structured program in this field currently exists in Germany, and baseline data are lacking.We have incorporated all these aspects more clearly into the Discussion as key limitations and outlined corresponding needs for future research, particularly regarding standardized metrics, longitudinal evaluation frameworks, and comparative analyses.*

3. Statistical Analysis
The absence of inferential or even basic comparative statistical analysis significantly weakens the rigor of the study. Even within a descriptive framework, additional analyses (e.g., distribution comparisons across platforms or content types) could strengthen the manuscript.

Anwer: Thank you for this important point. We agree that the absence of inferential or comparative statistical analyses limits the rigor of the study. At the same time, additional comparative analyses are only feasible to a limited extent in this context. As outlined above, the different platforms apply heterogeneous and non-transparent definitions of key metrics (e.g., views, reach, engagement), which restricts comparability across platforms and limits the validity of distributional comparisons. We have clarified this constraint more explicitly in the revised Methods and Discussion.Within these boundaries, we have strengthened the descriptive analysis where possible and now more clearly highlight temporal developments (see Figure 2). However, more advanced comparative approaches (e.g., across content types or platforms) remain methodologically challenging given the lack of standardized metrics and a consistent baseline. We have incorporated these aspects into the Discussion as important limitations and outlined the need for future research focusing on standardized reporting frameworks and more robust comparative designs.

4. Operationalization of Target Groups
Although the authors have removed earlier overinterpretations, the discussion still occasionally suggests inferred audience characteristics (e.g., healthcare professionals, nurses) without direct evidence. Platform-derived demographic and professional data should be treated with caution and clearly labeled as indirect indicators.

*Anwer: Thank you for this important observation. We agree that such interpretations require caution. In the revision, we have addressed this by consistently avoiding unsupported inferences and by clearly labeling any platform-derived demographic or professional information as indirect indicators. We have further revised the Discussion to ensure that audience characteristics are not overstated and remain aligned with the descriptive nature of the data.*

5. Scholarly Contribution
The manuscript still struggles to clearly articulate its novel contribution. While documenting a large-scale communication effort is valuable, the authors should more explicitly position their findings within digital health communication literature. What transferable insights does this study offer beyond descriptive reporting? This point requires further strengthening.

*Anwer: hank you for this important comment. We agree that the novel contribution of the study needs to be articulated more clearly.In the revision, we have strengthened the positioning of our study within the digital health communication literature and more explicitly outlined its contribution. To the best of our knowledge, this study represents the first structured, multi-platform digital communication approach addressing the highly specialized field of long-term mechanical ventilation in Germany. As such, it should primarily be understood as a hypothesis-generating and exploratory analysis rather than a confirmatory evaluation.Beyond descriptive reporting, we highlight (i) the feasibility of developing and implementing a structured, multi-platform communication strategy for a previously underrepresented patient group, (ii) the methodological challenges related to non-standardized and non-transparent platform metrics and their implications for evaluation, and (iii) practical considerations for longitudinal, cross-platform outreach in a clinical research context.The Discussion has been revised accordingly to better emphasize these aspects and to clarify how our findings may inform the design of future studies, particularly with regard to more rigorous, comparative, and theory-driven evaluation frameworks.*

6. Discussion and Critical Reflection
The discussion has improved but remains somewhat limited in critical depth. Important aspects are still underdeveloped, including:
- Algorithmic bias and platform-specific visibility mechanisms
- The role of paid promotion versus organic reach
- Sustainability of engagement over time
- Potential echo-chamber effects
- Cost-effectiveness of the campaign
A more critical and reflective discussion would significantly enhance the manuscript’s academic value.

*Anwer:Thank you for this valuable and insightful feedback. We agree that these aspects are highly relevant and that a more critical and reflective discussion strengthens the manuscript.In the revision, we have expanded the Discussion accordingly. Specifically, we now address the potential influence of algorithmic bias and platform-specific visibility mechanisms, acknowledging that content reach is shaped by non-transparent algorithms. We also clarify that our analysis is based predominantly on organic reach.Furthermore, we have added a more critical reflection on the sustainability of engagement over time, referring to the temporal trends shown in Figure 2, while noting the current lack of a defined post-campaign phase due to ongoing platform activity. We also discuss the potential for echo-chamber effects, particularly in health-related social media communication, and the associated limitations in reaching broader or less engaged audiences.Regarding cost-effectiveness, we clarify in the revision that a formal evaluation of the overall PRiVENT intervention is planned. To avoid unwarranted assumptions, we deliberately refrain from providing estimates or interpretations at this stage and instead highlight this as an important area for future research.All these aspects have been incorporated into the revised Discussion as key limitations and avenues for further investigation, thereby strengthening the critical depth and contextualization of our findings.*

Minor Comments
- Some grammatical inconsistencies and minor language issues remain and should be carefully proofread.
- The Methods section would benefit from clearer structuring, particularly in distinguishing between content production and analytical procedures.
- The Data Availability Statement does not fully comply with open data requirements; providing at least anonymized aggregated datasets in a repository would improve transparency.The manuscript has improved compared to previous versions and addresses a relevant topic. However, it still requires moderate to major revision to enhance methodological rigor, analytical depth, and clarity of scholarly contribution before it can be considered suitable for publication.

*Anwer: We have carefully proofread the manuscript and corrected remaining grammatical inconsistencies and minor language issues. The Methods section has been restructured to more clearly distinguish between content production and analytical procedures. An anonymized, aggregated dataset has been made publicly available via Zenodo (*[*https://zenodo.org/records/20035681*](https://zenodo.org/records/20035681)*)*

Reviewer #6: Thank you for the revision. The manuscript has improved, especially by narrowing the claim from “raising awareness” to “enhancing visibility.” This better matches the available data, which mainly include views, reach, impressions, followers, and platform user profiles.
The study remains descriptive and does not directly measure awareness, knowledge change, patient outcomes, or behavioral effects. Its theoretical and methodological contributions are therefore limited. However, the manuscript documents a meaningful practice in digital health communication. The PRiVENT project produced substantial public-facing content, including blog posts, social media posts, and podcast episodes, on long-term invasive mechanical ventilation, a highly specialized and often overlooked topic.
I especially appreciate the practical value of providing plain-language information for patients, relatives, and healthcare professionals. The strong interest in basic explanatory content, such as weaning and invasive ventilation, suggests a real need for accessible information in this field.
I believe that the manuscript has now met the requirements for publication.

*Anwer: Thank you again for your constructive feedback and for supporting the publication of our work.*
