# Supplementary material for: Social media activities to enhance the visibility of long-term invasive ventilation: A content and visibility analysis from the PRiVENT study
Source: PLoS One. 2026 Jun 25;21(6):e0352267. doi: 10.1371/journal.pone.0352267 (PMC13298930; doi:10.1371/journal.pone.0352267)
Supplement: Attachment — Submitted filename: Plos One social media.docx [file pone.0352267.s007.docx]

SOCIAL MEDIA ACTIVITIES TO ENHANCE THE VISIBILITY OF LONG-TERM INVASIVE VENTILATION: A CONTENT AND VISIBILITY ANALYSIS FROM THE PRIVENT STUDY

The manuscript addresses an important and underexplored topic, namely the use of social media and digital communication strategies to enhance the visibility of long-term invasive mechanical ventilation (IMV) and ventilator weaning. The PRiVENT project represents a substantial multi-platform communication effort conducted over several years and involving different stakeholder groups. The topic is socially relevant and the manuscript provides valuable practical insights into digital health communication in a highly specialized medical field.

The revised version has improved considerably compared with earlier versions, particularly through the more appropriate use of the terms “visibility” and “reach” instead of “awareness,” which better reflects the descriptive nature of the available data. The authors also provide a more balanced discussion of the study limitations.

However, several issues still require further refinement before publication.

Major comments

1. Clarification of scholarly contribution
   Although the manuscript documents a meaningful communication initiative, the scientific contribution remains somewhat unclear. The study is primarily descriptive and resembles a project report in several sections. The authors should further strengthen the positioning of the manuscript within the broader digital health communication literature and more explicitly explain what transferable scientific insights can be derived from this work beyond reporting platform metrics and content dissemination activities.
2. Overinterpretation of platform metrics
   The manuscript has improved substantially in avoiding unsupported claims; however, some statements still imply audience needs or audience composition without direct evidence. For example, statements suggesting that the observed access patterns indicate an “unmet need” or that specific professional groups were reached should be softened further. Platform analytics and view counts cannot reliably establish user motivations, informational needs, or professional identities. More cautious wording such as “may suggest” or “could reflect” would be more appropriate.
3. Limited analytical depth
   The analysis remains largely descriptive. While the limitations of platform-specific metrics are acknowledged, the manuscript would benefit from additional analytical depth where feasible. Even within a descriptive framework, the authors could further discuss comparative performance across content categories or provide more structured interpretation of longitudinal developments shown in Figure 2. At present, the manuscript relies heavily on absolute numbers without deeper contextual interpretation.
4. Theoretical framing
   The introduction would benefit from stronger integration of digital health communication theory. The manuscript briefly refers to digital engagement and health information seeking, but these concepts are not sufficiently developed throughout the paper. A clearer theoretical framing would strengthen the academic value of the study and help contextualize the findings within existing communication research.

Minor comments

1. Several grammatical inconsistencies and typographical issues remain throughout the manuscript and should be carefully proofread.
2. There are occasional formatting inconsistencies, including spacing and punctuation issues.
3. A clear reference mismatch appears in reference 10 (“Partial crowns as bridge abutments”), which seems unrelated to the topic of the manuscript and should be corrected.
4. Some figure legends could be improved for clarity and readability.
5. The manuscript would benefit from minor language polishing by a native English speaker or professional editing service.

Overall assessment

Overall, this manuscript addresses a relevant and specialized topic and provides useful practical insights into digital communication strategies in the field of long-term invasive mechanical ventilation. While the study remains primarily descriptive and has limited analytical depth, the revisions have substantially improved the manuscript’s balance and transparency. With additional clarification of the scholarly contribution, careful reduction of residual overinterpretation, and minor editorial corrections, the manuscript would be suitable for publication.
