# Supplementary material for: Social media activities to enhance the visibility of long-term invasive ventilation: A content and visibility analysis from the PRiVENT study
Source: PLoS One. 2026 Jun 25;21(6):e0352267. doi: 10.1371/journal.pone.0352267 (PMC13298930; doi:10.1371/journal.pone.0352267)
Supplement: Attachment — Submitted filename: Plos One social media point by point.docx [file pone.0352267.s009.docx]

**Dear Editor and Reviewers,**

We would like to sincerely thank you for the opportunity to revise and resubmit our manuscript and for the additional time and effort invested in the renewed evaluation of our work. We greatly appreciate the thoughtful and constructive comments, which have helped us to further improve the manuscript.

We have carefully considered all comments and revised the manuscript accordingly. Reviewer comments are reproduced below, followed by our detailed responses. All corresponding changes have been incorporated into the revised manuscript and are highlighted where appropriate.

Following the reviewers’ additional recommendations, we have carefully revised the manuscript and addressed all remaining comments. The revisions primarily focused on further improving methodological clarity, refining the presentation of the results, and strengthening the discussion and interpretation of the findings. We believe that these changes have further enhanced the overall quality, transparency, and readability of the manuscript.

We hope that the revisions satisfactorily address the remaining concerns and that the manuscript is now suitable for publication. We are grateful for the constructive feedback and for the opportunity to further improve our work.

Please find our detailed point-by-point responses below.

**Sincerely,**

**Franziska C. Trudzinski, on behalf of all co-authors**

SOCIAL MEDIA ACTIVITIES TO ENHANCE THE VISIBILITY OF LONG-TERM INVASIVE VENTILATION: A CONTENT AND VISIBILITY ANALYSIS FROM THE PRIVENT STUDY

The manuscript addresses an important and underexplored topic, namely the use of social media and digital communication strategies to enhance the visibility of long-term invasive mechanical ventilation (IMV) and ventilator weaning. The PRiVENT project represents a substantial multi-platform communication effort conducted over several years and involving different stakeholder groups. The topic is socially relevant and the manuscript provides valuable practical insights into digital health communication in a highly specialized medical field.

The revised version has improved considerably compared with earlier versions, particularly through the more appropriate use of the terms “visibility” and “reach” instead of “awareness,” which better reflects the descriptive nature of the available data. The authors also provide a more balanced discussion of the study limitations.

However, several issues still require further refinement before publication.

Response:

We sincerely thank the reviewer for the positive and encouraging assessment of our work. We greatly appreciate the recognition of the relevance of the topic, the substantial communication efforts within the PRiVENT project, and the practical contribution of the study to the field of digital health communication. We are also grateful for the acknowledgment that the revised manuscript has improved. We have carefully considered the remaining comments and addressed each point in detail below. We believe that these additional revisions have further strengthened the manuscript and improved its clarity and scientific rigor.

Major comments

1. Clarification of scholarly contribution
   Although the manuscript documents a meaningful communication initiative, the scientific contribution remains somewhat unclear. The study is primarily descriptive and resembles a project report in several sections. The authors should further strengthen the positioning of the manuscript within the broader digital health communication literature and more explicitly explain what transferable scientific insights can be derived from this work beyond reporting platform metrics and content dissemination activities.

**Response:**We thank the reviewer for this valuable comment. We agree that the primary purpose of the study is descriptive and that the manuscript reports the implementation and performance of a multi-platform communication strategy within the PRiVENT project. To better highlight the scientific contribution beyond the reporting of communication metrics, we have revised the Discussion section and more explicitly positioned our findings within the context of digital health communication research.In particular, we now emphasize that our findings provide transferable insights into the implementation of digital communication strategies in highly specialised healthcare settings. The analysis demonstrates that different social media platforms reach distinct stakeholder groups and generate different patterns of engagement, suggesting that a coordinated multi-channel approach may be required to effectively communicate complex healthcare topics to patients, relatives, healthcare professionals, researchers, and the general public. We further discuss how these observations may inform the design of future dissemination and stakeholder engagement strategies in healthcare research projects and specialised medical disciplines.

Accordingly, the following text has been added to the Discussion "Beyond documenting communication metrics, our findings provide practical insights into the implementation of digital communication strategies in highly specialised healthcare settings. The observed differences in reach and engagement across platforms suggest that a coordinated multi-channel approach may be necessary to effectively address the diverse information needs of patients, relatives, healthcare professionals, researchers, and the broader public. These observations contribute to the growing literature on digital health communication by illustrating how different communication channels can complement each other when disseminating complex healthcare topics and engaging diverse stakeholder groups in a real-world setting."

2. Overinterpretation of platform metrics
   The manuscript has improved substantially in avoiding unsupported claims; however, some statements still imply audience needs or audience composition without direct evidence. For example, statements suggesting that the observed access patterns indicate an “unmet need” or that specific professional groups were reached should be softened further. Platform analytics and view counts cannot reliably establish user motivations, informational needs, or professional identities. More cautious wording such as “may suggest” or “could reflect” would be more appropriate.

**Response:** We thank the reviewer for this important comment and agree that platform analytics do not allow firm conclusions regarding user motivations, informational needs, or audience composition. We have therefore carefully revised the manuscript and adopted more cautious wording throughout. Statements implying certainty about audience characteristics or unmet informational needs have been softened, and demographic information derived from social media analytics is now explicitly described as platform-provided audience estimates rather than confirmed user characteristics. These revisions ensure that the interpretation remains fully consistent with the descriptive nature and limitations of the available data.

3. Limited analytical depth
   The analysis remains largely descriptive. While the limitations of platform-specific metrics are acknowledged, the manuscript would benefit from additional analytical depth where feasible. Even within a descriptive framework, the authors could further discuss comparative performance across content categories or provide more structured interpretation of longitudinal developments shown in Figure 2. At present, the manuscript relies heavily on absolute numbers without deeper contextual interpretation.

**Response:**We thank the reviewer for this valuable suggestion. We agree that, despite the descriptivenature of the study, additional contextual interpretation can help readers better understand the observed communication patterns.

In response, we have expanded the Results and Discussion sections to provide a more structured interpretation of the longitudinal developments shown in Figure 2 and the performance of different communication channels. Specifically, we now discuss that visibility was not distributed evenly over time but was characterised by several pronounced peaks, likely reflecting periods of intensified communication activity, publication-related dissemination, or particularly engaging content. We further highlight the complementary roles of different platforms, with Facebook and Instagram generating the highest overall reach, whereas LinkedIn contributed more consistently to professional visibility. Given the exploratory and descriptive design of the study, we deliberately refrained from conducting inferential analyses that would exceed the explanatory power of the available platform metrics. Nevertheless, the additional interpretation provides greater context for the observed trends while remaining consistent with the descriptive nature of the data.

4. Theoretical framing
   The introduction would benefit from stronger integration of digital health communication theory. The manuscript briefly refers to digital engagement and health information seeking, but these concepts are not sufficiently developed throughout the paper. A clearer theoretical framing would strengthen the academic value of the study and help contextualize the findings within existing communication research.

**Response:**We thank the reviewer for this valuable suggestion and agree that a clearer theoretical framing strengthens the interpretation and contextualisation of our findings. In response, we have expanded the Introduction to more explicitly position the study within the field of digital health communication.Specifically, we now discuss the role of social media as a tool for health information dissemination, stakeholder engagement, and increasing the visibility of healthcare topics. We further introduce concepts of health information seeking and digital engagement to provide a conceptual framework for understanding how different digital platforms may contribute to the communication of specialised healthcare topics and reach diverse audiences. These additions help place the PRiVENT communication activities within the broader digital health communication literature and provide a clearer theoretical context for the interpretation of the observed visibility, reach, and engagement patterns.

Minor comments

1. Several grammatical inconsistencies and typographical issues remain throughout the manuscript and should be carefully proofread.
2. There are occasional formatting inconsistencies, including spacing and punctuation issues.

**Response:** We thank the reviewer for these comments. The manuscript has been carefully proofread and revised to address grammatical inconsistencies, typographical errors, as well as minor formatting issues, including spacing and punctuation. These revisions were made throughout the manuscript to improve consistency, clarity, and readability.

3. A clear reference mismatch appears in reference 10 (“Partial crowns as bridge abutments”), which seems unrelated to the topic of the manuscript and should be corrected.

**Response:** We thank the reviewer for identifying this error. Reference 10 was incorrectly listed due to a citation management issue and was unrelated to the topic of the manuscript. The reference has been corrected in the revised version, and we have carefully checked the reference list to ensure the accuracy and consistency of all citations.

4. Some figure legends could be improved for clarity and readability.

**Response:**We thank the reviewer for this helpful suggestion. We have revised the figure legends to improve clarity and readability. Additional explanatory information has been provided where appropriate, and the wording has been refined to facilitate interpretation of the figures without requiring extensive reference to the main text.

5. The manuscript would benefit from minor language polishing by a native English speaker or professional editing service.

**Response:**We thank the reviewer for this suggestion. The manuscript has been carefully proofread and comprehensively revised. Particular attention was paid to language, style, grammar, consistency, and readability throughout the text. We believe that these revisions have substantially improved the overall quality and clarity of the manuscript.

Overall assessment

Overall, this manuscript addresses a relevant and specialized topic and provides useful practical insights into digital communication strategies in the field of long-term invasive mechanical ventilation. While the study remains primarily descriptive and has limited analytical depth, the revisions have substantially improved the manuscript’s balance and transparency. With additional clarification of the scholarly contribution, careful reduction of residual overinterpretation, and minor editorial corrections, the manuscript would be suitable for publication.

**Response:**We sincerely thank the reviewer for the careful evaluation of our manuscript and for the constructive comments provided throughout the review process. We appreciate the recognition of the relevance of the topic and the practical contribution of the study to the field of digital health communication. We have carefully addressed all remaining comments by further clarifying the scholarly contribution of the work, adopting more cautious interpretations of platform metrics where appropriate, strengthening the theoretical framing, and implementing the suggested editorial corrections. We believe that these revisions have further improved the clarity, transparency, and overall quality of the manuscript, and we are grateful for the reviewer’s positive assessment and recommendation for publication.
