# Supplementary material for: Social media activities to enhance the visibility of long-term invasive ventilation: A content and visibility analysis from the PRiVENT study
Source: PLoS One. 2026 Jun 25;21(6):e0352267. doi: 10.1371/journal.pone.0352267 (PMC13298930; doi:10.1371/journal.pone.0352267)
Supplement: Peer review history

# Peer review: Decision Letter 0

**DOI:** 10.1371/journal.pone.0352267.r001

**Authors:** Jianhong Zhou

13 Oct 2025

PONE-D-25-24730

The use of social media in PRiVENT to raise awareness of long-term invasive ventilation

PLOS ONE

Dear Dr. Michels-Zetsche,

Thank you for submitting your manuscript to PLOS ONE. After careful consideration, we feel that it has merit but does not fully meet PLOS ONE’s publication criteria as it currently stands. Therefore, we invite you to submit a revised version of the manuscript that addresses the points raised during the review process.

Please submit your revised manuscript by Nov 22 2025 11:59PM. If you will need more time than this to complete your revisions, please reply to this message or contact the journal office at plosone@plos.org. When you're ready to submit your revision, log on to https://www.editorialmanager.com/pone/ and select the 'Submissions Needing Revision' folder to locate your manuscript file.

Please include the following items when submitting your revised manuscript:

- A rebuttal letter that responds to each point raised by the academic editor and reviewer(s). You should upload this letter as a separate file labeled 'Response to Reviewers'.
- A marked-up copy of your manuscript that highlights changes made to the original version. You should upload this as a separate file labeled 'Revised Manuscript with Track Changes'.
- An unmarked version of your revised paper without tracked changes. You should upload this as a separate file labeled 'Manuscript'.

-->If you would like to make changes to your financial disclosure, please include your updated statement in your cover letter. Guidelines for resubmitting your figure files are available below the reviewer comments at the end of this letter.

If applicable, we recommend that you deposit your laboratory protocols in protocols.io to enhance the reproducibility of your results. Protocols.io assigns your protocol its own identifier (DOI) so that it can be cited independently in the future. For instructions see: https://journals.plos.org/plosone/s/submission-guidelines#loc-laboratory-protocols. Additionally, PLOS ONE offers an option for publishing peer-reviewed Lab Protocol articles, which describe protocols hosted on protocols.io. Read more information on sharing protocols at https://plos.org/protocols?utm_medium=editorial-email&utm_source=authorletters&utm_campaign=protocols.

We look forward to receiving your revised manuscript.

Kind regards,

Jianhong Zhou

Staff Editor

PLOS ONE

Journal Requirements:

When submitting your revision, we need you to address these additional requirements.

1. Please ensure that your manuscript meets PLOS ONE's style requirements, including those for file naming. The PLOS ONE style templates can be found at

https://journals.plos.org/plosone/s/file?id=wjVg/PLOSOne_formatting_sample_main_body.pdf and

https://journals.plos.org/plosone/s/file?id=ba62/PLOSOne_formatting_sample_title_authors_affiliations.pdf

2. We note that the grant information you provided in the ‘Funding Information’ and ‘Financial Disclosure’ sections do not match.

When you resubmit, please ensure that you provide the correct grant numbers for the awards you received for your study in the ‘Funding Information’ section.

3. Thank you for stating the following financial disclosure:

“Funding by the Innovation Fund of the Federal Joint Committee (G-BA), grant number: 01NVF19023.”

Please state what role the funders took in the study.  If the funders had no role, please state: "The funders had no role in study design, data collection and analysis, decision to publish, or preparation of the manuscript." If this statement is not correct you must amend it as needed.

Please include this amended Role of Funder statement in your cover letter; we will change the online submission form on your behalf.

4. Please note that funding information should not appear in the Acknowledgments section or other areas of your manuscript. We will only publish funding information present in the Funding Statement section of the online submission form. Please remove any funding-related text from the manuscript.

5. Thank you for stating the following in the Competing Interests section:

“FCT reports payment or honoraria for lectures, or reimbursement of travel expenses from Novartis AG, GlaxoSmithKline, Chiesi, Boehringer Ingelheim GmbH, Grifols and AstraZeneca. JDMZ payment or honoraria for lectures, or reimbursement of travel expenses from CSL Behring and AstraZeneca. All authors declare funding by the Innovation Fund of the Federal Joint Committee (G-BA), grant number: 01NVF19023.”

We note that you received funding from a commercial source: Novartis AG, GlaxoSmithKline, Chiesi, Boehringer Ingelheim GmbH, Grifols, CSL Behring and AstraZeneca.

Please provide an amended Competing Interests Statement that explicitly states this commercial funder, along with any other relevant declarations relating to employment, consultancy, patents, products in development, marketed products, etc.

Within this Competing Interests Statement, please confirm that this does not alter your adherence to all PLOS ONE policies on sharing data and materials by including the following statement: "This does not alter our adherence to PLOS ONE policies on sharing data and materials.” (as detailed online in our guide for authors http://journals.plos.org/plosone/s/competing-interests).  If there are restrictions on sharing of data and/or materials, please state these. Please note that we cannot proceed with consideration of your article until this information has been declared.

Please include your amended Competing Interests Statement within your cover letter. We will change the online submission form on your behalf.

6. We note that you have indicated that there are restrictions to data sharing for this study. For studies involving human research participant data or other sensitive data, we encourage authors to share de-identified or anonymized data. However, when data cannot be publicly shared for ethical reasons, we allow authors to make their data sets available upon request. For information on unacceptable data access restrictions, please see http://journals.plos.org/plosone/s/data-availability#loc-unacceptable-data-access-restrictions.

Before we proceed with your manuscript, please address the following prompts:

a) If there are ethical or legal restrictions on sharing a de-identified data set, please explain them in detail (e.g., data contain potentially identifying or sensitive patient information, data are owned by a third-party organization, etc.) and who has imposed them (e.g., a Research Ethics Committee or Institutional Review Board, etc.). Please also provide contact information for a data access committee, ethics committee, or other institutional body to which data requests may be sent.

b) If there are no restrictions, please upload the minimal anonymized data set necessary to replicate your study findings to a stable, public repository and provide us with the relevant URLs, DOIs, or accession numbers. Please see http://www.bmj.com/content/340/bmj.c181.long for guidelines on how to de-identify and prepare clinical data for publication. For a list of recommended repositories, please see https://journals.plos.org/plosone/s/recommended-repositories. You also have the option of uploading the data as Supporting Information files, but we would recommend depositing data directly to a data repository if possible.

Please update your Data Availability statement in the submission form accordingly.

7. In the online submission form you indicate that your data is not available for proprietary reasons and have provided a contact point for accessing this data. Please note that your current contact point is a co-author on this manuscript. According to our Data Policy, the contact point must not be an author on the manuscript and must be an institutional contact, ideally not an individual. Please revise your data statement to a non-author institutional point of contact, such as a data access or ethics committee, and send this to us via return email. Please also include contact information for the third party organization, and please include the full citation of where the data can be found.

8. Thank you for stating the following in the Financial Disclosure section:

“Funding by the Innovation Fund of the Federal Joint Committee (G-BA), grant number: 01NVF19023.”

We note that one or more of the authors are employed by a commercial company: “Visuelle Werte”

1) Please provide an amended Funding Statement declaring this commercial affiliation, as well as a statement regarding the Role of Funders in your study. If the funding organization did not play a role in the study design, data collection and analysis, decision to publish, or preparation of the manuscript and only provided financial support in the form of authors' salaries and/or research materials, please review your statements relating to the author contributions, and ensure you have specifically and accurately indicated the role(s) that these authors had in your study. You can update author roles in the Author Contributions section of the online submission form.

Please also include the following statement within your amended Funding Statement.

“The funder provided support in the form of salaries for authors [insert relevant initials], but did not have any additional role in the study design, data collection and analysis, decision to publish, or preparation of the manuscript. The specific roles of these authors are articulated in the ‘author contributions’ section.”

If your commercial affiliation did play a role in your study, please state and explain this role within your updated Funding Statement.

2) Please also provide an updated Competing Interests Statement declaring this commercial affiliation along with any other relevant declarations relating to employment, consultancy, patents, products in development, or marketed products, etc.

Within your Competing Interests Statement, please confirm that this commercial affiliation does not alter your adherence to all PLOS ONE policies on sharing data and materials by including the following statement: ""This does not alter our adherence to  PLOS ONE policies on sharing data and materials.” (as detailed online in our guide for authors http://journals.plos.org/plosone/s/competing-interests) . If this adherence statement is not accurate and  there are restrictions on sharing of data and/or materials, please state these. Please note that we cannot proceed with consideration of your article until this information has been declared.

Please include both an updated Funding Statement and Competing Interests Statement in your cover letter. We will change the online submission form on your behalf.

9. One of the noted authors is a group or consortium: “the PRiVENT - Study Group”

In addition to naming the author group, please list the individual authors and affiliations within this group in the acknowledgments section of your manuscript. Please also indicate clearly a lead author for this group along with a contact email address.

10. Your ethics statement should only appear in the Methods section of your manuscript. If your ethics statement is written in any section besides the Methods, please move it to the Methods section and delete it from any other section. Please ensure that your ethics statement is included in your manuscript, as the ethics statement entered into the online submission form will not be published alongside your manuscript.

11. We note that Figure 1 includes an image of participants in the study.

As per the PLOS ONE policy (http://journals.plos.org/plosone/s/submission-guidelines#loc-human-subjects-research) on papers that include identifying, or potentially identifying, information, the individual(s) or parent(s)/guardian(s) must be informed of the terms of the PLOS open-access (CC-BY) license and provide specific permission for publication of these details under the terms of this license. Please download the Consent Form for Publication in a PLOS Journal (http://journals.plos.org/plosone/s/file?id=8ce6/plos-consent-form-english.pdf). The signed consent form should not be submitted with the manuscript, but should be securely filed in the individual's case notes. Please amend the methods section and ethics statement of the manuscript to explicitly state that the patient/participant has provided consent for publication: “The individual in this manuscript has given written informed consent (as outlined in PLOS consent form) to publish these case details”.

If you are unable to obtain consent from the subject of the photograph, you will need to remove the figure and any other textual identifying information or case descriptions for this individual.

12. Please include captions for your Supporting Information files at the end of your manuscript, and update any in-text citations to match accordingly. Please see our Supporting Information guidelines for more information: http://journals.plos.org/plosone/s/supporting-information.

13. If the reviewer comments include a recommendation to cite specific previously published works, please review and evaluate these publications to determine whether they are relevant and should be cited. There is no requirement to cite these works unless the editor has indicated otherwise.

14. Please review your reference list to ensure that it is complete and correct. If you have cited papers that have been retracted, please include the rationale for doing so in the manuscript text, or remove these references and replace them with relevant current references. Any changes to the reference list should be mentioned in the rebuttal letter that accompanies your revised manuscript. If you need to cite a retracted article, indicate the article’s retracted status in the References list and also include a citation and full reference for the retraction notice.

[Note: HTML markup is below. Please do not edit.]

Reviewers' comments:

Reviewer's Responses to Questions

**Comments to the Author**

1. Is the manuscript technically sound, and do the data support the conclusions?

The manuscript must describe a technically sound piece of scientific research with data that supports the conclusions. Experiments must have been conducted rigorously, with appropriate controls, replication, and sample sizes. The conclusions must be drawn appropriately based on the data presented.

Reviewer #1: Yes

Reviewer #2: Yes

Reviewer #3: Yes

Reviewer #4: Yes

**********

2. Has the statistical analysis been performed appropriately and rigorously?

Reviewer #1: Yes

Reviewer #2: Yes

Reviewer #3: Yes

Reviewer #4: Yes

**********

3. Have the authors made all data underlying the findings in their manuscript fully available?

The PLOS Data policy requires authors to make all data underlying the findings described in their manuscript fully available without restriction, with rare exception (please refer to the Data Availability Statement in the manuscript PDF file). The data should be provided as part of the manuscript or its supporting information, or deposited to a public repository. For example, in addition to summary statistics, the data points behind means, medians and variance measures should be available. If there are restrictions on publicly sharing data—e.g. participant privacy or use of data from a third party—those must be specified.

Reviewer #1: Yes

Reviewer #2: Yes

Reviewer #3: Yes

Reviewer #4: Yes

**********

4. Is the manuscript presented in an intelligible fashion and written in standard English?

PLOS ONE does not copyedit accepted manuscripts, so the language in submitted articles must be clear, correct, and unambiguous. Any typographical or grammatical errors should be corrected at revision, so please note any specific errors here.

Reviewer #1: Yes

Reviewer #2: Yes

Reviewer #3: Yes

Reviewer #4: Yes

**********

5. Review Comments to the Author

Please use the space provided to explain your answers to the questions above. You may also include additional comments for the author, including concerns about dual publication, research ethics, or publication ethics. (Please upload your review as an attachment if it exceeds 20,000 characters)

Reviewer #1: The Privent project's use of social media to spread knowledge about long-term invasive mechanical ventilation is examined in this publication. The study presents a well-planned campaign that incorporates blogs, podcasts, and social media platforms, and it is timely and pertinent. Novelty, transparency, and public health significance are among its strong points. The use of descriptive statistics (views, impressions) in the absence of explicit indicators of empowerment or awareness, however, has drawbacks that need to be more explicitly recognized. Conclusions ought to be more circumspect, focusing on possible effects rather than validated results. Data accessibility might be enhanced. All things considered, this is a worthwhile addition that would be appropriate with only slight to moderate editing.

Reviewer #2: The manuscript tackles a significant and little-studied subject: using social media to increase awareness about long-term invasive mechanical ventilation. The approach is transparent about funding, ethics approval, and conflicting interests, and it is well explained. The findings are pertinent and show that various audiences were meaningfully engaged. The findings align with the information provided. There was no proof of publication misbehavior, ethical transgressions, or dual publication. By emphasizing the importance of digital communication in raising health awareness, the study provides a significant contribution. The manuscript would be strengthened even more with minor adjustments to the topic of generalizability and the clarity of the findings presentation.

Reviewer #3: The manuscript addresses an important and timely issue: raising public awareness of long-term invasive mechanical ventilation (IMV) through the PRiVENT social media campaign. The study is well-structured, the objectives are clear, and the descriptive analysis provides valuable insight into how social media can serve as a platform for disseminating health-related information. I commend the authors for including a multidisciplinary team and for highlighting the role of patients, relatives, and healthcare professionals in shaping the campaign.

Strengths:

The study is innovative in its focus on social media as a tool to engage both the general public and healthcare professionals in a sensitive medical topic.

The dataset is substantial, covering a four-year period with diverse outputs (blog posts, podcasts, and social media posts).

Results are clearly presented with supporting figures and tables, which help illustrate reach and demographic differences across platforms.

Ethical considerations were well-handled, and the data were anonymized appropriately.

Points for Improvement:

Data Availability: The manuscript states that datasets are available upon request. PLOS ONE requires full data availability unless there are clear legal or ethical restrictions. Please clarify why full public access is restricted and consider providing at least anonymized aggregated data in a repository.

Statistical Rigor: While descriptive statistics are appropriate for measuring reach and impressions, the analysis could be enhanced by including trend analyses (e.g., growth over time, engagement per type of content) or comparisons between platforms using simple statistical tests. This would strengthen the conclusions.

Impact Assessment: The manuscript focuses primarily on quantitative reach. It would be beneficial to discuss qualitative outcomes, such as evidence of increased awareness, behavioral change, or stakeholder engagement beyond clicks and views.

Literature Context: The discussion could be enriched by integrating more recent references (within the past 5 years) on health communication via social media, to situate the study more firmly within current discourse.

Language and Style: The manuscript is overall well-written, but some minor typographical and grammatical errors should be revised at copyediting (e.g., occasional redundancies in describing the platforms and content types).

Overall Assessment:

This manuscript makes a useful contribution to the field of health communication and respiratory medicine. With improvements in data transparency, statistical depth, and contextual discussion, it will provide even stronger evidence for the role of social media in addressing underrepresented healthcare topics.

Reviewer #4: This manuscript addresses an important and underexplored issue: raising public awareness of long-term invasive mechanical ventilation through social media. The study is relevant, timely, and well-presented, with a clear description of campaign activities and transparent reporting of reach metrics. Its interdisciplinary approach and innovative use of multiple media formats are commendable.

However, the manuscript focuses primarily on quantitative reach without sufficiently addressing qualitative impact, such as changes in knowledge, attitudes, or behaviors among patients, relatives, or healthcare staff. The Methods section is overly descriptive in parts, while the Discussion could be strengthened by deeper analysis, including comparison with similar health communication campaigns and exploration of broader policy implications.

Overall, the manuscript is valuable but requires minor revision to improve its scientific contribution and generalizability.

**********

6. PLOS authors have the option to publish the peer review history of their article (what does this mean?). If published, this will include your full peer review and any attached files.

If you choose “no”, your identity will remain anonymous but your review may still be made public.

**Do you want your identity to be public for this peer review?**    For information about this choice, including consent withdrawal, please see our Privacy Policy.

Reviewer #1: No

Reviewer #2: No

Reviewer #3: No

Reviewer #4: No

**********

[NOTE: If reviewer comments were submitted as an attachment file, they will be attached to this email and accessible via the submission site. Please log into your account, locate the manuscript record, and check for the action link "View Attachments". If this link does not appear, there are no attachment files.]

While revising your submission, please upload your figure files to the Preflight Analysis and Conversion Engine (PACE) digital diagnostic tool, https://pacev2.apexcovantage.com/. PACE helps ensure that figures meet PLOS requirements. To use PACE, you must first register as a user. Registration is free. Then, login and navigate to the UPLOAD tab, where you will find detailed instructions on how to use the tool. If you encounter any issues or have any questions when using PACE, please email PLOS at figures@plos.org. Please note that Supporting Information files do not need this step.

Attachment Submitted filename: PONE-D-25-24730_reviewer.pdf

# Author comment: Author response to Decision Letter 1

**DOI:** 10.1371/journal.pone.0352267.r002

12 Dec 2025

Response to Reviewers

We would like to thank the Academic Editor and the reviewers for their thorough and constructive feedback on our manuscript “The use of social media in PRiVENT to raise awareness of long-term invasive ventilation.” We carefully considered all comments and have revised the manuscript accordingly. The responses below address each point raised by the reviewers in detail. All changes made to the manuscript are highlighted in the revised version.

Reviewers’ comments:

Reviewers’ comments:

Reviewer #1: The Privent project's use of social media to spread knowledge about long-term invasive mechanical ventilation is examined in this publication. The study presents a well-planned campaign that incorporates blogs, podcasts, and social media platforms, and it is timely and pertinent. Novelty, transparency, and public health significance are among its strong points. The use of descriptive statistics (views, impressions) in the absence of explicit indicators of empowerment or awareness, however, has drawbacks that need to be more explicitly recognized. Conclusions ought to be more circumspect, focusing on possible effects rather than validated results. Data accessibility might be enhanced. All things considered, this is a worthwhile addition that would be appropriate with only slight to moderate editing.

Answer: We thank Reviewer #1 for the positive and constructive feedback. We appreciate the recognition of the study’s relevance, novelty, and public health significance. We acknowledge the limitation of relying on descriptive statistics without direct measures of empowerment or awareness and have revised the manuscript to discuss this more explicitly. The conclusions have been adjusted to adopt a more cautious tone, focusing on potential rather than confirmed effects. We also improved the section on data accessibility. We are grateful for the reviewer’s helpful comments, which have strengthened the manuscript.Thank you very much for this positive feedback and pointing out to stress the limitations of descriptive statistics, which we added in the discussion section. The conclusion was shortened.We hope this imprioved our manuscript.

Reviewer #2: The manuscript tackles a significant and little-studied subject: using social media to increase awareness about long-term invasive mechanical ventilation. The approach is transparent about funding, ethics approval, and conflicting interests, and it is well explained. The findings are pertinent and show that various audiences were meaningfully engaged. The findings align with the information provided. There was no proof of publication misbehavior, ethical transgressions, or dual publication. By emphasizing the importance of digital communication in raising health awareness, the study provides a significant contribution. The manuscript would be strengthened even more with minor adjustments to the topic of generalizability and the clarity of the findings presentation.

Answer:We thank Reviewer #2 for the positive and encouraging feedback and for acknowledging the study’s transparency, methodological rigor, and relevance. We appreciate the suggestion to further address the generalizability of our findings and have added a short discussion on this aspect.

Reviewer #3: The manuscript addresses an important and timely issue: raising public awareness of long-term invasive mechanical ventilation (IMV) through the PRiVENT social media campaign. The study is well-structured, the objectives are clear, and the descriptive analysis provides valuable insight into how social media can serve as a platform for disseminating health-related information. I commend the authors for including a multidisciplinary team and for highlighting the role of patients, relatives, and healthcare professionals in shaping the campaign.

Strengths:The study is innovative in its focus on social media as a tool to engage both the general public and healthcare professionals in a sensitive medical topic. The dataset is substantial, covering a four-year period with diverse outputs (blog posts, podcasts, and social media posts).Results are clearly presented with supporting figures and tables, which help illustrate reach and demographic differences across platforms.Ethical considerations were well-handled, and the data were anonymized appropriately.

Answer:We thank Reviewer #3 for the positive and thoughtful feedback and for recognizing the importance, structure, and clarity of our study. We greatly appreciate the acknowledgment of our multidisciplinary approach and the inclusion of patients, relatives, and healthcare professionals in shaping the PRiVENT campaign. We are pleased that the reviewer found the presentation of results, ethical considerations, and use of diverse communication formats to be clear and appropriate.We are grateful for these encouraging comments, which support the relevance and value of our work.

Points for Improvement:Data Availability: The manuscript states that datasets are available upon request. PLOS ONE requires full data availability unless there are clear legal or ethical restrictions. Please clarify why full public access is restricted and consider providing at least anonymized aggregated data in a repository.

Answer: We thank the reviewer for this valuable comment. The underlying data are derived from user interactions on social media platforms, which are subject to the platforms’ terms of use and data protection regulations. These legal and ethical restrictions prevent the public sharing of raw datasets, as user-level data cannot be fully anonymised without breaching platform policies.To ensure transparency, we have clarified this point in the Data Availability Statement. Summary data supporting the findings of this study can be requested from the corresponding author.

Statistical Rigor: While descriptive statistics are appropriate for measuring reach and impressions, the analysis could be enhanced by including trend analyses (e.g., growth over time, engagement per type of content) or comparisons between platforms using simple statistical tests. This would strengthen the conclusions.

Answer: We thank the reviewer for this valuable comment. The statistical approach was intentionally kept simple, as the primary aim of the study was to provide a descriptive overview of the campaign’s reach and engagement rather than to test specific hypotheses. Nevertheless, we acknowledge that more detailed analyses (e.g., trend or comparative analyses) could provide additional insights. We have therefore included this point as a limitation in the revised Discussion section and noted that future research could build on this by applying more advanced statistical methods

Impact Assessment: The manuscript focuses primarily on quantitative reach. It would be beneficial to discuss qualitative outcomes, such as evidence of increased awareness, behavioral change, or stakeholder engagement beyond clicks and views.

Answer: We thank the reviewer for this important suggestion. We agree that assessing qualitative outcomes such as increased awareness, behavioral change, or stakeholder engagement would provide valuable additional insights beyond quantitative reach. This limitation has been added to the revised Discussion section, where we note that future studies should incorporate qualitative or mixed-methods approaches to better evaluate the broader impact of social media-based communication efforts.

We thank the reviewer for this insightful comment. Unfortunately, the available data do not allow for a qualitative assessment of increased awareness, behavioral change, or stakeholder engagement beyond quantitative metrics such as reach and impressions.

Answer: We thank the reviewer for this important suggestion. We agree that assessing qualitative outcomes such as increased awareness, behavioral change, or stakeholder engagement would provide valuable additional insights beyond quantitative reach. This limitation has been added to the revised Discussion section, where we note that future studies should incorporate qualitative or mixed-methods approaches to better evaluate the broader impact of social media-based communication efforts.

Literature Context: The discussion could be enriched by integrating more recent references (within the past 5 years) on health communication via social media, to situate the study more firmly within current discourse.

We thank the reviewer for this helpful suggestion. We have revised the Discussion section to include several more recent references (published within the past five years) on health communication via social media. These additions help to better position our study within the current research discourse.

Answer: We thank the reviewer for this helpful suggestion. We have revised the Discussion section to include several more recent references (published within the past five years) on health communication via social media. These additions help to better position our study within the current research discourse. The corresponding changes have been highlighted in the revised manuscript.

Language and Style: The manuscript is overall well-written, but some minor typographical and grammatical errors should be revised at copyediting (e.g., occasional redundancies in describing the platforms and content types). Data Availability: Statistical Rigor, Impact Assessment, Literature Context, Language and Style

We thank the reviewer for the careful reading and constructive feedback. Minor typographical and grammatical issues have been corrected throughout the manuscript. We appreciate the note that additional adjustments can be addressed during copyediting.

Overall Assessment: This manuscript makes a useful contribution to the field of health communication and respiratory medicine. With improvements in data transparency, statistical depth, and contextual discussion, it will provide even stronger evidence for the role of social media in addressing underrepresented healthcare topics.

Answer: We thank the reviewer for the careful reading and constructive feedback. We have carefully revised the manuscript to address the mentioned points. We appreciate the reviewer’s helpful comments, which have contributed to improving the overall clarity and quality of the manuscript.

Reviewer #4: This manuscript addresses an important and underexplored issue: raising public awareness of long-term invasive mechanical ventilation through social media. The study is relevant, timely, and well-presented, with a clear description of campaign activities and transparent reporting of reach metrics. Its interdisciplinary approach and innovative use of multiple media formats are commendable. However, the manuscript focuses primarily on quantitative reach without sufficiently addressing qualitative impact, such as changes in knowledge, attitudes, or behaviors among patients, relatives, or healthcare staff. The Methods section is overly descriptive in parts, while the Discussion could be strengthened by deeper analysis, including comparison with similar health communication campaigns and exploration of broader policy implications.Overall, the manuscript is valuable but requires minor revision to improve its scientific contribution and generalizability.

Answer: We thank Reviewer #4 for the positive and constructive feedback. We appreciate the recognition of the study’s relevance, interdisciplinary approach, and transparent reporting of campaign activities. We have addressed the points raised in the revised manuscript: the limitations regarding qualitative impact, generalizability, and the descriptive nature of the Methods section are now explicitly discussed. The Discussion has also been strengthened with additional context and references to similar health communication campaigns, highlighting broader implications. We are grateful for the reviewer’s suggestions, which have helped improve the scientific contribution and clarity of the manuscript.Thank you very much for pointing out the value of our manuscript and the limitations with room for improvement. The topic of generalizability was also adressed by reviewer 2 and included in the conclusion. The issue of qualitative impact was raised by reviewer 3 as well.

Attachment Submitted filename: Response to Reviewers.docx

# Peer review: Decision Letter 1

**DOI:** 10.1371/journal.pone.0352267.r003

**Authors:** Jianhong Zhou, Sulemana Bankuoru Egala

11 Jan 2026

PONE-D-25-24730R1

The use of social media in PRiVENT to raise awareness of long-term invasive ventilation

PLOS One

Dear Dr. Trudzinski,

Thank you for submitting your manuscript to PLOS ONE. After careful consideration, we feel that a significant revision has been done on the manuscript. However, there are some few outstanding issues to address in order to fully meet PLOS ONE’s publication criteria as it currently stands. Therefore, we invite you to submit a revised version of the manuscript that addresses the few points raised during the review process particularly by the second reviewer.

Please submit your revised manuscript by Feb 25 2026 11:59PM. If you will need more time than this to complete your revisions, please reply to this message or contact the journal office at plosone@plos.org. When you're ready to submit your revision, log on to https://www.editorialmanager.com/pone/ and select the 'Submissions Needing Revision' folder to locate your manuscript file.

Please include the following items when submitting your revised manuscript:

- A letter that responds to each point raised by the academic editor and reviewer(s). You should upload this letter as a separate file labeled 'Response to Reviewers'.
- A marked-up copy of your manuscript that highlights changes made to the original version. You should upload this as a separate file labeled 'Revised Manuscript with Track Changes'.
- An unmarked version of your revised paper without tracked changes. You should upload this as a separate file labeled 'Manuscript'.

-->If you would like to make changes to your financial disclosure, please include your updated statement in your cover letter. Guidelines for resubmitting your figure files are available below the reviewer comments at the end of this letter.

If applicable, we recommend that you deposit your laboratory protocols in protocols.io to enhance the reproducibility of your results. Protocols.io assigns your protocol its own identifier (DOI) so that it can be cited independently in the future. For instructions see: https://journals.plos.org/plosone/s/submission-guidelines#loc-laboratory-protocols. Additionally, PLOS ONE offers an option for publishing peer-reviewed Lab Protocol articles, which describe protocols hosted on protocols.io. Read more information on sharing protocols at https://plos.org/protocols?utm_medium=editorial-email&utm_source=authorletters&utm_campaign=protocols.

We look forward to receiving your revised manuscript.

Kind regards,

Sulemana Bankuoru Egala

Academic Editor

PLOS One

Journal Requirements:

If the reviewer comments include a recommendation to cite specific previously published works, please review and evaluate these publications to determine whether they are relevant and should be cited. There is no requirement to cite these works unless the editor has indicated otherwise.

Please review your reference list to ensure that it is complete and correct. If you have cited papers that have been retracted, please include the rationale for doing so in the manuscript text, or remove these references and replace them with relevant current references. Any changes to the reference list should be mentioned in the rebuttal letter that accompanies your revised manuscript. If you need to cite a retracted article, indicate the article’s retracted status in the References list and also include a citation and full reference for the retraction notice.

[Note: HTML markup is below. Please do not edit.]

Reviewers' comments:

Reviewer's Responses to Questions

**Comments to the Author**

1. If the authors have adequately addressed your comments raised in a previous round of review and you feel that this manuscript is now acceptable for publication, you may indicate that here to bypass the “Comments to the Author” section, enter your conflict of interest statement in the “Confidential to Editor” section, and submit your "Accept" recommendation.

Reviewer #1: (No Response)

Reviewer #3: All comments have been addressed

**********

2. Is the manuscript technically sound, and do the data support the conclusions?

The manuscript must describe a technically sound piece of scientific research with data that supports the conclusions. Experiments must have been conducted rigorously, with appropriate controls, replication, and sample sizes. The conclusions must be drawn appropriately based on the data presented.

Reviewer #1: Yes

Reviewer #3: Yes

**********

3. Has the statistical analysis been performed appropriately and rigorously?

Reviewer #1: Yes

Reviewer #3: Yes

**********

4. Have the authors made all data underlying the findings in their manuscript fully available?

The PLOS Data policy requires authors to make all data underlying the findings described in their manuscript fully available without restriction, with rare exception (please refer to the Data Availability Statement in the manuscript PDF file). The data should be provided as part of the manuscript or its supporting information, or deposited to a public repository. For example, in addition to summary statistics, the data points behind means, medians and variance measures should be available. If there are restrictions on publicly sharing data—e.g. participant privacy or use of data from a third party—those must be specified.

Reviewer #1: Yes

Reviewer #3: Yes

**********

5. Is the manuscript presented in an intelligible fashion and written in standard English?

PLOS ONE does not copyedit accepted manuscripts, so the language in submitted articles must be clear, correct, and unambiguous. Any typographical or grammatical errors should be corrected at revision, so please note any specific errors here.

Reviewer #1: Yes

Reviewer #3: Yes

**********

6. Review Comments to the Author

Please use the space provided to explain your answers to the questions above. You may also include additional comments for the author, including concerns about dual publication, research ethics, or publication ethics. (Please upload your review as an attachment if it exceeds 20,000 characters)

Reviewer #1: (No Response)

Reviewer #3: The revised manuscript has addressed the major points raised in the previous round of review satisfactorily. The overall structure is clear, the objectives are well defined, and the methodological approach is appropriate for the research questions posed.

The data analysis is coherent and adequately supports the conclusions. The discussion section successfully links the findings to existing literature, although minor strengthening of the theoretical implications would further enhance the manuscript’s contribution.

1. Minor language polishing is recommended, particularly in the Introduction and Discussion sections, to improve clarity and flow.

2. Please ensure consistency in terminology and formatting throughout the manuscript.

3. Where applicable, adding brief justifications for selected analytical parameters would improve methodological transparency.

Overall, the manuscript is scientifically sound and suitable for publication after minor revisions.

**********

7. PLOS authors have the option to publish the peer review history of their article (what does this mean?). If published, this will include your full peer review and any attached files.

If you choose “no”, your identity will remain anonymous but your review may still be made public.

**Do you want your identity to be public for this peer review?**    For information about this choice, including consent withdrawal, please see our Privacy Policy.

Reviewer #1: No

Reviewer #3: **Yes:**    Alexander Adrian Saragi

**********

[NOTE: If reviewer comments were submitted as an attachment file, they will be attached to this email and accessible via the submission site. Please log into your account, locate the manuscript record, and check for the action link "View Attachments". If this link does not appear, there are no attachment files.]

To ensure your figures meet our technical requirements, please review our figure guidelines: https://journals.plos.org/plosone/s/figures

You may also use PLOS’s free figure tool, NAAS, to help you prepare publication quality figures: https://journals.plos.org/plosone/s/figures#loc-tools-for-figure-preparation.

NAAS will assess whether your figures meet our technical requirements by comparing each figure against our figure specifications.

Attachment Submitted filename: Plos one accept.docx

# Author comment: Author response to Decision Letter 2

**DOI:** 10.1371/journal.pone.0352267.r004

12 Jan 2026

Response to Reviewers’ Comments

We thank the reviewers for their careful evaluation of our revised manuscript and for their constructive and positive feedback.

Reviewer #1

Reviewer #1 did not provide additional comments and indicated that all evaluation criteria have been satisfactorily met. We thank the reviewer for the positive assessment of our work.

Reviewer #3

We sincerely thank Reviewer #3 for the detailed review and for confirming that the major issues raised in the previous round have been adequately addressed. We respond to the remaining minor comments as follows:

Comment 1: Minor language polishing is recommended, particularly in the Introduction and Discussion sections.

Response:

The Introduction and Discussion have been carefully revised to improve clarity, flow, and readability. Minor grammatical and stylistic issues have been corrected throughout the manuscript.

Comment 2: Please ensure consistency in terminology and formatting throughout the manuscript.

Response:

The manuscript has been reviewed in its entirety to ensure consistent terminology, abbreviations, and formatting across all sections.

Comment 3: Adding brief justifications for selected analytical parameters would improve methodological transparency.

Response:

Brief justifications for key analytical parameters have been added to the Methods section where appropriate, improving transparency and clarity of the analytical approach.

We believe that these minor revisions further strengthen the manuscript and address all remaining concerns. We thank the reviewer again for the constructive comments and for considering the manuscript suitable for publication.

Attachment Submitted filename: Response_to_Reviewers_auresp_2.docx

# Peer review: Decision Letter 2

**DOI:** 10.1371/journal.pone.0352267.r005

**Authors:** Jianhong Zhou, Sulemana Bankuoru Egala, Sulemana Bankuoru Egala

18 Mar 2026

PONE-D-25-24730R2

The use of social media in PRiVENT to raise awareness of long-term invasive ventilation

PLOS One

Dear Dr. Trudzinski,

Thank you for submitting your manuscript to PLOS ONE. After careful consideration, we feel that the manuscript still falls short of the quality desired for publication in its current form. Consequently, we have decided to request another round of major revisions to enable you to thoroughly revise the manuscript to ensure it is fit for purpose.

While Reviewer 6 recommends acceptance, Reviewer 5 has raised critical concerns that are essential to address in order to make the manuscript suitable for publication in this journal. Specifically, these concerns relate to the conceptual framing and theoretical rigor, weak methodological approaches and operationalization of targets, and insufficient contributions.

Therefore, we invite you to submit a revised version of the manuscript that addresses the critical points raised during the review process, particularly those noted by Reviewer 5.

We wish to stress that this stage is very critical in accordance with the journal's standards, and we recommend that you adequately address all the issues raised by the reviewer.

Please submit your revised manuscript by May 02 2026 11:59PM. If you will need more time than this to complete your revisions, please reply to this message or contact the journal office at plosone@plos.org. When you're ready to submit your revision, log on to https://www.editorialmanager.com/pone/ and select the 'Submissions Needing Revision' folder to locate your manuscript file.

Please include the following items when submitting your revised manuscript:

- A letter that responds to each point raised by the academic editor and reviewer(s). You should upload this letter as a separate file labeled 'Response to Reviewers'.
- A marked-up copy of your manuscript that highlights changes made to the original version. You should upload this as a separate file labeled 'Revised Manuscript with Track Changes'.
- An unmarked version of your revised paper without tracked changes. You should upload this as a separate file labeled 'Manuscript'.

If you would like to make changes to your financial disclosure, please include your updated statement in your cover letter. Guidelines for resubmitting your figure files are available below the reviewer comments at the end of this letter.

If applicable, we recommend that you deposit your laboratory protocols in protocols.io to enhance the reproducibility of your results. Protocols.io assigns your protocol its own identifier (DOI) so that it can be cited independently in the future. For instructions see: https://journals.plos.org/plosone/s/submission-guidelines#loc-laboratory-protocols. Additionally, PLOS ONE offers an option for publishing peer-reviewed Lab Protocol articles, which describe protocols hosted on protocols.io. Read more information on sharing protocols at https://plos.org/protocols?utm_medium=editorial-email&utm_source=authorletters&utm_campaign=protocols.

We look forward to receiving your revised manuscript.

Kind regards,

Sulemana Bankuoru Egala

Academic Editor

PLOS One

**Journal Requirements:**

If the reviewer comments include a recommendation to cite specific previously published works, please review and evaluate these publications to determine whether they are relevant and should be cited. There is no requirement to cite these works unless the editor has indicated otherwise.

[Note: HTML markup is below. Please do not edit.]

Reviewers' comments:

Reviewer's Responses to Questions

**Comments to the Author**

1. If the authors have adequately addressed your comments raised in a previous round of review and you feel that this manuscript is now acceptable for publication, you may indicate that here to bypass the “Comments to the Author” section, enter your conflict of interest statement in the “Confidential to Editor” section, and submit your "Accept" recommendation.

Reviewer #5: (No Response)

Reviewer #6: All comments have been addressed

**********

2. Is the manuscript technically sound, and do the data support the conclusions?

The manuscript must describe a technically sound piece of scientific research with data that supports the conclusions. Experiments must have been conducted rigorously, with appropriate controls, replication, and sample sizes. The conclusions must be drawn appropriately based on the data presented.

Reviewer #5: Partly

Reviewer #6: Yes

**********

3. Has the statistical analysis been performed appropriately and rigorously?

Reviewer #5: I Don't Know

Reviewer #6: Yes

**********

4. Have the authors made all data underlying the findings in their manuscript fully available?

The PLOS Data policy requires authors to make all data underlying the findings described in their manuscript fully available without restriction, with rare exception (please refer to the Data Availability Statement in the manuscript PDF file). The data should be provided as part of the manuscript or its supporting information, or deposited to a public repository. For example, in addition to summary statistics, the data points behind means, medians and variance measures should be available. If there are restrictions on publicly sharing data—e.g. participant privacy or use of data from a third party—those must be specified.

Reviewer #5: Yes

Reviewer #6: Yes

**********

5. Is the manuscript presented in an intelligible fashion and written in standard English?

PLOS ONE does not copyedit accepted manuscripts, so the language in submitted articles must be clear, correct, and unambiguous. Any typographical or grammatical errors should be corrected at revision, so please note any specific errors here.

Reviewer #5: Yes

Reviewer #6: Yes

**********

6. Review Comments to the Author

Please use the space provided to explain your answers to the questions above. You may also include additional comments for the author, including concerns about dual publication, research ethics, or publication ethics. (Please upload your review as an attachment if it exceeds 20,000 characters)

Reviewer #5: Review of manuscript no. PONE-D-25-24730

1. Conceptual overclaim: The central problem is conceptual. The study claims to assess whether social media “raises awareness,” yet no awareness, knowledge, attitude, perception, or behavioral outcomes were measured. Only platform analytics (views, impressions, followers) were analyzed. Visibility cannot be equated with awareness or empowerment. The conclusions therefore overstate what the data support. The manuscript must either (a) narrow its claims to “digital reach and engagement,” or (b) include outcome measures demonstrating actual awareness impact.

2. Lack of theoretical framework: The manuscript is descriptive and operational but theoretically underdeveloped. There is no engagement with communication theory, health literacy frameworks, public sphere theory, digital engagement models, or knowledge translation literature. For a paper that positions itself around “public awareness,” this absence is a major weakness. The introduction should be grounded in established frameworks of science communication, digital health communication, or misinformation research.

3. Methodological limitation: purely descriptive analytics: The study design is retrospective and purely descriptive. There is no comparator (e.g., before–after, control topic, similar campaigns), no inferential analysis, no engagement rate calculation (per post, per follower), and no evaluation of content effectiveness beyond raw counts. Without normalization or benchmarking, the 2.6 million views cannot be interpreted. The analysis lacks analytical depth and risks being perceived as a campaign report rather than research.

4. Weak operationalization of target groups: The manuscript assumes that certain demographic distributions (e.g., young female Instagram users) indicate successful reach of nurses. This inference is speculative. No direct occupational verification exists. Similarly, LinkedIn professional categories are interpreted as specific healthcare roles without validation. The conclusions about reaching intended target groups are not sufficiently supported by the data.

5. Limited contribution: From a scholarly perspective, the contribution is unclear. Many health projects use social media; descriptive reporting of output volume and views is not, in itself, novel. The manuscript needs to clarify what new knowledge this study generates for digital health communication, implementation science, or public engagement research. At present, it reads as documentation of project dissemination activities.

6. Discussion lacks critical reflection: The discussion remains largely affirmative. There is minimal critical interrogation of:

• algorithmic bias.

• paid promotion effects.

• regional targeting limitations.

• sustainability of engagement.

• potential echo-chamber effects.

• cost-effectiveness.

Hence, a more rigorous critical appraisal is required.

Overall assessment: The topic is relevant and socially meaningful. The execution of the campaign appears substantial. However, as a scientific manuscript, it currently overinterprets descriptive analytics and lacks theoretical and methodological depth. Substantial revision is required to align claims with evidence and to clarify the scholarly contribution.

Reviewer #6: This paper investigates the use of social media in the PRiVENT project to raise awareness of long-term invasive ventilation. While the methodological approach is relatively basic, it is highly appropriate and sufficient for the research objective.

The manuscript's core strength lies in its significant practical application and strong social value. By addressing a critical health communication need through social media, the findings offer actionable insights for public health practitioners and related initiatives. The paper is well-structured and clearly written. Given its robust practical contribution, I recommend this manuscript for acceptance.

**********

7. PLOS authors have the option to publish the peer review history of their article (what does this mean?). If published, this will include your full peer review and any attached files.

If you choose “no”, your identity will remain anonymous but your review may still be made public.

**Do you want your identity to be public for this peer review?**    For information about this choice, including consent withdrawal, please see our Privacy Policy.

Reviewer #5: No

Reviewer #6: **Yes:**    chen jinghao

**********

[NOTE: If reviewer comments were submitted as an attachment file, they will be attached to this email and accessible via the submission site. Please log into your account, locate the manuscript record, and check for the action link "View Attachments". If this link does not appear, there are no attachment files.]

To ensure your figures meet our technical requirements, please review our figure guidelines: https://journals.plos.org/plosone/s/figures

You may also use PLOS’s free figure tool, NAAS, to help you prepare publication quality figures: https://journals.plos.org/plosone/s/figures#loc-tools-for-figure-preparation.

NAAS will assess whether your figures meet our technical requirements by comparing each figure against our figure specifications.

# Author comment: Author response to Decision Letter 3

**DOI:** 10.1371/journal.pone.0352267.r006

27 Mar 2026

Subject: Re: Manuscript PONE-D-25-24730R2 – Revision 3

Dear Sulemana Bankuoru Egala

Thank you very much for your detailed evaluation of our manuscript “The use of social media in PRiVENT to raise awareness of long-term invasive ventilation”.

We appreciate the constructive feedback and the opportunity to submit another revision. We acknowledge the critical concerns raised by Reviewer 5 regarding conceptual framing, theoretical rigor, methodological approaches, and the operationalization of targets. We will substantially revise the manuscript to address all points raised, with particular attention to strengthening the theoretical underpinnings, clarifying methodological procedures, and more explicitly demonstrating the contribution of our work. We also sincerely thank Reviewer #6, who had no further comments and recommended the manuscript for acceptance. We appreciate this positive assessment and are pleased that the practical relevance and social value of the work were recognized. We hope that the revised version meets all expectations and remains at your disposal for any further questions.

Thank you again for the opportunity to improve our manuscript. We look forward to continuing the review process.

Kind regards,

Franziska Trudzinski, MD

(on behalf of all co-authors)

Reviewer #5: Review of manuscript no. PONE-D-25-24730

1. Conceptual overclaim: The central problem is conceptual. The study claims to assess whether social media “raises awareness,” yet no awareness, knowledge, attitude, perception, or behavioral outcomes were measured. Only platform analytics (views, impressions, followers) were analyzed. Visibility cannot be equated with awareness or empowerment. The conclusions therefore overstate what the data support. The manuscript must either (a) narrow its claims to “digital reach and engagement,” or (b) include outcome measures demonstrating actual awareness impact.

Response:

We thank the reviewer for this important conceptual clarification and fully agree with the concern. Platform analytics provide information on visibility and reach, but they cannot be used to infer “awareness,” attitudes, or behavioral outcomes.

Following this recommendation, we have fundamentally revised the conceptual framing of the manuscript:

1. Terminology revised throughout:

We systematically replaced the term “raises awareness” with more accurate terms such as “visibility,” “digital reach,” and “engagement metrics.” No remaining statements suggest an awareness or empowerment effect.

2. Revised study title to reflect this conceptual correction:

The manuscript now uses the title:

“Social media activities to enhance the visibility of long-term invasive ventilation: A content and visibility analysis from the PRiVENT study.”

This title accurately reflects the methodological scope and avoids overstating the impact.

3. Adjusted aims and conclusions:

The aims, results, and conclusions now explicitly describe the study as an analysis of content and visibility patterns of PRiVENT’s social media activities. We emphasize that our findings relate to visibility rather than awareness.

4. Clarified methodological limitations:

We added a dedicated section explaining that platform analytics do not measure cognitive or behavioral change. This limitation is now explicitly acknowledged and integrated.

We appreciate the reviewer’s sharp conceptual guidance and believe that these changes significantly strengthen the accuracy, clarity, and rigor of the manuscript.

2. Lack of theoretical framework: The manuscript is descriptive and operational but theoretically underdeveloped. There is no engagement with communication theory, health literacy frameworks, public sphere theory, digital engagement models, or knowledge translation literature. For a paper that positions itself around “public awareness,” this absence is a major weakness. The introduction should be grounded in established frameworks of science communication, digital health communication, or misinformation research.

Response:

We thank the reviewer for this valuable comment. We agree that the original introduction did not sufficiently reflect relevant theoretical perspectives. To address this, we have briefly expanded the introduction to reference key concepts from digital health communication and social media engagement. These additions provide a clearer rationale for analysing visibility and reach in the context of specialised health topics. Given the descriptive nature of our dataset, we focus on frameworks that are directly applicable without overstating the theoretical scope of the study. We hope this concise integration satisfactorily addresses the reviewer’s concern.

3. Methodological limitation: purely descriptive analytics: The study design is retrospective and purely descriptive. There is no comparator (e.g., before–after, control topic, similar campaigns), no inferential analysis, no engagement rate calculation (per post, per follower), and no evaluation of content effectiveness beyond raw counts. Without normalization or benchmarking, the 2.6 million views cannot be interpreted. The analysis lacks analytical depth and risks being perceived as a campaign report rather than research.

Response:

We appreciate the reviewer’s observation regarding the descriptive nature of our analysis. Our dataset is limited to retrospective platform analytics, which do not allow for inferential comparisons, benchmarking, or content-effectiveness analyses. We have therefore added a clear statement to the Limitations section emphasising that our results represent descriptive visibility metrics only, that the view counts cannot be interpreted without normalization or comparators, and that the analysis should not be understood as an effectiveness evaluation. We hope this clarification appropriately contextualises the methodological scope of the study.

4. Weak operationalization of target groups: The manuscript assumes that certain demographic distributions (e.g., young female Instagram users) indicate successful reach of nurses. This inference is speculative. No direct occupational verification exists. Similarly, LinkedIn professional categories are interpreted as specific healthcare roles without validation. The conclusions about reaching intended target groups are not sufficiently supported by the data.

Response:

We thank the reviewer for this important comment. We agree that the platform-based demographic information does not allow us to reliably infer occupational groups, and that any interpretation regarding specific professions (e.g., nurses) would be speculative. We have therefore revised the manuscript to remove such inferences and now describe the demographic data strictly as platform-reported user distributions. The conclusions have been adjusted accordingly to state that different demographic groups were reached, without attributing these groups to specific professional roles. We hope this clarification adequately addresses the reviewer’s concern.

5. Limited contribution: From a scholarly perspective, the contribution is unclear. Many health projects use social media; descriptive reporting of output volume and views is not, in itself, novel. The manuscript needs to clarify what new knowledge this study generates for digital health communication, implementation science, or public engagement research. At present, it reads as documentation of project dissemination activities.

Response:

We appreciate the reviewer’s concern regarding the contribution of the study. We agree that many health projects use social media and that descriptive metrics alone are not novel. However, we believe the study still provides meaningful value for digital health communication and implementation practice. It documents, for the first time, how a national weaning project in Germany—focused on a highly specialised and often overlooked topic—was able to reach large and diverse audiences, including unexpected demographic groups. The findings offer empirical insight into which types of content were most accessed and where information needs appear to exist.While exploratory, this evidence can support future implementation strategies for similar specialised medical fields, where public awareness has historically been low. We have clarified this contribution in the revised manuscript and explicitly acknowledged the study’s limitations.

6. Discussion lacks critical reflection: The discussion remains largely affirmative. There is minimal critical interrogation of:

• algorithmic bias.

• paid promotion effects.

• regional targeting limitations.

• sustainability of engagement.

• potential echo-chamber effects.

• cost-effectiveness.

Hence, a more rigorous critical appraisal is required.

Response:

We thank the reviewer for these important comments. We agree that the study has several methodological and conceptual limitations, including the indirect identification of user groups, the descriptive design, and the limited ability to assess algorithmic effects, sustainability, or cost-effectiveness. We have now revised the limitations section accordingly and clearly outlined these constraints. In addition, we have strengthened the discussion to better delineate the study’s contribution—namely, empirical insights into the feasibility and reach of a collaborative digital health communication approach—and to specify where more robust research is needed. We hope that these revisions adequately address the reviewer’s concerns.

Overall assessment: The topic is relevant and socially meaningful. The execution of the campaign appears substantial. However, as a scientific manuscript, it currently overinterprets descriptive analytics and lacks theoretical and methodological depth. Substantial revision is required to align claims with evidence and to clarify the scholarly contribution.

Response:Thank you for this overall assessment. We appreciate the acknowledgement of the social relevance and the substantial execution of the campaign. We also agree that the original manuscript tended to overinterpret descriptive findings and did not sufficiently articulate its theoretical and methodological boundaries. In the revision, we have aligned all claims more closely with the available evidence, strengthened the positioning within existing frameworks of digital health communication, and clarified the exploratory nature of the analysis. We now explicitly outline the study’s scholarly contribution as well as its limitations.

Attachment Submitted filename: Point_by_point.docx

# Peer review: Decision Letter 3

**DOI:** 10.1371/journal.pone.0352267.r007

**Authors:** Jianhong Zhou, Sulemana Bankuoru Egala, Sulemana Bankuoru Egala, Sulemana Bankuoru Egala

4 May 2026

PONE-D-25-24730R3

Social media activities to enhance the visibility of long-term invasive ventilation: A content and visibility analysis from the PRiVENT study

PLOS One

Dear Dr. Franziska Trudzinski,

Thank you for submitting your manuscript to PLOS ONE. After careful consideration, we feel that it has merit but does not fully meet PLOS ONE’s publication criteria as it currently stands. Therefore, we invite you to submit a revised version of the manuscript that addresses the points raised during the review process.

Specifically, reviewer 3 acknowledges that the manuscript has improved, particularly in aligning claims with the descriptive nature of the data, and that the topic is socially relevant and underexplored. However, several issues remain that require attention before acceptance. These include: further softening of interpretative claims based solely on view counts, adding basic analytical depth (e.g., normalization of metrics, temporal or benchmarking analysis), strengthening statistical description (even within a descriptive framework), tempering inferred audience characteristics without direct evidence, clarifying the novel scholarly contribution relative to digital health communication literature, and expanding critical discussion to include algorithmic bias, paid vs. organic reach, sustainability, echo chambers, and cost-effectiveness. Minor language, structural, and data availability improvements are also requested.

Given that these revisions do not require new data collection or fundamental changes to the study design, a minor revision is sufficient to address the reviewer’s concerns.

Please submit your revised manuscript by Jun 18 2026 11:59PM. If you will need more time than this to complete your revisions, please reply to this message or contact the journal office at plosone@plos.org. When you're ready to submit your revision, log on to https://www.editorialmanager.com/pone/ and select the 'Submissions Needing Revision' folder to locate your manuscript file.

Please include the following items when submitting your revised manuscript:

- A letter that responds to each point raised by the academic editor and reviewer(s). You should upload this letter as a separate file labeled 'Response to Reviewers'.
- A marked-up copy of your manuscript that highlights changes made to the original version. You should upload this as a separate file labeled 'Revised Manuscript with Track Changes'.
- An unmarked version of your revised paper without tracked changes. You should upload this as a separate file labeled 'Manuscript'.

If you would like to make changes to your financial disclosure, please include your updated statement in your cover letter. Guidelines for resubmitting your figure files are available below the reviewer comments at the end of this letter.

If applicable, we recommend that you deposit your laboratory protocols in protocols.io to enhance the reproducibility of your results. Protocols.io assigns your protocol its own identifier (DOI) so that it can be cited independently in the future. For instructions see: https://journals.plos.org/plosone/s/submission-guidelines#loc-laboratory-protocols. Additionally, PLOS ONE offers an option for publishing peer-reviewed Lab Protocol articles, which describe protocols hosted on protocols.io. Read more information on sharing protocols at https://plos.org/protocols?utm_medium=editorial-email&utm_source=authorletters&utm_campaign=protocols.

As the corresponding author, your ORCID iD is verified in the submission system and will appear in the published article. PLOS supports the use of ORCID, and we encourage all coauthors to register for an ORCID iD and use it as well. Please encourage your coauthors to verify their ORCID iD within the submission system before final acceptance, as unverified ORCID iDs will not appear in the published article. *Only*    the individual author can complete the verification step; PLOS staff *cannot*    verify ORCID iDs on behalf of authors.

We look forward to receiving your revised manuscript.

Kind regards,

Sulemana Bankuoru Egala (PhD)

Academic Editor

PLOS One

Journal Requirements:

If the reviewer comments include a recommendation to cite specific previously published works, please review and evaluate these publications to determine whether they are relevant and should be cited. There is no requirement to cite these works unless the editor has indicated otherwise.

Please review your reference list to ensure that it is complete and correct. If you have cited papers that have been retracted, please include the rationale for doing so in the manuscript text, or remove these references and replace them with relevant current references. Any changes to the reference list should be mentioned in the rebuttal letter that accompanies your revised manuscript. If you need to cite a retracted article, indicate the article’s retracted status in the References list and also include a citation and full reference for the retraction notice.

[Note: HTML markup is below. Please do not edit.]

Reviewers' comments:

Reviewer's Responses to Questions

**Comments to the Author**

1. If the authors have adequately addressed your comments raised in a previous round of review and you feel that this manuscript is now acceptable for publication, you may indicate that here to bypass the “Comments to the Author” section, enter your conflict of interest statement in the “Confidential to Editor” section, and submit your "Accept" recommendation.

Reviewer #3: All comments have been addressed

Reviewer #6: All comments have been addressed

**********

2. Is the manuscript technically sound, and do the data support the conclusions?

The manuscript must describe a technically sound piece of scientific research with data that supports the conclusions. Experiments must have been conducted rigorously, with appropriate controls, replication, and sample sizes. The conclusions must be drawn appropriately based on the data presented.

Reviewer #3: Partly

Reviewer #6: Yes

**********

3. Has the statistical analysis been performed appropriately and rigorously?

Reviewer #3: No

Reviewer #6: Yes

**********

4. Have the authors made all data underlying the findings in their manuscript fully available?

The PLOS Data policy requires authors to make all data underlying the findings described in their manuscript fully available without restriction, with rare exception (please refer to the Data Availability Statement in the manuscript PDF file). The data should be provided as part of the manuscript or its supporting information, or deposited to a public repository. For example, in addition to summary statistics, the data points behind means, medians and variance measures should be available. If there are restrictions on publicly sharing data—e.g. participant privacy or use of data from a third party—those must be specified.

Reviewer #3: No

Reviewer #6: Yes

**********

5. Is the manuscript presented in an intelligible fashion and written in standard English?

PLOS ONE does not copyedit accepted manuscripts, so the language in submitted articles must be clear, correct, and unambiguous. Any typographical or grammatical errors should be corrected at revision, so please note any specific errors here.

Reviewer #3: Yes

Reviewer #6: Yes

**********

6. Review Comments to the Author

Please use the space provided to explain your answers to the questions above. You may also include additional comments for the author, including concerns about dual publication, research ethics, or publication ethics. (Please upload your review as an attachment if it exceeds 20,000 characters)

Reviewer #3: The manuscript addresses a socially relevant and underexplored topic, namely the role of social media in increasing the visibility of long-term invasive mechanical ventilation (IMV). The authors present a substantial communication effort through the PRiVENT project, and the dataset spans multiple years and platforms, which is commendable. The revision has improved the manuscript, particularly in aligning claims with the descriptive nature of the data. However, several critical issues remain that limit the scientific contribution of the study.

Major Comments

1. Conceptual Framing and Claims

The authors have improved the terminology by shifting from “awareness” to “visibility” and “reach,” which is appropriate. However, some residual interpretative statements still imply user interest or informational needs based solely on view counts. These interpretations should be further softened, as platform analytics cannot capture cognitive engagement, understanding, or behavioral impact.

2. Methodological Limitations and Analytical Depth

The study remains purely descriptive. While the authors acknowledge this limitation, the manuscript still lacks analytical depth expected for a research article. Specifically:

- No normalization of metrics (e.g., engagement rate per post or per follower) is provided.

- No temporal or comparative analysis (e.g., trends over time, pre/post campaign phases) is included.

- No benchmarking against similar campaigns or baseline metrics is attempted.

As a result, the reported figures (e.g., 2.6 million views) remain difficult to interpret in a meaningful scientific context.

3. Statistical Analysis

The absence of inferential or even basic comparative statistical analysis significantly weakens the rigor of the study. Even within a descriptive framework, additional analyses (e.g., distribution comparisons across platforms or content types) could strengthen the manuscript.

4. Operationalization of Target Groups

Although the authors have removed earlier overinterpretations, the discussion still occasionally suggests inferred audience characteristics (e.g., healthcare professionals, nurses) without direct evidence. Platform-derived demographic and professional data should be treated with caution and clearly labeled as indirect indicators.

5. Scholarly Contribution

The manuscript still struggles to clearly articulate its novel contribution. While documenting a large-scale communication effort is valuable, the authors should more explicitly position their findings within digital health communication literature. What transferable insights does this study offer beyond descriptive reporting? This point requires further strengthening.

6. Discussion and Critical Reflection

The discussion has improved but remains somewhat limited in critical depth. Important aspects are still underdeveloped, including:

- Algorithmic bias and platform-specific visibility mechanisms

- The role of paid promotion versus organic reach

- Sustainability of engagement over time

- Potential echo-chamber effects

- Cost-effectiveness of the campaign

A more critical and reflective discussion would significantly enhance the manuscript’s academic value.

Minor Comments

- Some grammatical inconsistencies and minor language issues remain and should be carefully proofread.

- The Methods section would benefit from clearer structuring, particularly in distinguishing between content production and analytical procedures.

- The Data Availability Statement does not fully comply with open data requirements; providing at least anonymized aggregated datasets in a repository would improve transparency.

The manuscript has improved compared to previous versions and addresses a relevant topic. However, it still requires moderate to major revision to enhance methodological rigor, analytical depth, and clarity of scholarly contribution before it can be considered suitable for publication.

Reviewer #6: Thank you for the revision. The manuscript has improved, especially by narrowing the claim from “raising awareness” to “enhancing visibility.” This better matches the available data, which mainly include views, reach, impressions, followers, and platform user profiles.

The study remains descriptive and does not directly measure awareness, knowledge change, patient outcomes, or behavioral effects. Its theoretical and methodological contributions are therefore limited. However, the manuscript documents a meaningful practice in digital health communication. The PRiVENT project produced substantial public-facing content, including blog posts, social media posts, and podcast episodes, on long-term invasive mechanical ventilation, a highly specialized and often overlooked topic.

I especially appreciate the practical value of providing plain-language information for patients, relatives, and healthcare professionals. The strong interest in basic explanatory content, such as weaning and invasive ventilation, suggests a real need for accessible information in this field.

I believe that the manuscript has now met the requirements for publication.

**********

7. PLOS authors have the option to publish the peer review history of their article (what does this mean?). If published, this will include your full peer review and any attached files.

If you choose “no”, your identity will remain anonymous but your review may still be made public.

**Do you want your identity to be public for this peer review?**    For information about this choice, including consent withdrawal, please see our Privacy Policy.

Reviewer #3: **Yes:**    Alexander Adrian Saragi

Reviewer #6: **Yes:**    chen jinghao

**********

[NOTE: If reviewer comments were submitted as an attachment file, they will be attached to this email and accessible via the submission site. Please log into your account, locate the manuscript record, and check for the action link "View Attachments". If this link does not appear, there are no attachment files.]

To ensure your figures meet our technical requirements, please review our figure guidelines: https://journals.plos.org/plosone/s/figures

You may also use PLOS’s free figure tool, NAAS, to help you prepare publication quality figures: https://journals.plos.org/plosone/s/figures#loc-tools-for-figure-preparation.

NAAS will assess whether your figures meet our technical requirements by comparing each figure against our figure specifications.

# Author comment: Author response to Decision Letter 4

**DOI:** 10.1371/journal.pone.0352267.r008

6 May 2026

Reviewer #3: The manuscript addresses a socially relevant and underexplored topic, namely the role of social media in increasing the visibility of long-term invasive mechanical ventilation (IMV). The authors present a substantial communication effort through the PRiVENT project, and the dataset spans multiple years and platforms, which is commendable. The revision has improved the manuscript, particularly in aligning claims with the descriptive nature of the data. However, several critical issues remain that limit the scientific contribution of the study.

Anwer: Thank you for your thoughtful and constructive feedback. We appreciate your recognition of the relevance of the topic and the improvements made in the revision. We have carefully addressed the remaining concerns and further clarified scope, methods, and interpretation accordingly.

Major Comments

1. Conceptual Framing and Claims

The authors have improved the terminology by shifting from “awareness” to “visibility” and “reach,” which is appropriate. However, some residual interpretative statements still imply user interest or informational needs based solely on view counts. These interpretations should be further softened, as platform analytics cannot capture cognitive engagement, understanding, or behavioral impact.

The manuscript has been thoroughly revised and the respective sections have been adjusted accordingly.

2. Methodological Limitations and Analytical Depth

The study remains purely descriptive. While the authors acknowledge this limitation, the manuscript still lacks analytical depth expected for a research article. Specifically:

- No normalization of metrics (e.g., engagement rate per post or per follower) is provided.

- No temporal or comparative analysis (e.g., trends over time, pre/post campaign phases) is included.

- No benchmarking against similar campaigns or baseline metrics is attempted.

As a result, the reported figures (e.g., 2.6 million views) remain difficult to interpret in a meaningful scientific context.

Anwer: Thank you for these important and well-founded comments. We fully agree with the limitations you raise and have addressed them more explicitly in the revised manuscript.Regarding normalization of metrics, this remains challenging, as the included platforms use fundamentally different definitions and calculation methods for reach and engagement, limiting comparability across channels. We have clarified this point in the Methods and Discussion.With respect to temporal analyses, we now explicitly refer to Figure X, which illustrates trends over time within platforms. However, we agree that more robust pre/post comparisons are not feasible: the information offering was newly established within the PRiVENT project, and no pre-campaign phase exists. In addition, the platforms are still actively maintained, so a defined post-campaign phase cannot yet be analyzed.Benchmarking against similar campaigns or baseline metrics is likewise not possible, as— to our knowledge—no comparable structured program in this field currently exists in Germany, and baseline data are lacking.We have incorporated all these aspects more clearly into the Discussion as key limitations and outlined corresponding needs for future research, particularly regarding standardized metrics, longitudinal evaluation frameworks, and comparative analyses.

3. Statistical Analysis

The absence of inferential or even basic comparative statistical analysis significantly weakens the rigor of the study. Even within a descriptive framework, additional analyses (e.g., distribution comparisons across platforms or content types) could strengthen the manuscript.

Anwer: Thank you for this important point. We agree that the absence of inferential or comparative statistical analyses limits the rigor of the study. At the same time, additional comparative analyses are only feasible to a limited extent in this context. As outlined above, the different platforms apply heterogeneous and non-transparent definitions of key metrics (e.g., views, reach, engagement), which restricts comparability across platforms and limits the validity of distributional comparisons. We have clarified this constraint more explicitly in the revised Methods and Discussion.Within these boundaries, we have strengthened the descriptive analysis where possible and now more clearly highlight temporal developments (see Figure 2). However, more advanced comparative approaches (e.g., across content types or platforms) remain methodologically challenging given the lack of standardized metrics and a consistent baseline. We have incorporated these aspects into the Discussion as important limitations and outlined the need for future research focusing on standardized reporting frameworks and more robust comparative designs.

4. Operationalization of Target Groups

Although the authors have removed earlier overinterpretations, the discussion still occasionally suggests inferred audience characteristics (e.g., healthcare professionals, nurses) without direct evidence. Platform-derived demographic and professional data should be treated with caution and clearly labeled as indirect indicators.

Anwer: Thank you for this important observation. We agree that such interpretations require caution. In the revision, we have addressed this by consistently avoiding unsupported inferences and by clearly labeling any platform-derived demographic or professional information as indirect indicators. We have further revised the Discussion to ensure that audience characteristics are not overstated and remain aligned with the descriptive nature of the data.

5. Scholarly Contribution

The manuscript still struggles to clearly articulate its novel contribution. While documenting a large-scale communication effort is valuable, the authors should more explicitly position their findings within digital health communication literature. What transferable insights does this study offer beyond descriptive reporting? This point requires further strengthening.

Anwer: hank you for this important comment. We agree that the novel contribution of the study needs to be articulated more clearly.In the revision, we have strengthened the positioning of our study within the digital health communication literature and more explicitly outlined its contribution. To the best of our knowledge, this study represents the first structured, multi-platform digital communication approach addressing the highly specialized field of long-term mechanical ventilation in Germany. As such, it should primarily be understood as a hypothesis-generating and exploratory analysis rather than a confirmatory evaluation.Beyond descriptive reporting, we highlight (i) the feasibility of developing and implementing a structured, multi-platform communication strategy for a previously underrepresented patient group, (ii) the methodological challenges related to non-standardized and non-transparent platform metrics and their implications for evaluation, and (iii) practical considerations for longitudinal, cross-platform outreach in a clinical research context.The Discussion has been revised accordingly to better emphasize these aspects and to clarify how our findings may inform the design of future studies, particularly with regard to more rigorous, comparative, and theory-driven evaluation frameworks.

6. Discussion and Critical Reflection

The discussion has improved but remains somewhat limited in critical depth. Important aspects are still underdeveloped, including:

- Algorithmic bias and platform-specific visibility mechanisms

- The role of paid promotion versus organic reach

- Sustainability of engagement over time

- Potential echo-chamber effects

- Cost-effectiveness of the campaign

A more critical and reflective discussion would significantly enhance the manuscript’s academic value.

Anwer:Thank you for this valuable and insightful feedback. We agree that these aspects are highly relevant and that a more critical and reflective discussion strengthens the manuscript.In the revision, we have expanded the Discussion accordingly. Specifically, we now address the potential influence of algorithmic bias and platform-specific visibility mechanisms, acknowledging that content reach is shaped by non-transparent algorithms. We also clarify that our analysis is based predominantly on organic reach.Furthermore, we have added a more critical reflection on the sustainability of engagement over time, referring to the temporal trends shown in Figure 2, while noting the current lack of a defined post-campaign phase due to ongoing platform activity. We also discuss the potential for echo-chamber effects, particularly in health-related social media communication, and the associated limitations in reaching broader or less engaged audiences.Regarding cost-effectiveness, we clarify in the revision that a formal evaluation of the overall PRiVENT intervention is planned. To avoid unwarranted assumptions, we deliberately refrain from providing estimates or interpretations at this stage and instead highlight this as an important area for future research.All these aspects have been incorporated into the revised Discussion as key limitations and avenues for further investigation, thereby strengthening the critical depth and contextualization of our findings.

Minor Comments

- Some grammatical inconsistencies and minor language issues remain and should be carefully proofread.

- The Methods section would benefit from clearer structuring, particularly in distinguishing between content production and analytical procedures.

- The Data Availability Statement does not fully comply with open data requirements; providing at least anonymized aggregated datasets in a repository would improve transparency.The manuscript has improved compared to previous versions and addresses a relevant topic. However, it still requires moderate to major revision to enhance methodological rigor, analytical depth, and clarity of scholarly contribution before it can be considered suitable for publication.

Anwer: We have carefully proofread the manuscript and corrected remaining grammatical inconsistencies and minor language issues. The Methods section has been restructured to more clearly distinguish between content production and analytical procedures. An anonymized, aggregated dataset has been made publicly available via Zenodo (https://zenodo.org/records/20035681)

Reviewer #6: Thank you for the revision. The manuscript has improved, especially by narrowing the claim from “raising awareness” to “enhancing visibility.” This better matches the available data, which mainly include views, reach, impressions, followers, and platform user profiles.

The study remains descriptive and does not directly measure awareness, knowledge change, patient outcomes, or behavioral effects. Its theoretical and methodological contributions are therefore limited. However, the manuscript documents a meaningful practice in digital health communication. The PRiVENT project produced substantial public-facing content, including blog posts, social media posts, and podcast episodes, on long-term invasive mechanical ventilation, a highly specialized and often overlooked topic.

I especially appreciate the practical value of providing plain-language information for patients, relatives, and healthcare professionals. The strong interest in basic explanatory content, such as weaning and invasive ventilation, suggests a real need for accessible information in this field.

I believe that the manuscript has now met the requirements for publication.

Anwer: Thank you again for your constructive feedback and for supporting the publication of our work.

Attachment Submitted filename: Point_by_point_auresp_4.docx

# Peer review: Decision Letter 4

**DOI:** 10.1371/journal.pone.0352267.r009

**Authors:** Jianhong Zhou, Sulemana Bankuoru Egala, Sulemana Bankuoru Egala, Sulemana Bankuoru Egala, Sulemana Bankuoru Egala

20 May 2026

PONE-D-25-24730R4

Social media activities to enhance the visibility of long-term invasive ventilation: A content and visibility analysis from the PRiVENT study

PLOS One

Dear Dr. Franziska Trudzinski,

Thank you for submitting your manuscript to PLOS ONE. After careful consideration, we feel that it has merit but does not fully meet PLOS ONE’s publication criteria as it currently stands. Therefore, we invite you to submit a revised version of the manuscript that addresses the points raised during the review process.

Soecifically, it is acknowledged that the manuscript addresses an important, underexplored topic (digital communication for long-term invasive mechanical ventilation and weaning) and notes that the revised version has improved substantially, particularly in the appropriate use of “visibility”/“reach” and a more balanced discussion of limitations. However, several issues require further refinement before publication. The reviewer raises four gemaine comments focused on strengthening the scholarly framing and interpretation:

1. Position the work better within digital health communication literature and articulate transferable scientific insights beyond a project report.

2. Soften claims about “unmet needs” or professional group targeting, as platform analytics cannot reliably infer motivations or identities.

3. Move beyond descriptive reporting; discuss comparative content performance or provide more structured interpretation of longitudinal data (e.g., Figure 2).

4. Better integrate digital health communication theory throughout, not just briefly in the introduction.

Other minor comments include grammatical and typographical fixes, formatting inconsistencies, an unrelated reference (#10) should be corrected, improved figure legends, and language polishing by a native English speaker.

We hope the comments will be addressed timely and adequately for consideration and publiucation.

Please submit your revised manuscript by Jul 04 2026 11:59PM. If you will need more time than this to complete your revisions, please reply to this message or contact the journal office at plosone@plos.org. When you're ready to submit your revision, log on to https://www.editorialmanager.com/pone/ and select the 'Submissions Needing Revision' folder to locate your manuscript file.

Please include the following items when submitting your revised manuscript:

- A letter that responds to each point raised by the academic editor and reviewer(s). You should upload this letter as a separate file labeled 'Response to Reviewers'.
- A marked-up copy of your manuscript that highlights changes made to the original version. You should upload this as a separate file labeled 'Revised Manuscript with Track Changes'.
- An unmarked version of your revised paper without tracked changes. You should upload this as a separate file labeled 'Manuscript'.

If you would like to make changes to your financial disclosure, please include your updated statement in your cover letter. Guidelines for resubmitting your figure files are available below the reviewer comments at the end of this letter.

If applicable, we recommend that you deposit your laboratory protocols in protocols.io to enhance the reproducibility of your results. Protocols.io assigns your protocol its own identifier (DOI) so that it can be cited independently in the future. For instructions see: https://journals.plos.org/plosone/s/submission-guidelines#loc-laboratory-protocols. Additionally, PLOS ONE offers an option for publishing peer-reviewed Lab Protocol articles, which describe protocols hosted on protocols.io. Read more information on sharing protocols at https://plos.org/protocols?utm_medium=editorial-email&utm_source=authorletters&utm_campaign=protocols.

As the corresponding author, your ORCID iD is verified in the submission system and will appear in the published article. PLOS supports the use of ORCID, and we encourage all coauthors to register for an ORCID iD and use it as well. Please encourage your coauthors to verify their ORCID iD within the submission system before final acceptance, as unverified ORCID iDs will not appear in the published article. *Only*    the individual author can complete the verification step; PLOS staff *cannot*    verify ORCID iDs on behalf of authors.

We look forward to receiving your revised manuscript.

Kind regards,

Sulemana Bankuoru Egala (PhD)

Academic Editor

PLOS One

Journal Requirements:

If the reviewer comments include a recommendation to cite specific previously published works, please review and evaluate these publications to determine whether they are relevant and should be cited. There is no requirement to cite these works unless the editor has indicated otherwise.

Please review your reference list to ensure that it is complete and correct. If you have cited papers that have been retracted, please include the rationale for doing so in the manuscript text, or remove these references and replace them with relevant current references. Any changes to the reference list should be mentioned in the rebuttal letter that accompanies your revised manuscript. If you need to cite a retracted article, indicate the article’s retracted status in the References list and also include a citation and full reference for the retraction notice.

[Note: HTML markup is below. Please do not edit.]

Reviewers' comments:

Reviewer's Responses to Questions

**Comments to the Author**

1. If the authors have adequately addressed your comments raised in a previous round of review and you feel that this manuscript is now acceptable for publication, you may indicate that here to bypass the “Comments to the Author” section, enter your conflict of interest statement in the “Confidential to Editor” section, and submit your "Accept" recommendation.

Reviewer #3: All comments have been addressed

Reviewer #4: All comments have been addressed

Reviewer #6: All comments have been addressed

Reviewer #7: All comments have been addressed

**********

2. Is the manuscript technically sound, and do the data support the conclusions?

The manuscript must describe a technically sound piece of scientific research with data that supports the conclusions. Experiments must have been conducted rigorously, with appropriate controls, replication, and sample sizes. The conclusions must be drawn appropriately based on the data presented.

Reviewer #3: Yes

Reviewer #4: Yes

Reviewer #6: Yes

Reviewer #7: Yes

**********

3. Has the statistical analysis been performed appropriately and rigorously?

Reviewer #3: Yes

Reviewer #4: Yes

Reviewer #6: Yes

Reviewer #7: Yes

**********

4. Have the authors made all data underlying the findings in their manuscript fully available?

The PLOS Data policy requires authors to make all data underlying the findings described in their manuscript fully available without restriction, with rare exception (please refer to the Data Availability Statement in the manuscript PDF file). The data should be provided as part of the manuscript or its supporting information, or deposited to a public repository. For example, in addition to summary statistics, the data points behind means, medians and variance measures should be available. If there are restrictions on publicly sharing data—e.g. participant privacy or use of data from a third party—those must be specified.

Reviewer #3: Yes

Reviewer #4: Yes

Reviewer #6: Yes

Reviewer #7: Yes

**********

5. Is the manuscript presented in an intelligible fashion and written in standard English?

PLOS ONE does not copyedit accepted manuscripts, so the language in submitted articles must be clear, correct, and unambiguous. Any typographical or grammatical errors should be corrected at revision, so please note any specific errors here.

Reviewer #3: Yes

Reviewer #4: Yes

Reviewer #6: Yes

Reviewer #7: Yes

**********

6. Review Comments to the Author

Please use the space provided to explain your answers to the questions above. You may also include additional comments for the author, including concerns about dual publication, research ethics, or publication ethics. (Please upload your review as an attachment if it exceeds 20,000 characters)

Reviewer #3: The revised manuscript has improved substantially compared with previous versions. The authors have responded carefully and constructively to the major concerns raised during the earlier review rounds, particularly by refining the conceptual framing from “awareness” to “visibility” and “reach,” which is more consistent with the descriptive nature of the available platform metrics.

The manuscript now presents a clearer and more balanced interpretation of the findings. The authors appropriately acknowledge that platform analytics cannot directly measure cognitive engagement, knowledge acquisition, or behavioral change. The expanded discussion of methodological limitations, including the heterogeneity of platform metrics, lack of standardized benchmarking, absence of a pre-intervention baseline, and limited comparability across platforms, significantly strengthens the scientific transparency of the study.

The restructuring of the Methods section has also improved readability, especially the clearer distinction between content production and analytical procedures. In addition, the public availability of anonymized aggregated data through Zenodo enhances the transparency and reproducibility of the work.

Although the study remains primarily descriptive and lacks inferential statistical analyses, the authors now position the manuscript appropriately as an exploratory and hypothesis-generating contribution within digital health communication research. The expanded discussion addressing algorithmic bias, sustainability of engagement, echo-chamber effects, and the limitations of platform-derived demographic indicators further improves the manuscript’s scholarly value.

The topic itself is socially relevant and underexplored. The manuscript documents an extensive and sustained multi-platform communication initiative focused on long-term invasive mechanical ventilation, a field that remains underrepresented in public health communication. The findings may provide useful practical insights for future digital communication strategies in specialized healthcare domains.

Only minor editorial polishing may still be beneficial prior to publication, particularly with regard to small grammatical inconsistencies and formatting details. Overall, however, I believe the manuscript is now suitable for publication.

Reviewer #4: SOCIAL MEDIA ACTIVITIES TO ENHANCE THE VISIBILITY OF LONG-TERM INVASIVE VENTILATION: A CONTENT AND VISIBILITY ANALYSIS FROM THE PRIVENT STUDY

The manuscript addresses an important and underexplored topic, namely the use of social media and digital communication strategies to enhance the visibility of long-term invasive mechanical ventilation (IMV) and ventilator weaning. The PRiVENT project represents a substantial multi-platform communication effort conducted over several years and involving different stakeholder groups. The topic is socially relevant and the manuscript provides valuable practical insights into digital health communication in a highly specialized medical field.

The revised version has improved considerably compared with earlier versions, particularly through the more appropriate use of the terms “visibility” and “reach” instead of “awareness,” which better reflects the descriptive nature of the available data. The authors also provide a more balanced discussion of the study limitations.

However, several issues still require further refinement before publication.

Major comments

1. Clarification of scholarly contribution

Although the manuscript documents a meaningful communication initiative, the scientific contribution remains somewhat unclear. The study is primarily descriptive and resembles a project report in several sections. The authors should further strengthen the positioning of the manuscript within the broader digital health communication literature and more explicitly explain what transferable scientific insights can be derived from this work beyond reporting platform metrics and content dissemination activities.

2. Overinterpretation of platform metrics

The manuscript has improved substantially in avoiding unsupported claims; however, some statements still imply audience needs or audience composition without direct evidence. For example, statements suggesting that the observed access patterns indicate an “unmet need” or that specific professional groups were reached should be softened further. Platform analytics and view counts cannot reliably establish user motivations, informational needs, or professional identities. More cautious wording such as “may suggest” or “could reflect” would be more appropriate.

3. Limited analytical depth

The analysis remains largely descriptive. While the limitations of platform-specific metrics are acknowledged, the manuscript would benefit from additional analytical depth where feasible. Even within a descriptive framework, the authors could further discuss comparative performance across content categories or provide more structured interpretation of longitudinal developments shown in Figure 2. At present, the manuscript relies heavily on absolute numbers without deeper contextual interpretation.

4. Theoretical framing

The introduction would benefit from stronger integration of digital health communication theory. The manuscript briefly refers to digital engagement and health information seeking, but these concepts are not sufficiently developed throughout the paper. A clearer theoretical framing would strengthen the academic value of the study and help contextualize the findings within existing communication research.

Minor comments

1. Several grammatical inconsistencies and typographical issues remain throughout the manuscript and should be carefully proofread.

2. There are occasional formatting inconsistencies, including spacing and punctuation issues.

3. A clear reference mismatch appears in reference 10 (“Partial crowns as bridge abutments”), which seems unrelated to the topic of the manuscript and should be corrected.

4. Some figure legends could be improved for clarity and readability.

5. The manuscript would benefit from minor language polishing by a native English speaker or professional editing service.

Overall assessment

Overall, this manuscript addresses a relevant and specialized topic and provides useful practical insights into digital communication strategies in the field of long-term invasive mechanical ventilation. While the study remains primarily descriptive and has limited analytical depth, the revisions have substantially improved the manuscript’s balance and transparency. With additional clarification of the scholarly contribution, careful reduction of residual overinterpretation, and minor editorial corrections, the manuscript would be suitable for publication.

Reviewer #6: The manuscript has improved, particularly in aligning the claims with the available data and in clarifying that platform metrics reflect visibility and reach rather than awareness, knowledge change, or behavioral impact. The study remains mainly descriptive, and its theoretical and methodological contribution is limited by the absence of standardized platform metrics, inferential analysis, and direct measures of audience engagement or outcomes. However, the topic is important, underrepresented, and socially meaningful. The manuscript provides useful documentation of a structured, multi-platform communication effort in the field of long-term invasive mechanical ventilation, with practical relevance for digital health communication, patients, relatives, and healthcare professionals.

Reviewer #7: The use of social media in PRiVENT to raise awareness of long-term invasive

ventilation

I thank the authors of this work for the important task provided with the PRIVENT project, about information spreading on long-term invasive mechanical ventilation.

I have seen the work and its progress across the multiple revisions. The authors have done a great job improving the content a lot by following the reviewers' suggestions in this 4-stage revision.

I think that now the manuscript is ready for acceptance and publication.

**********

7. PLOS authors have the option to publish the peer review history of their article (what does this mean?). If published, this will include your full peer review and any attached files.

If you choose “no”, your identity will remain anonymous but your review may still be made public.

**Do you want your identity to be public for this peer review?**    For information about this choice, including consent withdrawal, please see our Privacy Policy.

Reviewer #3: **Yes:**    Alexander Adrian Saragi

Reviewer #4: No

Reviewer #6: **Yes:**    chen jinghao

Reviewer #7: No

**********

[NOTE: If reviewer comments were submitted as an attachment file, they will be attached to this email and accessible via the submission site. Please log into your account, locate the manuscript record, and check for the action link "View Attachments". If this link does not appear, there are no attachment files.]

To ensure your figures meet our technical requirements, please review our figure guidelines: https://journals.plos.org/plosone/s/figures

You may also use PLOS’s free figure tool, NAAS, to help you prepare publication quality figures: https://journals.plos.org/plosone/s/figures#loc-tools-for-figure-preparation.

NAAS will assess whether your figures meet our technical requirements by comparing each figure against our figure specifications.

Attachment Submitted filename: Plos One social media.docx

# Author comment: Author response to Decision Letter 5

**DOI:** 10.1371/journal.pone.0352267.r010

22 May 2026

SOCIAL MEDIA ACTIVITIES TO ENHANCE THE VISIBILITY OF LONG-TERM INVASIVE VENTILATION: A CONTENT AND VISIBILITY ANALYSIS FROM THE PRIVENT STUDY

The manuscript addresses an important and underexplored topic, namely the use of social media and digital communication strategies to enhance the visibility of long-term invasive mechanical ventilation (IMV) and ventilator weaning. The PRiVENT project represents a substantial multi-platform communication effort conducted over several years and involving different stakeholder groups. The topic is socially relevant and the manuscript provides valuable practical insights into digital health communication in a highly specialized medical field.

The revised version has improved considerably compared with earlier versions, particularly through the more appropriate use of the terms “visibility” and “reach” instead of “awareness,” which better reflects the descriptive nature of the available data. The authors also provide a more balanced discussion of the study limitations.

However, several issues still require further refinement before publication.

Response:

We sincerely thank the reviewer for the positive and encouraging assessment of our work. We greatly appreciate the recognition of the relevance of the topic, the substantial communication efforts within the PRiVENT project, and the practical contribution of the study to the field of digital health communication. We are also grateful for the acknowledgment that the revised manuscript has improved. We have carefully considered the remaining comments and addressed each point in detail below. We believe that these additional revisions have further strengthened the manuscript and improved its clarity and scientific rigor.

Major comments

1. Clarification of scholarly contribution

Although the manuscript documents a meaningful communication initiative, the scientific contribution remains somewhat unclear. The study is primarily descriptive and resembles a project report in several sections. The authors should further strengthen the positioning of the manuscript within the broader digital health communication literature and more explicitly explain what transferable scientific insights can be derived from this work beyond reporting platform metrics and content dissemination activities.

Response:We thank the reviewer for this valuable comment. We agree that the primary purpose of the study is descriptive and that the manuscript reports the implementation and performance of a multi-platform communication strategy within the PRiVENT project. To better highlight the scientific contribution beyond the reporting of communication metrics, we have revised the Discussion section and more explicitly positioned our findings within the context of digital health communication research.In particular, we now emphasize that our findings provide transferable insights into the implementation of digital communication strategies in highly specialised healthcare settings. The analysis demonstrates that different social media platforms reach distinct stakeholder groups and generate different patterns of engagement, suggesting that a coordinated multi-channel approach may be required to effectively communicate complex healthcare topics to patients, relatives, healthcare professionals, researchers, and the general public. We further discuss how these observations may inform the design of future dissemination and stakeholder engagement strategies in healthcare research projects and specialised medical disciplines.

Accordingly, the following text has been added to the Discussion "Beyond documenting communication metrics, our findings provide practical insights into the implementation of digital communication strategies in highly specialised healthcare settings. The observed differences in reach and engagement across platforms suggest that a coordinated multi-channel approach may be necessary to effectively address the diverse information needs of patients, relatives, healthcare professionals, researchers, and the broader public. These observations contribute to the growing literature on digital health communication by illustrating how different communication channels can complement each other when disseminating complex healthcare topics and engaging diverse stakeholder groups in a real-world setting."

2. Overinterpretation of platform metrics

The manuscript has improved substantially in avoiding unsupported claims; however, some statements still imply audience needs or audience composition without direct evidence. For example, statements suggesting that the observed access patterns indicate an “unmet need” or that specific professional groups were reached should be softened further. Platform analytics and view counts cannot reliably establish user motivations, informational needs, or professional identities. More cautious wording such as “may suggest” or “could reflect” would be more appropriate.

Response: We thank the reviewer for this important comment and agree that platform analytics do not allow firm conclusions regarding user motivations, informational needs, or audience composition. We have therefore carefully revised the manuscript and adopted more cautious wording throughout. Statements implying certainty about audience characteristics or unmet informational needs have been softened, and demographic information derived from social media analytics is now explicitly described as platform-provided audience estimates rather than confirmed user characteristics. These revisions ensure that the interpretation remains fully consistent with the descriptive nature and limitations of the available data.

3. Limited analytical depth

The analysis remains largely descriptive. While the limitations of platform-specific metrics are acknowledged, the manuscript would benefit from additional analytical depth where feasible. Even within a descriptive framework, the authors could further discuss comparative performance across content categories or provide more structured interpretation of longitudinal developments shown in Figure 2. At present, the manuscript relies heavily on absolute numbers without deeper contextual interpretation.

Response:We thank the reviewer for this valuable suggestion. We agree that, despite the descriptivenature of the study, additional contextual interpretation can help readers better understand the observed communication patterns.

In response, we have expanded the Results and Discussion sections to provide a more structured interpretation of the longitudinal developments shown in Figure 2 and the performance of different communication channels. Specifically, we now discuss that visibility was not distributed evenly over time but was characterised by several pronounced peaks, likely reflecting periods of intensified communication activity, publication-related dissemination, or particularly engaging content. We further highlight the complementary roles of different platforms, with Facebook and Instagram generating the highest overall reach, whereas LinkedIn contributed more consistently to professional visibility. Given the exploratory and descriptive design of the study, we deliberately refrained from conducting inferential analyses that would exceed the explanatory power of the available platform metrics. Nevertheless, the additional interpretation provides greater context for the observed trends while remaining consistent with the descriptive nature of the data.

4. Theoretical framing

The introduction would benefit from stronger integration of digital health communication theory. The manuscript briefly refers to digital engagement and health information seeking, but these concepts are not sufficiently developed throughout the paper. A clearer theoretical framing would strengthen the academic value of the study and help contextualize the findings within existing communication research.

Response:We thank the reviewer for this valuable suggestion and agree that a clearer theoretical framing strengthens the interpretation and contextualisation of our findings. In response, we have expanded the Introduction to more explicitly position the study within the field of digital health communication.Specifically, we now discuss the role of social media as a tool for health information dissemination, stakeholder engagement, and increasing the visibility of healthcare topics. We further introduce concepts of health information seeking and digital engagement to provide a conceptual framework for understanding how different digital platforms may contribute to the communication of specialised healthcare topics and reach diverse audiences. These additions help place the PRiVENT communication activities within the broader digital health communication literature and provide a clearer theoretical context for the interpretation of the observed visibility, reach, and engagement patterns.

Minor comments

1. Several grammatical inconsistencies and typographical issues remain throughout the manuscript and should be carefully proofread.

2. There are occasional formatting inconsistencies, including spacing and punctuation issues.

Response: We thank the reviewer for these comments. The manuscript has been carefully proofread and revised to address grammatical inconsistencies, typographical errors, as well as minor formatting issues, including spacing and punctuation. These revisions were made throughout the manuscript to improve consistency, clarity, and readability.

3. A clear reference mismatch appears in reference 10 (“Partial crowns as bridge abutments”), which seems unrelated to the topic of the manuscript and should be corrected.

Response: We thank the reviewer for identifying this error. Reference 10 was incorrectly listed due to a citation management issue and was unrelated to the topic of the manuscript. The reference has been corrected in the revised version, and we have carefully checked the reference list to ensure the accuracy and consistency of all citations.

4. Some figure legends could be improved for clarity and readability.

Response:We thank the reviewer for this helpful suggestion. We have revised the figure legends to improve clarity and readability. Additional explanatory information has been provided where appropriate, and the wording has been refined to facilitate interpretation of the figures without requiring extensive reference to the main text.

5. The manuscript would benefit from minor language polishing by a native English speaker or professional editing service.

Response:We thank the reviewer for this suggestion. The manuscript has been carefully proofread and comprehensively revised. Particular attention was paid to language, style, grammar, consistency, and readability throughout the text. We believe that these revisions have substantially improved the overall quality and clarity of the manuscript.

Overall assessment

Overall, this manuscript addresses a relevant and specialized topic and provides useful practical insights into digital communication strategies in the field of long-term invasive mechanical ventilation. While the study remains primarily descriptive and has limited analytical depth, the revisions have substantially improved the manuscript’s balance and transparency. With additional clarification of the scholarly contribution, careful reduction of residual overinterpretation, and minor editorial corrections, the manuscript would be suitable for publication.

Response:We sincerely thank the reviewer for the careful evaluation of our manuscript and for the constructive comments provided throughout the review process. We appreciate the recognition of the relevance of the topic and the practical contribution of the study to the field of digital health communication. We have carefully addressed all remaining comments by further clarifying the scholarly contribution of the work, adopting more cautious interpretations of platform metrics where appropriate, strengthening the theoretical framing, and implementing the suggested editorial corrections. We believe that these revisions have further improved the clarity, transparency, and overall quality of the manuscript, and we are grateful for the reviewer’s positive assessment and recommendation for publication.

Attachment Submitted filename: Plos One social media point by point.docx

# Peer review: Decision Letter 5

**DOI:** 10.1371/journal.pone.0352267.r011

**Authors:** Jianhong Zhou, Sulemana Bankuoru Egala, Sulemana Bankuoru Egala, Sulemana Bankuoru Egala, Sulemana Bankuoru Egala, Sulemana Bankuoru Egala

8 Jun 2026

Social media activities to enhance the visibility of long-term invasive ventilation: A content and visibility analysis from the PRiVENT study

PONE-D-25-24730R5

Dear Dr. Franziska Trudzinski,

We’re pleased to inform you that your manuscript has been judged scientifically suitable for publication and will be formally accepted for publication once it meets all outstanding technical requirements.

Within one week, you’ll receive an e-mail detailing the required amendments. When these have been addressed, you’ll receive a formal acceptance letter and your manuscript will be scheduled for publication.

An invoice will be generated when your article is formally accepted. Please note, if your institution has a publishing partnership with PLOS and your article meets the relevant criteria, all or part of your publication costs will be covered. Please make sure your user information is up-to-date by logging into Editorial Manager at Editorial Manager® and clicking the ‘Update My Information' link at the top of the page. For questions related to billing, please contact billing support.

If your institution or institutions have a press office, please notify them about your upcoming paper to help maximize its impact. If they’ll be preparing press materials, please inform our press team as soon as possible -- no later than 48 hours after receiving the formal acceptance. Your manuscript will remain under strict press embargo until 2 pm Eastern Time on the date of publication. For more information, please contact onepress@plos.org.

Kind regards,

Sulemana Bankuoru Egala (PhD)

Academic Editor

PLOS One

Additional Editor Comments (optional):

Reviewers' comments:

Reviewer's Responses to Questions

**Comments to the Author**

1. If the authors have adequately addressed your comments raised in a previous round of review and you feel that this manuscript is now acceptable for publication, you may indicate that here to bypass the “Comments to the Author” section, enter your conflict of interest statement in the “Confidential to Editor” section, and submit your "Accept" recommendation.

Reviewer #3: All comments have been addressed

Reviewer #6: All comments have been addressed

Reviewer #7: All comments have been addressed

**********

2. Is the manuscript technically sound, and do the data support the conclusions?

The manuscript must describe a technically sound piece of scientific research with data that supports the conclusions. Experiments must have been conducted rigorously, with appropriate controls, replication, and sample sizes. The conclusions must be drawn appropriately based on the data presented.

Reviewer #3: Yes

Reviewer #6: Yes

Reviewer #7: Yes

**********

3. Has the statistical analysis been performed appropriately and rigorously?

Reviewer #3: Yes

Reviewer #6: Yes

Reviewer #7: Yes

**********

4. Have the authors made all data underlying the findings in their manuscript fully available?

The PLOS Data policy requires authors to make all data underlying the findings described in their manuscript fully available without restriction, with rare exception (please refer to the Data Availability Statement in the manuscript PDF file). The data should be provided as part of the manuscript or its supporting information, or deposited to a public repository. For example, in addition to summary statistics, the data points behind means, medians and variance measures should be available. If there are restrictions on publicly sharing data—e.g. participant privacy or use of data from a third party—those must be specified.

Reviewer #3: Yes

Reviewer #6: Yes

Reviewer #7: Yes

**********

5. Is the manuscript presented in an intelligible fashion and written in standard English?

PLOS ONE does not copyedit accepted manuscripts, so the language in submitted articles must be clear, correct, and unambiguous. Any typographical or grammatical errors should be corrected at revision, so please note any specific errors here.

Reviewer #3: Yes

Reviewer #6: Yes

Reviewer #7: Yes

**********

6. Review Comments to the Author

Please use the space provided to explain your answers to the questions above. You may also include additional comments for the author, including concerns about dual publication, research ethics, or publication ethics. (Please upload your review as an attachment if it exceeds 20,000 characters)

Reviewer #3: Thank you for the thorough revision of the manuscript and for carefully addressing the comments raised during the previous review rounds.

The revised version demonstrates substantial improvement in several important areas. The authors have strengthened the theoretical framing of digital health communication, clarified the scientific contribution of the study, and adopted more cautious interpretations regarding platform analytics and audience characteristics. The discussion now better reflects the descriptive nature of the available data and appropriately acknowledges the limitations associated with visibility and reach metrics.

I also appreciate the additional contextual interpretation of longitudinal trends, the improved figure legends, correction of the reference inconsistencies, and the overall enhancement of language clarity and manuscript organization. The availability of an anonymized dataset through a public repository further improves transparency and reproducibility.

While the study remains primarily descriptive, the manuscript now provides a valuable real-world example of how a coordinated multi-platform communication strategy can be implemented in a highly specialized healthcare setting. The conclusions are appropriately aligned with the presented evidence and avoid unsupported claims regarding awareness or behavioral impact.

Overall, I believe that the authors have adequately addressed the concerns raised in previous review rounds. The manuscript is technically sound, clearly presented, and suitable for publication in its current form.

I have no further comments.

Reviewer #6: The manuscript has undergone several rounds of revision, and the current version shows clear improvement. The authors have adequately addressed the major concerns raised previously. I do not have further substantive comments.

Reviewer #7: The use of social media in PRiVENT to raise awareness of long-term invasive

ventilation

I thank the authors of this work for the important task provided with the PRIVENT project, about information spreading on long-term invasive mechanical ventilation.

I have seen the work and its progress across the multiple revisions. The authors have done a great job improving the content a lot by following the reviewers' suggestions in this 5-stage revision.

I think that now the manuscript is ready for acceptance and publication.

**********

7. PLOS authors have the option to publish the peer review history of their article (what does this mean?). If published, this will include your full peer review and any attached files.

If you choose “no”, your identity will remain anonymous but your review may still be made public.

**Do you want your identity to be public for this peer review?**    For information about this choice, including consent withdrawal, please see our Privacy Policy.

Reviewer #3: **Yes:**    Alexander Adrian Saragi

Reviewer #6: **Yes:**    chen jinghao

Reviewer #7: No

**********

# Editor report: Acceptance letter

**DOI:** 10.1371/journal.pone.0352267.r012

**Authors:** Jianhong Zhou, Sulemana Bankuoru Egala, Sulemana Bankuoru Egala, Sulemana Bankuoru Egala, Sulemana Bankuoru Egala, Sulemana Bankuoru Egala

PONE-D-25-24730R5

PLOS One

Dear Dr. Trudzinski,

I'm pleased to inform you that your manuscript has been deemed suitable for publication in PLOS One. Congratulations! Your manuscript is now being handed over to our production team.

At this stage, our production department will prepare your paper for publication. This includes ensuring the following:

* All references, tables, and figures are properly cited

* All relevant supporting information is included in the manuscript submission,

* There are no issues that prevent the paper from being properly typeset

You will receive further instructions from the production team, including instructions on how to review your proof when it is ready. Please keep in mind that we are working through a large volume of accepted articles, so please give us a few days to review your paper and let you know the next and final steps.

Lastly, if your institution or institutions have a press office, please let them know about your upcoming paper now to help maximize its impact. If they'll be preparing press materials, please inform our press team within the next 48 hours. Your manuscript will remain under strict press embargo until 2 pm Eastern Time on the date of publication. For more information, please contact onepress@plos.org.

You will receive an invoice from PLOS for your publication fee after your manuscript has reached the completed accept phase. If you receive an email requesting payment before acceptance or for any other service, this may be a phishing scheme. Learn how to identify phishing emails and protect your accounts at https://explore.plos.org/phishing.

If we can help with anything else, please email us at customercare@plos.org.

Thank you for submitting your work to PLOS ONE and supporting open access.

Kind regards,

PLOS ONE Editorial Office Staff

on behalf of

Prof. Sulemana Bankuoru Egala

Academic Editor

PLOS One
